# Carbene–Metal–Amide Materials Design: Tailoring *π*‐Extended Amides for High‐Performance Organic Light‐Emitting Diodes

**DOI:** 10.1002/advs.202516582

**Published:** 2025-11-14

**Authors:** Alexander C. Brannan, Jeoungmin Ji, Nguyen Le Phuoc, Donggyun Lee, Junho Kim, Mikko Linnolahti, Seunghyup Yoo, Alexander S. Romanov

**Affiliations:** ^1^ Department of Chemistry The University of Manchester Oxford Rd. Manchester M13 9PL United Kingdom; ^2^ School of Electrical Engineering Korea Advanced Institute of Science and Technology (KAIST) Daejeon 34141 Republic of Korea; ^3^ Department of Chemistry and Sustainable Technology University of Eastern Finland Joensuu Campus Joensuu FI‐80101 Finland; ^4^ Graduate School of Semiconductor Technology Korea Advanced Institute of Science and Technology (KAIST) Daejeon 34141 Republic of Korea

**Keywords:** carbene complex, gold, photoluminescence, OLED, TADF

## Abstract

A series of gold‐centred carbene‐metal‐amide (CMA) complexes are prepared with a rigid benzofuroindole (BFI) and benzothioindole (BTI) amide donor ligands coordinated with bicyclic(alkyl)amino carbene (BiC)Au(I)‐moiety in high yields. CMA complexes emit in the sky‐blue range at 501 and 511 nm with unity quantum yields in amorphous solid state media. Both CMA complexes emit thermally activated delayed fluorescence (TADF) originating from a charge transfer (CT) state with an excited state lifetime as short as 473 ns, resulting in fast radiative rates of 2 × 10^6^  s^−1^. The impact on the photoluminescence properties of the electron‐donating oxygen and sulfur atoms in amide‐donor ligand has been interpreted with the help of steady‐state and transient photo and electroluminescence experiments. Theoretical investigations revealed two charge‐transfer (T_1_ and T_2_) states followed by a locally excited T_3_ triplet state, and their spin–orbit coupling analysis. Sky‐blue host‐guest and host‐free organic light‐emitting diode (OLED) devices are fabricated with electroluminescence at ≈490 nm and practical external quantum efficiencies up to 23% with colour coordinates CIE (x; y) = 0.19; 0.32. The superior performance and operating stability of the neat OLEDs are correlated with the presence of the heavier sulfur atom, suggesting further molecular design modifications toward stable CMA OLED devices with a simplified architecture of the emitting layer (host‐free OLED).

## Graphical abstract

Sky‐blue and green thermally activated delayed fluorescent emitters, carbene–gold–amides (CMA), possess near unity photoluminescent quantum yields and high radiative rates up to 2.1 × 10^6^ s^−1^. Organic light‐emitting diodes (OLED) emit sky‐blue electroluminescence with efficiency over 20%, including host‐free OLED architecture, with operating stability LT_90_ of 4.3 h at practical brightness 100 cd m^−2^.

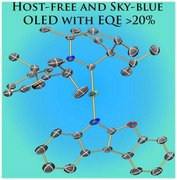

## 1 Introduction

**Scheme 1:**
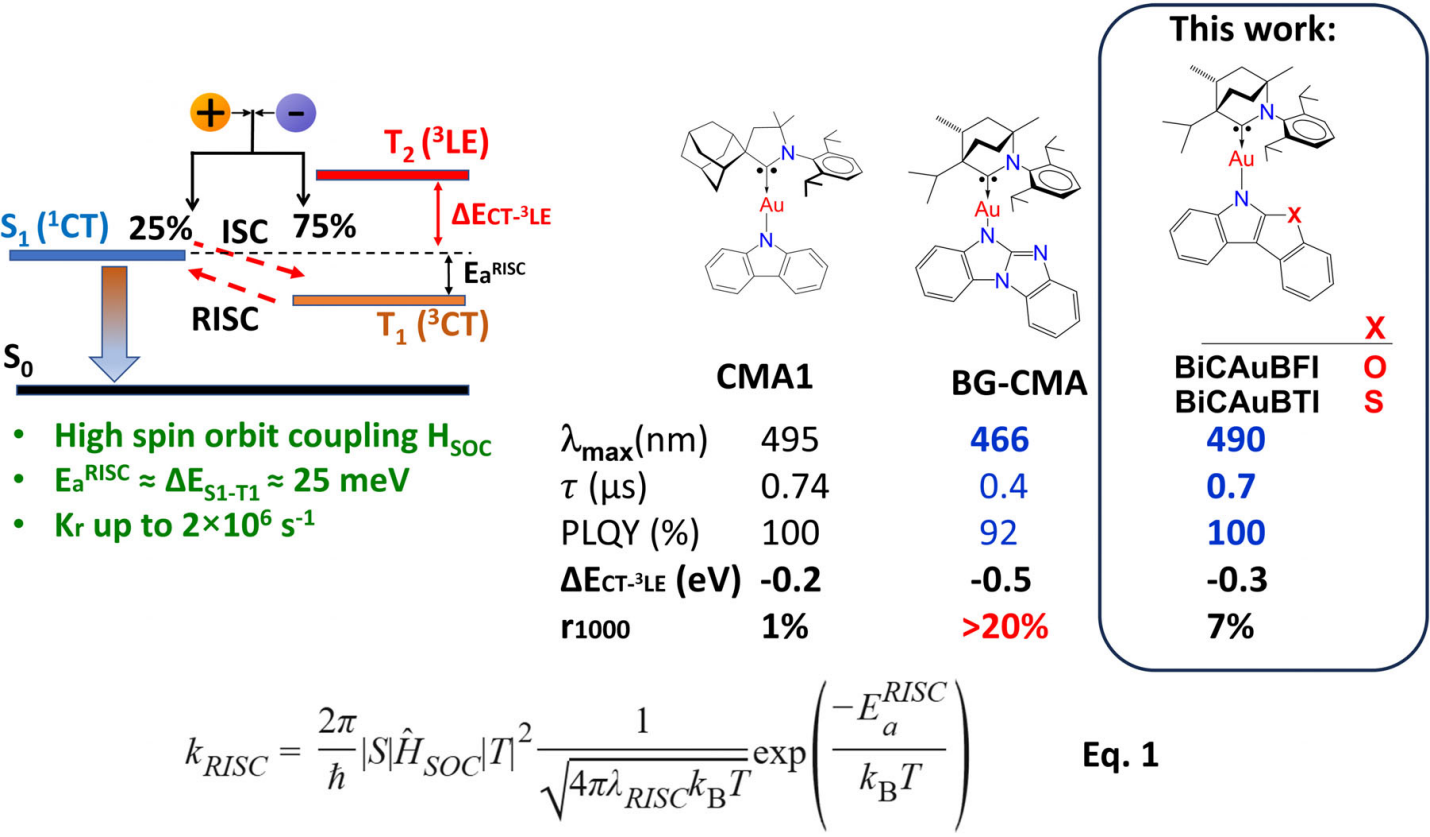
Energy state diagram for electroluminescence and representative examples of the CMA emitters with key photophysical parameters and OLED EQE efficiency roll‐off at 1000 cd m^−2^ (**r_1000_**). Equation 1 describes k_RISC_ rate where *<*S|*H*_SOC_|T*>* represents the spin–orbit coupling (HSOC) matrix element, Δ*E*_a_^RISC^ activation energy of RISC, which is similar to the energy gap between the lowest excited singlet (S_1_) and triplet (T_1_) states, λ_RISC_ is the reorganization energy, ℏ indicates the reduced Planck constant, *k*_B_ is the Boltzmann constant, and T refers to the absolute temperature.

Organometallic materials have played a pivotal role in the evolution of the Organic Light Emitting Diode (OLED) technology, enabling advanced display and lighting devices.^[^ ^1^ ^]^ Coinage metal‐based luminophores, thanks to their d^10^‐electron configuration for copper(I), silver(I), or gold(I), appeared to be particularly promising by mitigating the problem of the non‐radiative processes associated with the dark d–d^*^ transitions.^[^ ^2^, ^3^, ^4^, ^5^ ^]^ Large spin–orbit coupling of the coinage metal enables full harvesting of singlet and triplet excited states, resulting in unity photoluminescence quantum yields of phosphorescence or thermally activated delayed fluorescence (TADF).^[^ ^2^, ^3^, ^4^, ^5^ ^]^ A particular class of linear coinage metal complexes, termed as Carbene–Metal–Amide (CMA) materials, enabled fabrication of the highly energy‐efficient OLEDs with external quantum efficiencies (EQEs) over 20% at practical display brightness of 1000 cd m^−2^ by both solution and vacuum‐processed approaches.^[^ ^6^, ^7^, ^8^, ^9^, ^10^, ^11^, ^12^, ^13^ ^]^ CMA materials show leading photophysical properties among organometallic TADF emitters such as unity photoluminscence quantum yields and submicrosecond excited state lifetime across the visible range (**Scheme** **1**), however the device stability remains the key parameter to improve.^[^ ^8^, ^13^, ^14^ ^]^ In particular, it has previously been reported that operational stability is primarily compromised by bimolecular degradation pathways with devices employing CMA emitters, through exciton–exciton annihilation processes at high current densities.^[^ ^15^ ^]^ It was suggested that such a degradation pathway can be mitigated by lowering the exciton density population,^[^ ^15^ ^]^ necessitating the development of the CMA materials with shorter excited‐state lifetime and higher reversed intersystem crossing rate (rISC) over 10^6^ s^−1^ (Scheme 1).

Fermi's Golden Rule describes the rate of RISC between singlet and triplet states and is shown in Scheme 1.^[^ ^16^, ^17^, ^18^ ^]^ According to Equation 1 the rISC rate can be accelerated by minimising the energy gap (ΔE_ST_) via reducing the density overlap integral between the frontier orbitals of TADF emitter, and achieved by twisted D‐A molecular design. However, the ΔE_ST_ relies on energy of the singlet and triplet states having the same charge transfer (CT) character, therefore, forbidden according to El‐Sayed's rule,^[^ ^19^ ^]^ having very poor H_SOC_ values (<^1^CT**|**H_SOC_ **|** ^3^CT> ≈0). The organometallic CMA materials overcome this limitation thanks to a heavy coinage metal atom bridging π‐donor (amide ligand) and π‐acceptor (carbene ligand) ligands, enabling efficient ISC in a few picoseconds^[^ ^6^, ^20^ ^]^ and luminescence radiative rates exceeding 2 × 10^6^ s^−1^ (Scheme 1, BG‐CMA).^[^ ^13^ ^]^ We previously established design strategies focused on maximising the energy gap between the charge‐transfer (CT) and locally‐excited (LE) triplet states in CMA complexes.^[^ ^13^, ^21^ ^]^ By employing a rigid benzoguanidine amide donor ligand, this approach led to complexes with ΔE_(CT–3LE)_ of over 0.5 eV resulting in record sub‐microsecond TADF lifetimes as low as 240 ns with unity quantum yields (Scheme 1).^[^ ^13^ ^]^ The champion, BG‐CMA, achieved deep‐blue electroluminescence with an LT_50_ of ≈1 h at 100 cd m^−2^, a record for blue‐emitting CMA complex. However, even these advanced CMA systems retained limited operational stability and appreciable EQE roll‐off at practical brightness over 20% necessitating further research to improve the CMA material.

In this work, we build on the success of the BG‐CMA materials and enlarge the CMA materials portfolio with *π*‐extended and rigid amide ligands, i.e., benzofuroindole (BFI) and benzothioindole (BTI), coordinated with (BiC)Au(I)‐moiety and display two new emitters: BiCAuBFI and BiCAuBTI (Scheme 1). We focus on CMA complexes with fused five‐ and six‐membered, BFI and BTI amides, aiming to explore the relative importance of the embedded chalcogen atom (X═O and S) that increases HSOC value in molecular design of the amide ligand and its effect on photoluminescent properties and OLED device performance. We find that title complexes maintain large ΔE_(CT–3LE)_ with fast sub‐microsecond and unity PLQY TADF emission. In particular, sulfur‐based BiCAuBTI achieves external quantum efficiencies exceeding 20% in both doped and neat sky‐blue OLEDs with negligible EQE roll‐off at high brightness even for a neat OLED device. These findings suggest that bimolecular degradation involving emitter–emitter interactions has been effectively suppressed.

## 2 Results and Discussion

**Figure 1:**
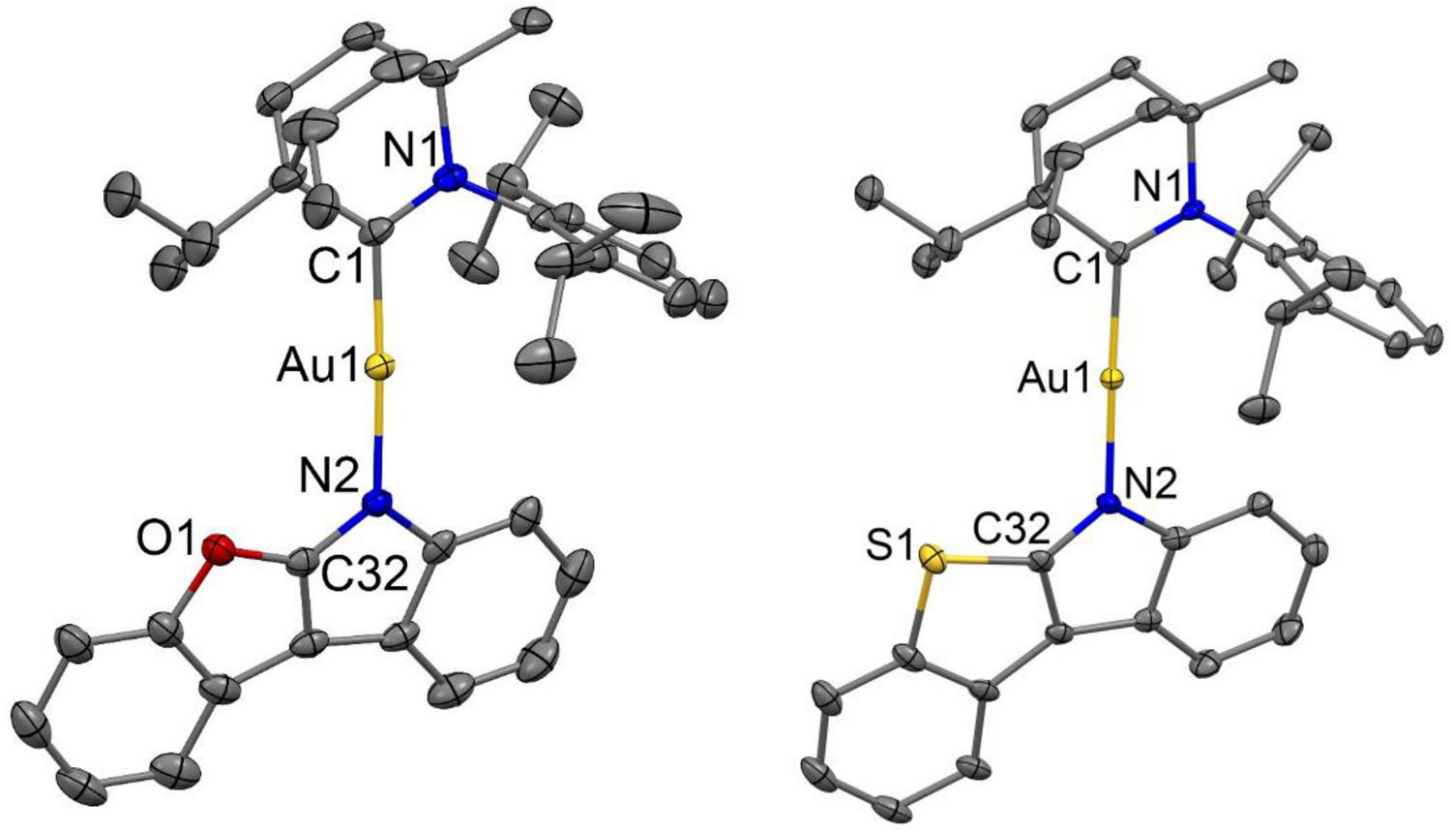
The crystal structures of the complex BiCAuBFI (left) and BiCAuBFI (right). Ellipsoids are shown at 50% probability. Hydrogen atoms and the CH_2_Cl_2_ solvate molecule are omitted for clarity.

Carbene–Metal–Amide (CMA) complexes BiCAuBFI and BiCAuBTI (Scheme 1 and **Figure** **1**) were prepared by reacting the halide complex (BiC)AuCl with either 6*H*‐benzofuro[2,3‐*b*]indole or 6*H*‐benzo[4,5]thieno[2,3‐*b*]indole, respectively in the presence of potassium *tert*‐butoxide in tetrahydrofuran according to our previously reported protocol.^[^ ^13^ ^]^ All complexes have been fully characterized by ^1^H and ^13^C{^1^H} NMR spectroscopy (Figures S1–S4, Supporting Information), high‐resolution mass spectrometry (HRMS), and X‐ray crystallography. The complexes are soluble in polar aprotic solvents such as dichloromethane and tetrahydrofuran and are sparingly soluble in non‐polar solvents such as hexane. The complexes are air‐stable for several months. Thermogravimetric analysis (TGA, 5% weight loss, see **Table** **1**; Figure S5, Supporting Information) indicates high thermal stability for the complexes with almost identical decomposition temperatures of 336 °C for BiCAuBFI and 337 °C for BiCAuBTI. These values show a significant improvement over recently reported BiCAuBG, which shows a decomposition temperature of 312 °C. Glass transition temperatures (*T_g_*) were estimated by differential scanning calorimetry (DSC, Figure S5, Supporting Information) showing a marked variation between *T_g_* for BiCAuBFI complex at 262 and 301 °C for complex BiCAuBTI. The *T_g_* values are up to 100 °C higher compared to our earlier reported CMA dendrimer,^[^ ^22^ ^]^ which can be explained by the high molecular mass and rigidity of the *π*‐extended amido ligand compared to the carbazole dendrimer donor.^[^ ^22^ ^]^

**Table 1:** Selected bond lengths (Å) and angles (°) for copper and gold complexes with thermal decomposition to 95% weight (T_90_) values, and calculated bond dissociation energies (BDE).

CMA	Au─C1, [Å]	Au─N2, [Å]	C1─Au─N2, [°]	Torsion angle, C1‐N1···N2‐C32 (α, °)	T_95_, [°C]	BDE, eV	
						Au─C1	Au─N2
**BG**	1.984(13)	2.025(6)	175.3(4)	17.0(2)	312	4.16	3.83
**BFI**	1.976(7)	2.024(6)	177.5(3)	6.9(3)	336	4.27	3.60
**BTI**	1.983(6)	2.017(4)	177.6(2)	10.5(3)	337	4.26	3.72

All gold complexes were crystallized by slow diffusion of hexane into a dichloromethane solution. The Au─C and Au─N bond lengths for the title molecules are negligibly different from the analogous structure of the BiCAuBG complex (Table 1) with differences lying within the error of the experiment. Both compounds BiCAuBFI and BiCAuBTI are isostructural and isomorphous with molecules possessing the C1─Au─N2 bond angle closer to the ideal value of 180° with a torsion angle between carbene and amide ligand being 7° smaller than in BiCAuBG complex (Table 1). This indicates that the common two‐coordinate linear geometry with a co‐planar arrangement of the ligands around the gold(I) atom is more pronounced for the title complexes. Both BiCAuBFI and BiCAuBTI crystals contain two independent molecules in their unit cell. Molecule A experiences a static disorder of the isopropyl moiety for the BiC‐carbene ligand while having a three‐dimensional network of weak C─H(carbene)∙∙∙*π*(amide) to form zigzag chains along the crystallographic *c‐*axis (Figure S6a, Supporting Information). Molecule B lacks disorder and possesses weak C─H(carbene)∙∙∙*π*(amide) and C─H(carbene)∙∙∙N2(amide) interactions between two antiparallel oriented molecules B, which is a repeating unit in a two‐dimensional network of molecules in the crystallographic *bc*‐plane. This is a major structural difference between A and B independent molecules for isostructural complexes BiCAuBFI and BiCAuBTI.

### 2.1 Electrochemistry

Electrochemical properties of the complexes were studied in THF solutions with [n‐Bu_4_N]PF_6_ as the supporting electrolyte with data collected in Table S1 (Supporting Information) (Figures S7 and S8, Supporting Information). Both complexes show a quasi‐reversible reduction process at −2.80 ± 0.01 V. This is consistent with BiCAuBG (−2.78 V), indicating the reduction process is centred on the BiC‐carbene ligand. This assignment is supported by the theoretical calculations, where the lowest occupied molecular orbital (LUMO, Table S4, Supporting Information) is largely localized over the BiC‐carbene ligand. The energy of the LUMO is estimated from the reduction profile onset as −2.68 eV for both complexes. The oxidation process for both BiCAuBFI and BiCAuBTI complexes is irreversible and centred on the *π*‐extended amide ligand. The oxidation potential of BiCAuBTI is higher by 0.08 V at +0.18 V, compared with BiCAuBFI at +0.10 V. This leads to minor differences for the highest occupied molecular orbital (HOMO) energy level at −5.40 eV (BiCAuBFI) and −5.45 eV for BiCAuBTI, respectively. The HOMO energies are somewhat destabilized compared to BiCAuBG (−5.53 eV), while the oxidation potential is anodically shifted up to +0.28 V compared to BiCAuBTI. This indicates a greater electron‐donor character of the BFI and BTI amide ligands thanks to conjugation with the electron‐rich oxygen or sulfur atoms compared to only nitrogen‐based BG ligand. Such a fact suggests a 0.2 eV smaller HOMO‐LUMO energy gap for the title complexes with a red‐shifted luminescence compared to BiCAuBG.

### 2.2 Photophysical Properties and Theoretical Calculations

**Figure 2:**
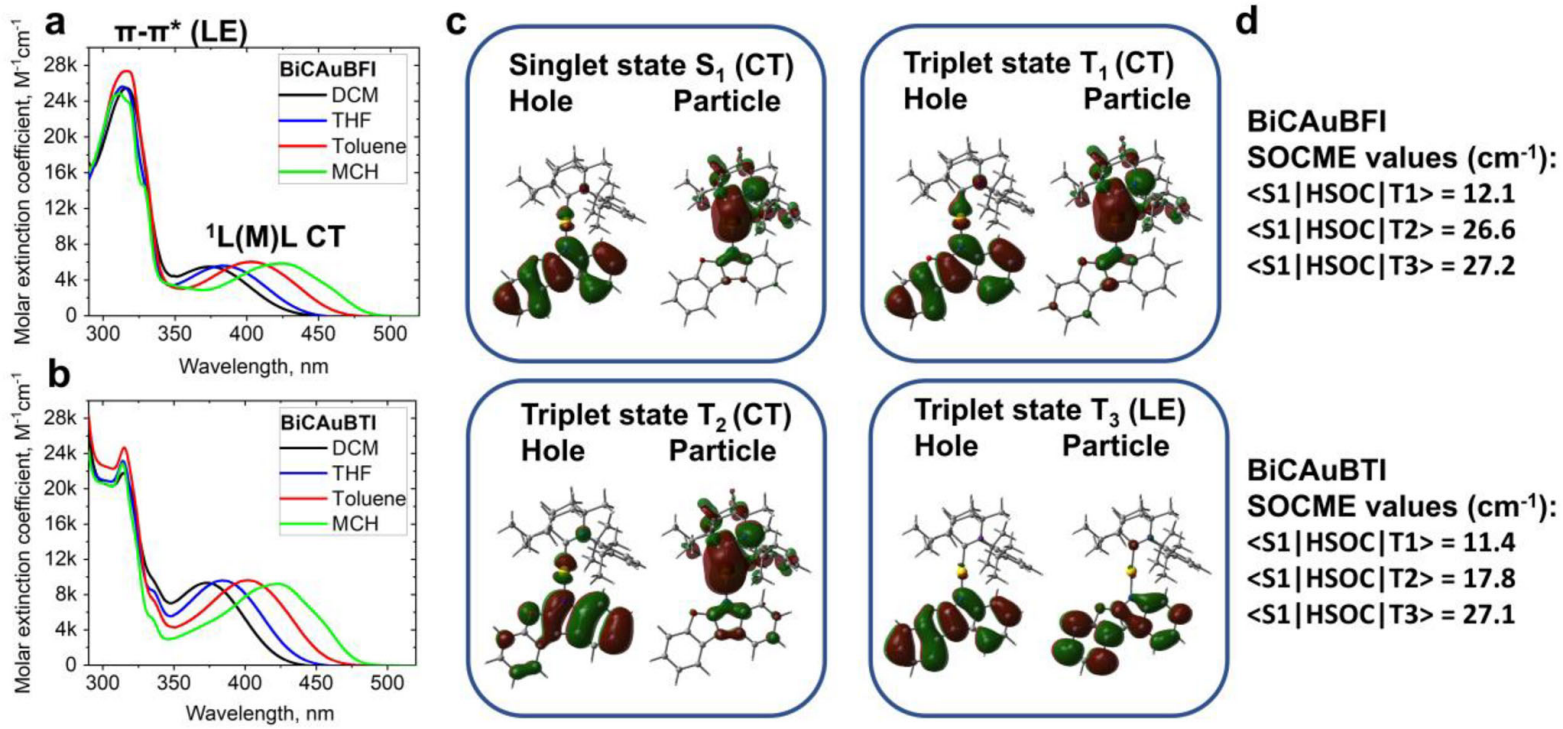
UV–vis spectra of BiCAuBFI a) and BiCAuBTI b) in various solvents at 295 K. c) Calculated the highest occupied natural transition orbitals (HONTOs) and lowest unoccupied natural transition orbitals (LUNTOs) for vertical singlet and triplet excited states for BiCAuBFI. d) spin–orbit coupling matrix elements (SOCME, cm^−1^) for the title complexes.

UV–vis absorption spectra were measured in various solvents with increasing polarity from dichloromethane, tetrahydrofuran, toluene, and methylcyclohexane (MCH), and are shown in (**Figure** **2**; Table S2, Supporting Information). Both complexes in the short wavelength region of the UV–vis spectra show an intense absorption at ≈314 nm, which is ascribed to the overlapping *π*–*π* ^*^ intraligand (IL) transitions of the BFI/BTI (amide) and BiC‐carbene ligands (Figure 2) with a similar extinction coefficient ≈24.8·10^4^ m ^−1^ cm^−1^. A low‐energy broad absorption band was measured at 424 nm with extinction coefficients for BiCAuBTI (ε = 9.2·10^3^ m ^−1^ cm^−1^) which is nearly two times higher than that for BiCAuBFI (ε = 5.9·10^3^ m ^−1^ cm^−1^). This band was ascribed to a singlet ligand‐to‐ligand charge transfer 1L(M)LCT band from the extended amide ligand (BFI or BTI) to BiC‐carbene ligand. Strong solvatochromism of the L(M)LCT band with a hypsochromic shift of ≈50 nm is measured on increasing the solvent polarity from methylcyclohexane to dichloromethane for both complexes (Figure 2), which is characteristic for the CMA materials. The assignment of the ^1^L(M)LCT band was further supported with theoretical DFT calculations revealing that S_1_ state has 98% HOMO→LUMO character (Figure 2; Tables S7 and S8, Supporting Information) with only a minor contribution (11–12%, Table S4, Supporting Information) of the gold orbitals. Theoretical calculations result in higher oscillator strength coefficients (f or probability to emit a photon) for S_0_→S_1_ transition in the case of complex BiCAuBTI (f = 0.1711) compared with BiCAuBFI (f = 0.1515) thus supporting experimentally measured higher extinction coefficients for the former complex. The f values are directly proportional to the overlap integral between HOMO and LUMO orbitals (S_H/L_) and parallel well with the larger orbital overlap integral for gold complex BiCAuBTI (S_H/L_ = 0.31) as compared to BiCAuBFI (SH/L = 0.29). This theoretical result suggests that the BiCAuBFI complex should have a faster radiative rates and a smaller energy gap (ΔE_ST_ values) between the lowest excited singlet and triplet states, which is achieved by minimizing the S_H/L_ values in TADF materials.

**Figure 3:**
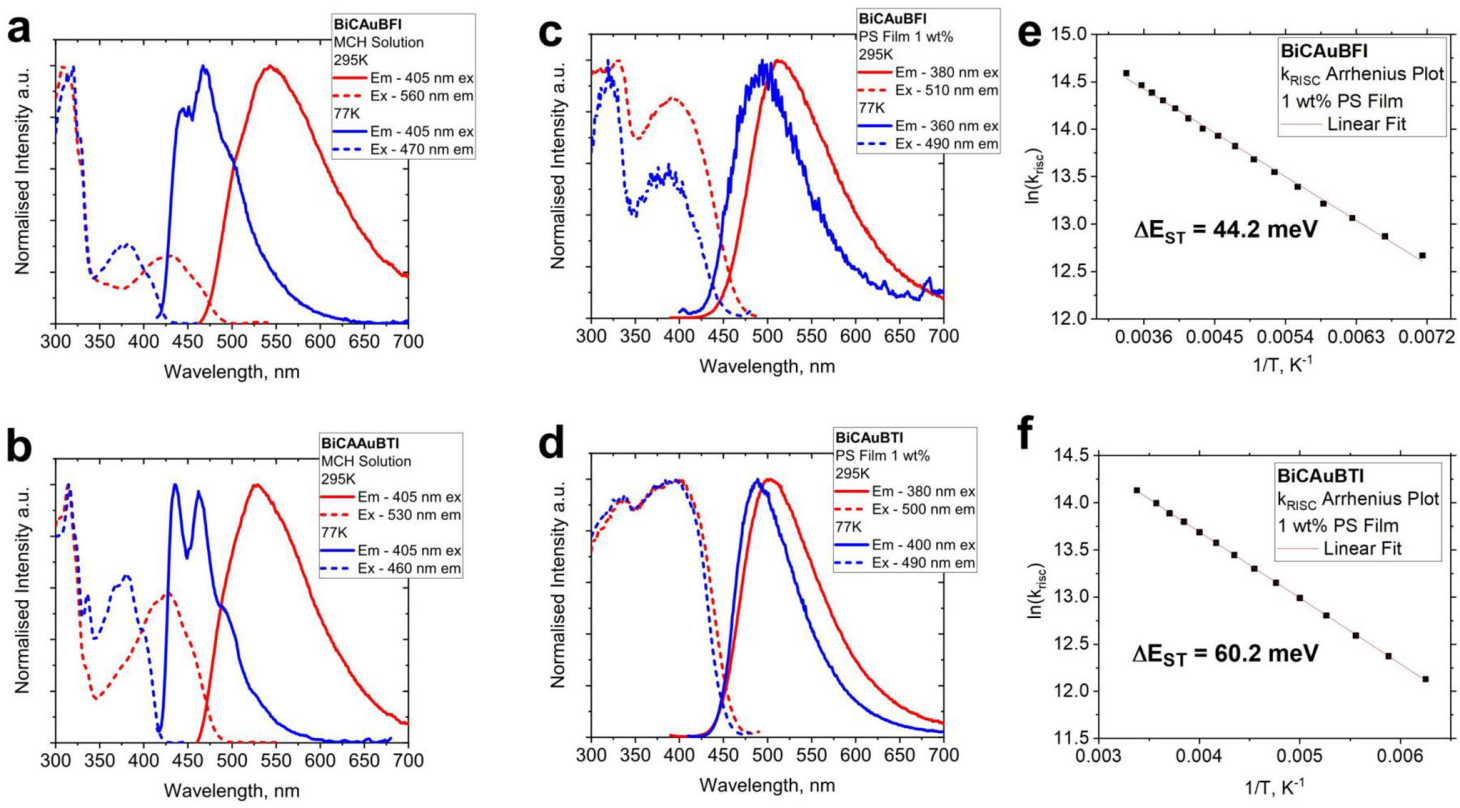
Excitation and emission spectra at 298 (red profile) and 77 K (blue profile) for BiCAuBFI (top) and BiCAuBTI (bottom) in methylcyclohexane solution and glass (left a and b), in 1 wt.% polystyrene matrix (middle c and d), and Arrhenius plots with estimated thermal activation energy barrier (right e and f).

To identify the CMA material with the best photophysical performance, we measured the luminescence properties of 1 wt.% gold complexes in polystyrene (PS) matrix, toluene, and methycyclohexane at 295 and 77 K. Data collected are summarized in **Table** **2** and displayed in (**Figure** **3**; Figures S11 and S12, Supporting Information). Both gold complexes in the PS matrix exhibit a featureless green CT luminescence at 511 nm for BiCAuBFI and 501 nm for BiCAuBTI (Figure 3). The emission peak for the title complexes is close to that of the benchmark CMA1 material (≈505 nm) and up to 50 nm red‐shifted compared to BiCAuBG (≈466 nm).^[^ ^6^, ^22^ ^]^ This result supports the 0.2 eV smaller HOMO‐LUMO energy gap (vide supra the electrochemistry data) for the title complexes while indicating that the electron donor strength of the BFI and BTI‐amide ligands is similar to that of the carbazolide ligand in benchmark CMA1 material. All gold complexes in PS matrix show a unity photoluminescence quantum yield (PLQY) whereas the excited state is nearly two times shorter for BiCAuBFI (473 ns) compared to BiCAuBTI (715 ns). Therefore, complex BiCAuBFI shows the champion radiative rate of 2.1 × 10^6^ s^−1^ (two‐times faster than the benchmark green CMA1 emitter) and supports the theoretical prediction for complex BiCAuBFI to have the highest radiative rates, as evidenced by the lower S_H/L_ values (vide supra).

**Table 2:** Photoluminescent properties of BiCAuBFI and BiCAuBTI in 1% by weight polystyrene matrix (PS), toluene solution, and methylcyclohexane solution at 295 and 77 K.

	λ_em_ [nm]	Τ [ns]	Φ [%]^a)^	k_r_ [10^6^ s^−1^]^b)^	k_nr_ [10^6^ s^−1^]^c)^	CT/LE/Δ*E*(_CT‐3LE_) (eV)^d)^	λ^e)^_m_ [nm, 77 K]	Τ [µs, 77 K]
1 wt.% PS matrix								
**BiCAuBFI**	511	473	100	2.11	–	2.75/2.83/0.08^e^	492	23.4 (16%) 65.3 (84%)
**BiCAuBTI**	501	715	100	1.40	–	2.78/2.81/0.03^e^	488	48.2 (26%) 87.7 (74%)
Toluene solution								
**BiCAuBFI**	582	88.7	39	4.40	6.88	2.54/2.87/0.33	482	63.2
**BiCAuBTI**	558	340	71	2.09	0.85	2.61/2.91/0.3	469	69.9
Methylcyclohexane solution								
**BiCAuBFI**	543	367	58	1.58	1.14	2.62/2.93/0.31	467	63.9
**BiCAuBTI**	529	740	85	1.15	0.20	2.65/2.94/0.29	435	62.4

^a)^ Quantum yields determined using an integrating sphere.

^b)^ radiative rate constant *k*_r_ = *Φ/ τ*

^c)^ Nonradiative constant *k*_nr_ = (1 – *Φ*)*/ τ*. In case of two‐component lifetime τ an average was used: *τ_av_ =(B_1_/(B_1_ +B_2_*))*τ_1_ + (B_2_/(B_1_ +B_2_*))*τ_2_*, where B1 and B2 are the relative amplitudes for *τ_1_* and *τ_2_*, respectively

^d)^ CT/LE energies based on the onset values of the emission spectra blue edges at 295 and 77 K, respectively

^e)^ CT energies based on the onset values of the emission spectra blue edges at 77 K

The luminescent properties for both gold complexes were measured as a function of temperature to verify the TADF nature of the luminescence. Upon cooling down to 77 K, both complexes maintain the emission profiles with a CT‐character while showing a blue‐shift by up to 21 nm (Figure 3). Thanks to near‐unity PLQY values, we can ignore the nonradiative processes and fit directly the temperature‐dependent PL decay curves to the Arrhenius equation (Figure 3; Figure S13, Supporting Information) of ln(*k*_RISC_) vs 1/T, which allows us to estimate the reverse intersystem crossing (rISC) activation energy (Δ*E*_a_) equal to 44.2 meV for BiCAuBFI, which is nearly two times smaller compared to 60.2 meV for BiCAuBTI. Note that the benchmark CMA1 material possesses a larger ΔE_a_ value of 69 meV;^[^ ^23^ ^]^ therefore, both complexes outperform CMA1 and are highly promising for applications in OLEDs.

We theoretically calculated the spin–orbit coupling matrix elements (SOCME, cm^−1^ Figure 2; Table S9, Supporting Information) to rationalize the 20 meV lower Δ*E_a_* estimated for BiCAuBFI compared to BiCAuBTI, despite the latter containing the slightly heavier chalcogen (oxygen vs. sulfur). Consistently higher SOCME values were calculated for BiCAuBFI between states of similar CT character, 〈S_1_|*Ĥ*_SOC_|T_1_〉 and 〈S_1_|*Ĥ*_SOC_|T_2_〉 compared to BiCAuBTI (Figure 2), indicating a more efficient coupling for 〈S_1_|*Ĥ*_SOC_|T_2_〉. We explain this with a more diffuse nature of sulfur orbitals compared to oxygen, which results in significantly lower electron density overlap with the neighbouring atoms. In contrast, the more compact oxygen orbitals maintain better overlap, allowing for stronger wavefunction mixing in the O‐based complex BiCAuBFI. This enhanced mixing propagates the heavy gold atom spin–orbit coupling effect throughout the entire CMA system more efficiently. This situation may override the direct heavy‐atom effect that would be expected from the chalcogen substitution alone (O→S). Both BiCAuBFI and BiCAuBTI complexes exhibit a similar SOCME value of ≈27 cm^−1^ for 〈S_1_|*Ĥ*_SOC_|T_3_〉, which is likely due to the different nature of the coupled states (CT for S_1_ and ^3^LE for T_3_). However, our previous works^[^ ^20^, ^21^ ^]^ revealed that coupling between ^1^CT and ^3^LE states is generally unfavourable for CMA materials. Therefore, the calculated higher SOCME values between the first CT singlet and second triplet states in BiCAuBFI corroborate well with the lower TADF activation barrier compared to BiCAuBTI, which is further reflected in OLED device performance.

Small ΔE_a_ is important but not a sufficient criterion to realize efficient CMA TADF materials with fast radiative rates. Recently, we demonstrated for a range of Aza‐CMA and BiCAuBG materials, of which the energy gap between CT and locally excited 3LE(amide) states, ΔE(CT–3LE), is a second key parameter for realizing fast radiative rates exceeding 10^6^ s^−1^. The larger the ΔE(CT–3LE) the faster the radiative rates for the CMA emitters become. Therefore, we measured the energy of ^1^CT and ^3^LE states (Table 2) for all gold complexes from high‐energy onset at 298 K and frozen methycyclohexane glass at 77 K. Both complexes show high CT energy at 2.62 eV for BiCAuBFI and 2.65 eV for BiCAuBTI at room temperature as a broad CT profile. On cooling to 77 K, both complexes show broad and featureless ^3^CT profile in toluene (Figures S11 and S12, Supporting Information) whereas, in methycylcohexane glass, the emission profile is vibronically resolved, indicating the dominant contribution of the locally excited triplet state ^3^LE(amide) with excited state lifetime up to 100 times longer than the ^1^CT lifetime at 298 K (Table 2). The ^3^LE state energy is ≈2.93 eV for both complexes, which is ≈0.3 eV higher than the CT state and supports a superior radiative rate exceeding 2·10^6^ s^−1^ compared to benchmark material CMA1. TD‐DFT calculations were made to reveal the origin of the orbitals responsible for the CT and ^3^LE states for both complexes (Figure 2; Tables S7 and S8, Supporting Information).

The majority of the CMA materials, including the benchmark material CMA1, possess the first triplet T_1_ to have a CT character while the second triplet T_2_ state as ^3^LE(amide). Unlike all other CMA materials, title complexes BiCAuBFI and BiCAuBTI exhibit the first two triplet states (T_1_ and T_2_) to have a CT character originating from HOMO→LUMO and (HOMO–1)→LUMO transition, respectively. It's only the third T_3_ triplet corresponds to the ^3^LE state, which originates from the HOMO→(LUMO+3) or HOMO→(LUMO+4) transition and is located on the strained BFI/BTI‐amido ligands, respectively. Overall, our photoluminescence study and theoretical investigation indicate a promising applied potential as OLED materials for both complexes.

### 2.3 OLED Device Fabrication

**Figure 4:**
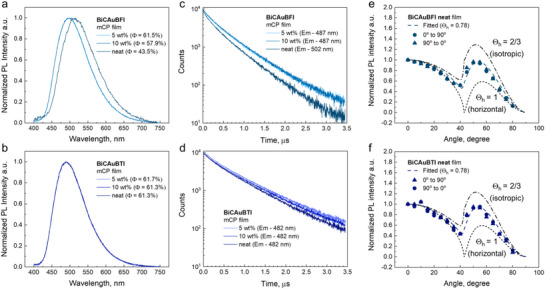
Photoluminescent (PL) properties of thin‐films of complexes BiCAuBFI and BiCAuBTI doped in 1,3‐bis(N‐carbazolyl)benzene (mCP) host at wt.% of 5% and 10% as well as those of their neat films at room temperature. a,b) PL spectra of the thin‐films, c,d) Time‐resolved PL decay of the thin‐films, e,f) Angle‐dependent PL intensity and molecular horizontal dipole orientation (Θ_*h*_) of the neat films.

To test the performance of the proposed CMA emitters, we fabricated thin films and OLEDs by thermal vapour deposition under high vacuum (10^−7^ Torr). To fabricate efficient CMA OLEDs, the photophysical properties were first measured for thin films doped with 5 and 10 wt.% of complexes BiCAuBFI and BiCAuBTI, doped in the host 1,3‐bis(N‐carbazolyl)benzene (mCP), and in a neat film. Consistent with the photophysical properties in solution, BiCAuBTI thin films have a shorter PL emission peak wavelength and longer exciton decay lifetime than BiCAuBFI thin films. It is noted that the photophysical properties of BiCAuBFI change as the concentration increases (5 wt.% → 10 wt.% → neat), whereas those of BiCAuBTI remain almost unchanged even with the increased concentration (**Figure** **4**; Table S10, Supporting Information). The peak wavelength of PL emission (*λ*_em,PL_) for the BiCAuBFI films red‐shifts by ≈15 nm and PLQY (Φ_PL_) decreases rapidly with the concentration, but BiCAuBTI films turn out to maintain the *λ*_em,PL_ of 482 nm, and Φ_PL_of ≈61%. Molecular horizontal dipole orientation (Θ_*h*_) of the mCP: BiCAuBFI film increases slightly from 70% to 75% when the emitter doping concentration is increased from 5to 10 wt.%, while the Θ_*h*_ of the mCP: BiCAuBTI film remains at 72% (Figure S14, Supporting Information). Through these steady‐state photoluminescence studies of thin films, it can be seen that BiCAuBFI is prone to aggregation‐induced degradation in its luminescent properties, while it is not the case for complex BiCAuBTI.

**Figure 5:**
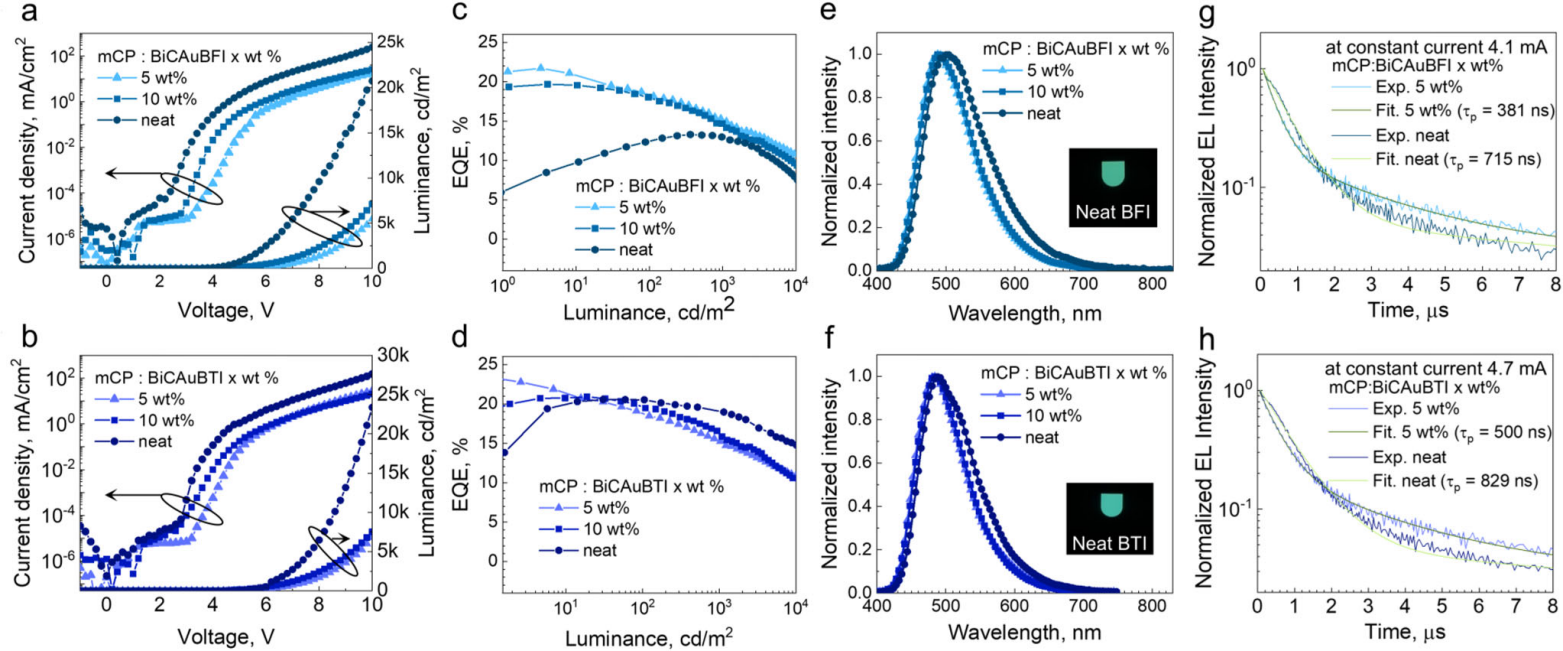
Electroluminescent characteristics of blue OLEDs based on CMA complexes: emission layers (EMLs) consist of doped thin‐films of complexes BiCAuBFI and BiCAuBTI in mCP host (5 or 10 wt.%) or respective neat films of BiCAuBFI and BiCAuBTI. a,b) Current density–voltage–luminance (*J*–*V*–*L*) characteristics. c,d) External quantum efficiency‐luminance (EQE‐*L*) characteristics. e,f) EL spectra and the photographs of the working blue OLEDs with the neat EMLs. g,h) Time‐resolved EL decay curves.

The electroluminescent (EL) properties of OLEDs adopting BiCAuBFI and BiCAuBTI as CMA TADF emitters were fabricated with transporting layers with appropriate HOMO and LUMO levels in the following device structures (Figure S15, Supporting Information): glass/indium‐tin‐oxide (ITO, 70 nm)/molybdenum oxide (MoO_3_, 4 nm)/1,1‐bis[(di‐4‐tolylamino)phenyl]cyclohexane (TAPC, 40 nm)/tris(4‐carbazoyl‐9‐ylphenyl)amine (TCTA, 10 nm)/1,3‐bis(*N*‐carbazolyl)benzene (mCP, 10 nm)/EML /Bis[2‐(diphenylphosphino)phenyl]ether oxide (DPEPO, 5 nm)/2,2',2″‐(1,3,5‐benzinetriyl)‐tris(1‐phenyl‐1‐*H*‐benzimidazole) (TPBi, 35 nm)/lithium fluoride (LiF, 1‐ nm)/aluminum (Al, 100 nm), in which EML refers to the emission layer based on BiCAuBFI or BiCAuBTI that are either doped into mCP (5 or 10 wt.%, 25 nm) or in a neat layer (15 nm). Their current density (*J*)–luminance (*L*)–voltage (*V*) characteristics are presented in **Figure** **5**a,b and the key parameters for EL performance are summarized in **Table** **3**. OLEDs based on BiCAuBFI and BiCAuBTI showed the highest maximum external quantum efficiency (EQE_max_) of 21.7% and 23.5%, respectively, when the doping concentration of the emitter was 5 wt.% (Figure 5c,d). Similar to what we observed for the PL of thin films, EQE_max_ of OLEDs based on the BiCAuBFI decreased rapidly from 21.7% to 13.3% as the doping concentration in the EML increases (5 wt.% → 10 wt.% → neat). The EL spectrum of the OLED based on the neat EML of BiCAuBFI exhibited, with respect to the OLED with the doped EML, the peak wavelength (*λ*_em,EL_) red‐shifted by ≈10 nm and the full width half‐maximum (FWHM) increased by 17 nm, mainly due to the broadening in the long wavelength side (Figure 5e,f). In the case of OLEDs based on BiCAuBTI, however, EQE_max_ remained to be more than 20% even for the devices with the neat emitter film, and *λ*_em,EL_ was red‐shifted only by ≈5 nm with respect to the OLEDs with the doped EML. Given with PLQY of 61%, this high EQE is attributed, in part, to the preferential horizontal dipole orientation (Θ_h_═0.78) of BiCAuBTI, which enhances light outcoupling efficiency in planar OLED structures^[^ ^24^, ^25^ ^]^ and helps maintain high performance even in the absence of a host matrix. Though not strong, slight Purcell enhancement in the radiative quantum yield within actual device structures is also expected to contribute to the observed high EQE, together with the high *Θ*_h_.^[^ ^26^ ^]^ The fact that OLEDs based on the neat film BiCAuBTI hold its efficiency well compared to those based on the doped EML is consistent with the PL properties of the films, in which BiCAuBTI films exhibited the aggregation‐resistant characteristics. It is also noteworthy that OLEDs with the neat EML exhibited a lower turn‐on voltage of 3.2 V compared to 4.4 V for the doped EML OLED (Figure 5a,b and Table 3). Such a lower turn‐on voltage and the broadened EL spectra in the long‐wavelength region, supported by the microsecond‐scale delayed emission observed in time‐resolved EL (Tr‐EL) (Figure 5g,h), indicate that the emission of the neat OLEDs may involve contributions from excimer‐related emission^[^ ^27^, ^28^, ^29^, ^30^ ^]^ and TTA‐induced fluorescence.^[^ ^31^ ^]^

**Table 3:** Electroluminescent characteristics of CMA complex blue OLEDs^a)^.

Emission layer	*V*_on_ ^b)^ [V]	*V*_op_ ^c)^ ^.^[V]	C.E.^d)^ [cd/A]	EQE^e)^ [%].	*λ*_em,EL_ ^f)^ [nm]	CIE (x,y)^g)^
mCP:**BiCAuBFI** 5 wt.%	4.4	7.6	53.1 / 38.1	21.7 / 15.3	490	(0.210, 0.379)
mCP:**BiCAuBFI** 10 wt.%	3.6	6.8	50.4 / 37.6	19.7 / 14.7	491	(0.210, 0.379)
Neat **BiCAuBFI**	3.2	5.4	35.7 / 33.8	13.3 / 12.6	501	(0.261, 0.449)
mCP:**BiCAuBTI** 5 wt.%	4.2	7.4	50.2 / 32.6	23.5 / 15.2	483	(0.193, 0.329)
mCP:**BiCAuBTI** 10 wt.%	3.6	7.4	46.2 / 35.0	20.9 / 15.8	483	(0.193, 0.329)
Neat **BiCAuBTI**	3.2	5.6	53.1 / 49.0	20.6 / 19.0	488	(0.220, 0.401)

^a)^ glass/ITO (70 nm)/MoO_3_ (4 nm)/TAPC (40 nm)/TCTA (10 nm)/mCP (10 nm)/mCP: emitter (5, 10 wt.%, 25 nm or neat, 15 nm)/DPEPO (5 nm)/TPBi (35 nm)/LiF (1 nm)/Al (100 nm).

^b)^ Applied voltage at a luminance of 1 cd m^−2^.

^c)^ Applied voltage at a luminance of 1000 cd m^−2^.

^d)^ Maximum current efficiency (CE): maximum CE at luminance (*L*) greater than 1 cd m^−2^, then the value at 1000 cd m^−2^.

^e)^ External quantum efficiency (EQE): maximum EQE at *L* >1 cd m^−2^, then the value at 1000 cd m^−2^.

^f)^ The peak wavelength of EL emission spectra.

^g)^ Color coordinates (CIE 1931) at maximum luminance.

Time‐resolved EL (Tr‐EL) data of OLEDs based on doped BiCAuBFI and BiCAuBTI EMLs exhibit typical TADF characteristics with the prompt decay constant (*τ*_prompt_) of 381 and 500 ns and the delayed decay constant (*τ*_delayed_) of 2.7 and 2.8 µs, respectively, estimated for 5 wt.% doped EMLs (Figure 5g,h). For the OLEDs with the neat EML, on the other hand, the decay lifetime (τ) is measured to be 715 ns for BiCAuBFI and 829 ns for BiCAuBTI. The fact that the decay constants are larger than *τ*_prompt_ and smaller than *τ*_delayed_ suggests bimolecular processes such as triplet–triplet annihilation (TTA) play a significant role in determining the emission properties of the OLEDs with the neat EML (Figure S16, Supporting Information).^[^ ^32^, ^33^, ^34^ ^]^ Furthermore, the voltage‐dependent time‐resolved EL decay curves (Figure S17, Supporting Information) exhibit a continuous reduction in slope across the entire decay region, consistent with TTA contributing not only to additional emission through triplet–triplet fusion but also to efficiency roll‐off via nonradiative annihilation at higher excitation densities in both doped and undoped OLEDs.^[^ ^31^ ^]^

**Figure 6:**
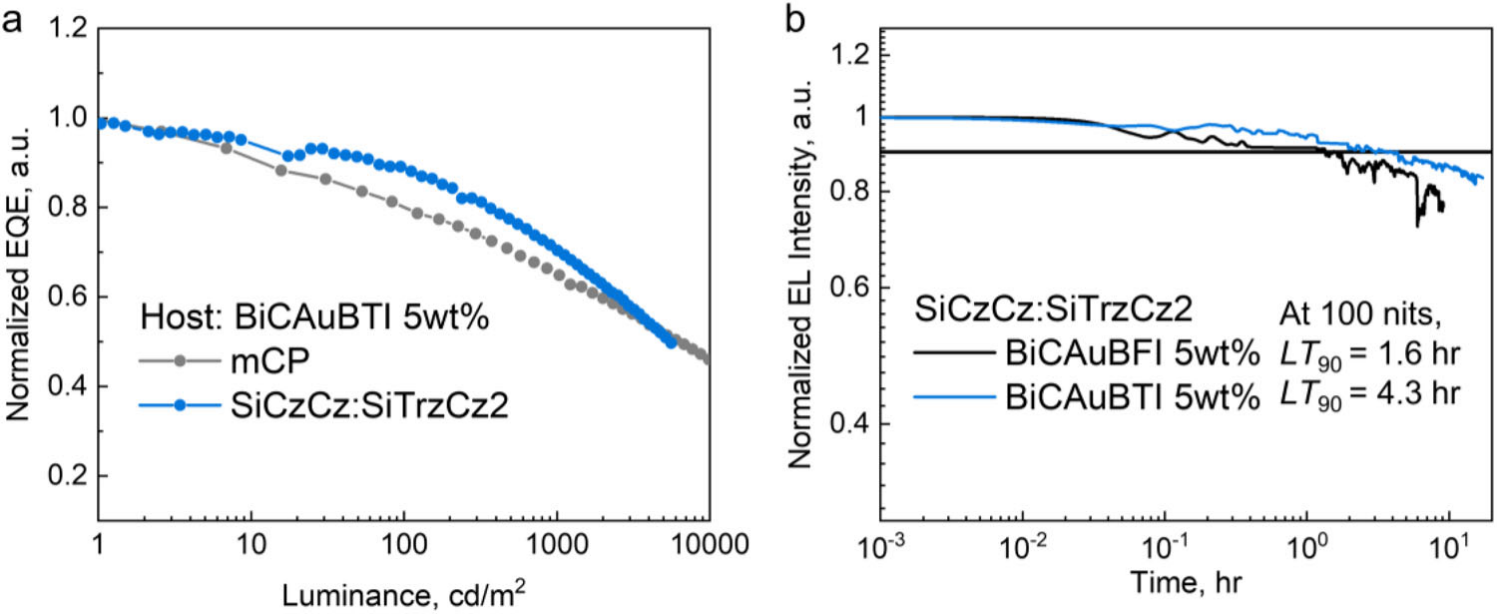
Roll‐off characteristics and operational stability of CMA‐complex‐based blue OLEDs (5wt.% in mCP). a) EQE‐roll off characteristics. b) Normalized EL intensity vs. operation time at the initial luminance of 100 nits.

To evaluate the operating stability of OLEDs based on BiCAuBFI and BiCAuBTI, the devices were fabricated by replacing the transport layer with materials containing fewer weak C─N bonds,^[^ ^35^, ^36^ ^]^ which have larger bond dissociation energies (BDE) than those of the C─N bonds in mCP, TAPC, TCTA, and DPEPO, as shown in Figure S17 (Supporting Information). The resultant device structures are as follows: glass/ ITO (70 nm)/ dipyrazino[2,3‐f:2',3'‐h]quinoxaline‐2,3,6,7,10,11‐hexacarbonitrile (HATCN, 10 nm)/ N‐([1,1′‐Biphenyl]‐4‐yl)‐9,9‐dimethyl‐N‐(4‐(9‐phenyl‐9H‐carbazol‐3‐yl)phenyl)‐9H‐fluoren‐2‐amine (BCFN, 60 nm) / 9‐(3‐(triphenylsilyl)phenyl)‐9H‐3,9′‐bicarbazole (SiCzCz, 5 nm)/ (9,9′‐(6‐(3‐(triphenylsilyl)phenyl)‐1,3,5‐triazine‐2,4‐diyl)bis(9H‐carbazole), SiTrzCz2) SiCzCz: SiTrzCz2:emitter 5 wt.% (6:4, 35 nm)/ 2‐Phenyl‐4,6‐bis(3‐(triphenylsilyl)phenyl)‐1,3,5‐triazine (mSiTrz, 5 nm)/ (lithium‐8‐hydroxyquinolinolate, Liq) mSiTrz:Liq (1:1, 31 nm)/ LiF (1 nm)/ Al (100 nm). As a result, the turn‐on voltage increased and the EQE decreased (Figure S18 and Table S11, Supporting Information), whereas the EQE roll‐off was improved in OLEDs using SiCzCz:SiTrzCz2 as an exciplex host when compared with OLEDs using mCP host. At 100 nits, *LT*_90_ of 1.6 and 4.3 hr were obtained for OLEDs with BiCAuBFI (5 wt.%) and BiCAuBTI (5 wt.%; **Figure** **6**), respectively, indicating that BiCAuBTI is a better candidate for emitter molecules in terms of both efficiency and operation stability. Overall, the OLED performance for sulfur‐based BiCAuBTI emitter appeared significantly improved compared to oxygen‐based BiCAuBFI, which is prone to the EQE roll‐off under all OLED tested architectures.

## 3 Conclusion

We have demonstrated that new gold(I)‐based CMA materials incorporating electron‐donating chalcogen substitution (oxygen or sulfur) in the *π*‐extended amide donor ligand yield bright sky‐blue TADF luminescence, red‐shifted by 30 nm compared to BG‐CMA (Scheme 1) materials that contain multiple electron‐withdrawing aza‐groups in the amide donor. We find that the combination of molecular rigidity and increased heavy‐atom spin–orbit coupling (HSOC) values within the donor ligand contributes to high radiative rates exceeding 10^6^ s^−1^. Electrochemical studies reveal a 0.2 eV smaller HOMO–LUMO energy gap for the new complexes relative to BG‐CMA, attributed to the electron‐rich oxygen or sulfur atoms in the amide donor ligand that destabilize the HOMO energy level.

Incorporation of the heavier sulfur atom in BiCAuBTI complex results in a slightly higher reversed intersystem crossing (RISC) activation energy of 60 meV, compared to 44 meV for the oxygen‐based CMA complex BiCAuBFI. It was found that higher SOCME values between CT singlet and triplet states in BiCAuBFI are likely associated with a lower TADF activation barrier compared to BiCAuBTI. However, steady‐state photoluminescence studies of thin films show that BiCAuBFI is prone to aggregation‐induced reduction in emissive efficiency in its luminescent properties, whereas this BiCAuBTI is not. This difference translates into improved efficiency and operational stability of the OLED device with BiCAuBTI, showing no aggregation phenomenon. OLED operating stability LT_90_ (time to reach 90% of initial luminance at 100 cd m^−2^) was measured to be 1.6 h for BiCAuBFI (5 wt.%) and 4.3 h for BiCAuBTI (5 wt.%), indicating that BiCAuBTI is the more promising candidate for the development of stable OLED devices.

This work demonstrates that the combination of sub‐microsecond TADF, resistance to aggregation, and tailored host selection provides a viable strategy to overcome longstanding limitations in the operational stability of CMA‐based OLEDs. Due to reduced aggregation‐induced quenching, the sulfur‐based BiCAuBTI emitter achieves over 20% external quantum efficiency (EQE) in a neat OLED device, with negligible EQE roll‐off at a high brightness of 1000 cd m^−2^. This finding not only enables a simplified OLED architecture based on the BiCAuBTI complex but also suggests a molecular design approach for the next‐generation CMA materials resistant to bimolecular quenching processes.

## Conflict of Interest

The authors declare no conflict of interest.

## Author Contributions

A.C.B. and J.J. contributed equally to this work. A.C.B performed the synthesis, steady‐state photoluminescence and electrochemistry studies. J. J. performed the steady‐state photoluminescence studies of thin‐films. J. J. also developed and characterized the OLED devices. D. L. and J. K. supported to characterize the OLED devices. A.S.R. performed X‐ray crystallography. N.L.P. and M.L. carried out theoretical calculations and analysis of the computational data. A.S.R., M.L., J. J., S.Y. planned the project and designed the experiments. A.C.B., A.S.R, J.J., S.Y. co‐wrote the manuscript. All authors contributed to the discussion of the results, analysis of the data and reviewed the manuscript.

## Supporting information



Supporting Information

## Data Availability

The data underlying this publication are available through the following web link.
