# Supplementary material for: Carbene–Metal–Amide Materials Design: Tailoring π‐Extended Amides for High‐Performance Organic Light‐Emitting Diodes
Source: Adv Sci (Weinh). 2025 Nov 14;13(6):e16582. doi: 10.1002/advs.202516582 (PMC12866764; doi:10.1002/advs.202516582)
Supplement: Supplementary file 1 — Supporting Information [file ADVS-13-e16582-s001.docx]

**Supporting Information for**

**Carbene-Metal-Amide Materials Design: Tailoring π-Extended Amides for High Performance Organic Light-Emitting Diodes**

Alexander C. Brannan,† Jeoungmin Ji,† Nguyen Le Phuoc, Donggyun Lee, Junho Kim, Mikko Linnolahti,* Seunghyup Yoo* and Alexander S. Romanov*

Table of Contents:

General considerations 2

Synthetic procedures 2

X-ray crystallography 8

Electrochemistry 10

Photophysical characterisation 12

Computational Details 18

OLED devices 24

References 29

**General Considerations**

All reactions were performed under a N_2_ atmosphere. Solvents were dried as required. **BiCAuCl**, 6*H*-benzofuro[2,3-*b*]indole and 6*H*-benzo[4,5]thieno[2,3-*b*]indole were prepared by following literature procedures.^[[1]](#endnote-1),^^[[2]](#endnote-2)^ ^1^H and ^13^C{^1^H} NMR spectra were recorded using a Bruker AVIII HD 500 MHz NMR spectrometer. ^1^H NMR spectra (500.19 MHz) and ^13^C{^1^H} (125.79 MHz) were referenced to acetone-d_6_ at δ 2.05 (^13^C, δ 29.84). Elemental analyses were performed by the Microanalysis Laboratory at the University of Manchester. Mass spectrometry data were obtained by the Mass Spectrometry Laboratory at the University of Manchester. Thermogravimetric analyse were performed by the Microanalysis Laboratory at the University of Manchester.


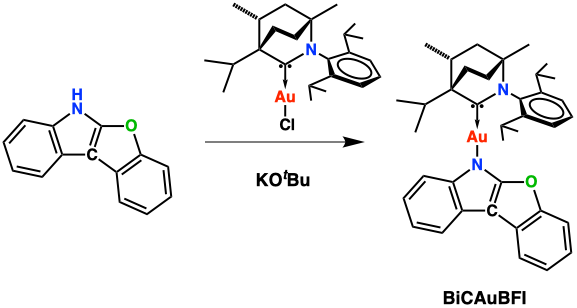


**Synthesis of BiCAuBFI**

Tetrahydrofuran (35 ml) was added to **BiCAACAuCl** (1.50 g, 2.62 mmol), potassium *tert*-butoxide (324 mg, 2.88 mmol) and 6*H*-benzofuro[2,3-*b*]indole (543 mg, 2.62 mmol) at −78 ºC and was allowed to slowly warm to room temperature and stir overnight. The reaction mixture dried under vacuum. The crude product was extracted with dichloromethane and filtered through Celite^®^. The crude product was purified by column chromatography (eluent 1:4 ethyl acetate:hexane) to give the pure product as a pale-yellow crystalline powder in 70% yield (1.37 g, 1.84 mmol). Single crystals suitable for X-ray diffraction were grown by layering a concentrated solution in DCM with pentane. ^1^H NMR (500 MHz, CD_2_Cl_2_) δ 7.65 – 7.60 (m, 2H, *p*-CH Dipp and BFI Ar-CH), 7.58 (d, *J* = 7.5 Hz, 1H, BFI Ar-CH), 7.47 (d, *J* = 7.8 Hz, 1H, *m*-CH Dipp), 7.38 (pseudo-t, *J* = 8.2 Hz, 2H, *m*-CH Dipp and BFI Ar-CH), 7.18 (t, *J* = 7.5 Hz, 1H, BFI Ar-CH), 7.01 (t, *J* = 7.7 Hz, 1H, BFI Ar-CH), 6.95 (t, *J* = 7.5 Hz, 1H, BFI Ar-CH), 6.84 (t, *J* = 7.6 Hz, 1H, BFI Ar-CH), 6.52 (d, *J* = 8.1 Hz, 1H, BFI Ar-CH), 3.34 – 3.25 (m, 1H, CH *i*Pr Carbene), 3.15 – 3.05 (m, 1H, CH *i*Pr Dipp), 2.72 – 2.63 (m, 1H, CH *i*Pr Dipp), 2.42 – 2.33 (m, 1H, CH(CH_3_) Carbene), 2.19 (dd, *J* = 13.7, 10.5 Hz, 1H, CH Carbene), 1.92 – 1.76 (m, 3H, CH Carbene), 1.74 – 1.62 (m, 5H, CH Carbene, overlapping), 1.41 (d, *J* = 6.7 Hz, 3H, CH_3_ *i*Pr Dipp), 1.38 (d, *J* = 6.9 Hz, 3H, CH_3_ *i*Pr Dipp), 1.35 (d, *J* = 6.9 Hz, 3H, CH_3_ *i*Pr Dipp), 1.30 (d, *J* = 6.9 Hz, 3H, CH_3_ *i*Pr Dipp), 1.18 (d, *J* = 6.9 Hz, 3H, CH_3_ *i*Pr Carbene), 1.14 (pseudo-d, *J* = 6.6 Hz, 6H, CH(CH_3_), overlapping). ^13^C NMR (126 MHz, CD_2_Cl_2_) δ 246.31 (C:), 166.61 (Ar-C(NCO) BFI), 156.55 (Ar-CO BFI) BFI), 146.51 (Ar-C=C(NO) BFI), 145.57 (*o*-CH Dipp), 144.74 (*o*-CH Dipp), 142.21 (*i*-CH Dipp), 130.00 (*p*-C Dipp), 126.76 (Ar-C BFI), 125.56 (*m*-C Dipp), 125.54 (*m*-C Dipp), 122.88 (Ar-C BFI), 122.81 (Ar-CH BFI), 119.25 (Ar-CH BFI), 118.31 (Ar-C BFI), 118.27 (Ar-CH BFI), 117.73 (Ar-CH BFI), 117.45 (Ar-CH BFI), 115.61 (Ar-CH BFI), 111.03 (Ar-CH BFI), 96.90 (Ar-CH BFI), 64.64 (C(CCN) Carbene), 55.69 (C(CCC) Carbene), 44.54 (CH Carbene), 34.80 (CH(CH_3_) Carbene), 33.01 (CH Carbene), 32.27 (CH *i*Pr Carbene), 29.63 (CH *i*Pr Dipp), 29.05 (CH *i*Pr Dipp), 25.32 (CH_3_ *i*Pr Dipp), 25.05 (CH_3_ *i*Pr Dipp), 24.20 (CH_3_ *i*Pr Carbene), 24.07 (CH_3_ *i*Pr Carbene), 23.45 (CH_3_ *i*Pr Dipp), 21.48 (CH Carbene), 20.57 (CH Carbene), 19.59 (CH(CH_3_)), 16.52 (CH_3_ *i*Pr Dipp). HRMS C_38_H_45_AuN_2_O theoretical [M+H]^+^ = 743.3270, HRMS (APCI): = 743.3258.

**Figure S1.** ^1^H NMR (500 MHz, CD_2_Cl_2_)


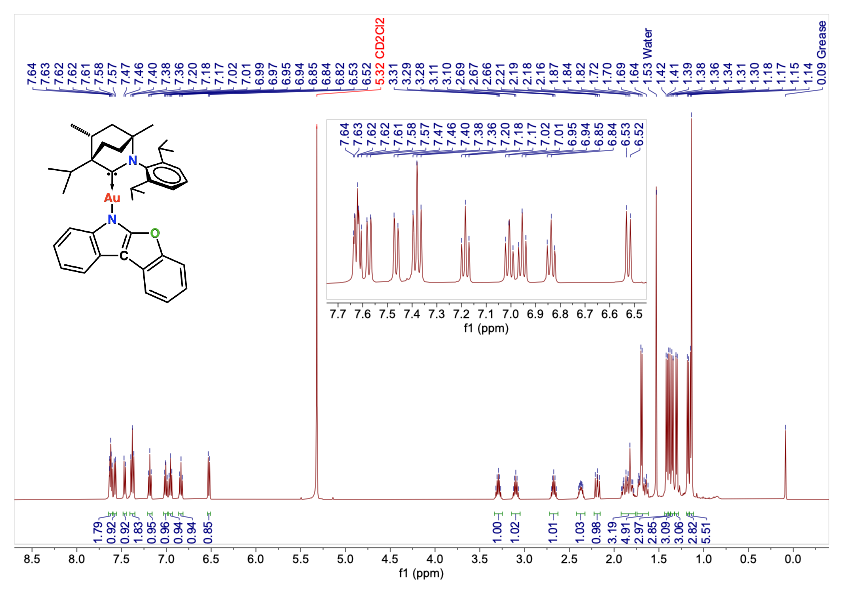


**Figure S2.** ^13^C{^1^H} NMR (126 MHz, CD_2_Cl_2_)


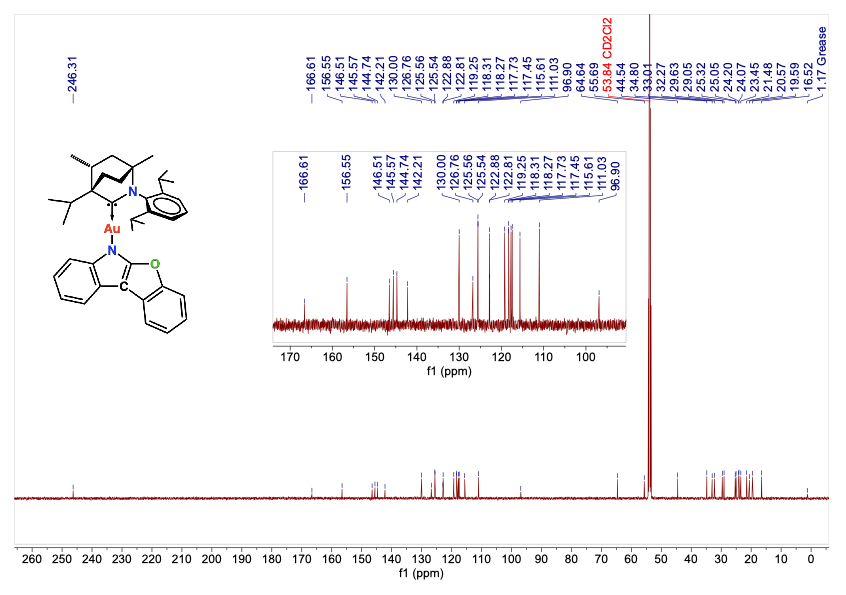


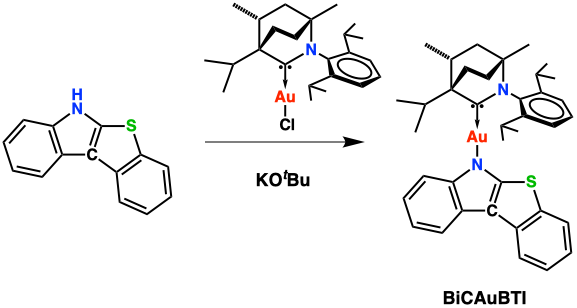


**Synthesis of BiCAuBTI**

Tetrahydrofuran (35 ml) was added to **BiCAACAuCl** (1.00 g, 1.75 mmol), potassium *tert*-butoxide (216 mg, 1.93 mmol) and 6*H*-benzo[4,5]thieno[2,3-*b*]indole (391 mg, 1.75 mmol) at −78 ºC and was allowed to slowly warm to room temperature and stir overnight. The reaction mixture dried under vacuum. The crude product was extracted with dichloromethane and filtered through Celite^®^. The crude product was purified by column chromatography (eluent 1:4 ethyl acetate:hexane) to give the pure product as a off-white crystalline powder in 56% yield (740 mg, 975 μmol). Single crystals suitable for X-ray diffraction were grown by layering a concentrated solution in DCM with pentane. ^1^H NMR (500 MHz, CD_2_Cl_2_) δ 7.88 (d, *J* = 7.8 Hz, 1H, BTI Ar-CH), 7.83 (d, *J* = 9.2 Hz, 1H, BTI Ar-CH), 7.71 – 7.62 (m, 2H, *p*-CH Dipp and BTI Ar-CH), 7.48 (d, *J* = 6.4 Hz, 1H, *m*-CH Dipp), 7.41 (d, *J* = 7.8 Hz, 1H, *m*-CH Dipp), 7.34 (t, *J* = 7.5 Hz, 1H, BTI Ar-CH), 7.08 (t, *J* = 7.6 Hz, 1H, BTI Ar-CH), 6.98 (t, *J* = 8.0 Hz, 1H, BTI Ar-CH), 6.93 (t, *J* = 7.6 Hz, 1H, BTI Ar-CH), 6.68 (d, *J* = 7.8 Hz, 1H, BTI Ar-CH), 3.35 – 3.24 (m, 1H, CH *i*Pr Carbene), 3.13 – 3.04 (m, 1H, CH *i*Pr Dipp), 2.71 – 2.61 (m, 1H, CH *i*Pr Dipp), 2.38 – 2.28 (m, 1H, CH(CH_3_) Carbene), 2.14 (dd, *J* = 13.8, 10.5 Hz, 1H, CH Carbene), 1.90 – 1.57 (m, 8H, CH Carbene, overlapping), 1.44 – 1.34 (m, 9H, CH_3_ *i*Pr Dipp), 1.30 (d, *J* = 6.9 Hz, 3H, CH_3_ *i*Pr Dipp), 1.18 (d, *J* = 6.7 Hz, 3H, CH_3_ *i*Pr Carbene), 1.14 – 1.07 (m, 6H, CH(CH_3_), overlapping). ^13^C NMR (126 MHz, CD_2_Cl_2_) δ 246.14 (C:), 153.49 61 (Ar-C(NCS) BTI), 151.15 (Ar-CS BFI) BTI), 145.65 (*o*-CH Dipp), 144.71 (*o*-CH Dipp), 142.27 (*i*-CH Dipp), 138.27 (Ar-C=C(NS) BTI), 134.66 (Ar-C BTI), 130.14 (*p*-C Dipp), 125.58 (*m*-C Dipp), 124.52 (Ar-CH BTI), 124.35 (Ar-C BTI), 123.57 (Ar-CH BTI), 120.49 (Ar-CH BTI), 119.51 (Ar-CH BTI), 119.23 (Ar-CH BTI), 117.81 (Ar-CH BTI), 117.69 (Ar-CH BTI), 116.94 (Ar-C BTI), 114.87 (Ar-CH BTI), 64.62 (C(CCN) Carbene), 55.69 (C(CCC) Carbene), 44.44 (CH Carbene), 34.75 (CH(CH_3_) Carbene), 32.96 (CH Carbene), 32.25 (CH *i*Pr Carbene), 29.62 (CH *i*Pr Dipp), 29.04 (CH *i*Pr Dipp), 25.45 (CH_3_ *i*Pr Dipp), 25.13 (CH_3_ *i*Pr Dipp), 24.14 (CH_3_ *i*Pr Carbene), 24.08 (CH_3_ *i*Pr Carbene), 23.39 (CH_3_ *i*Pr Dipp), 21.57 (CH Carbene), 20.52 (CH Carbene), 19.54 (CH(CH_3_)), 16.56 (CH_3_ *i*Pr Dipp). HRMS C_38_H_45_AuN_2_S theoretical [M+H]^+^ = 759.3042, HRMS (APCI): = 759.3029.

**Figure S3.** ^1^H NMR (500 MHz, CD_2_Cl_2_)


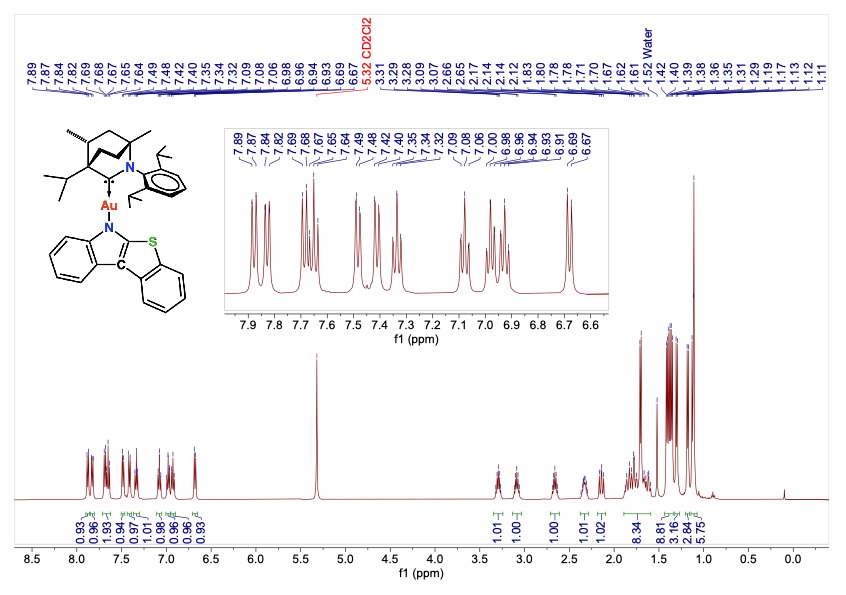


**Figure S4.** ^13^C{^1^H} NMR (126 MHz, CD_2_Cl_2_)


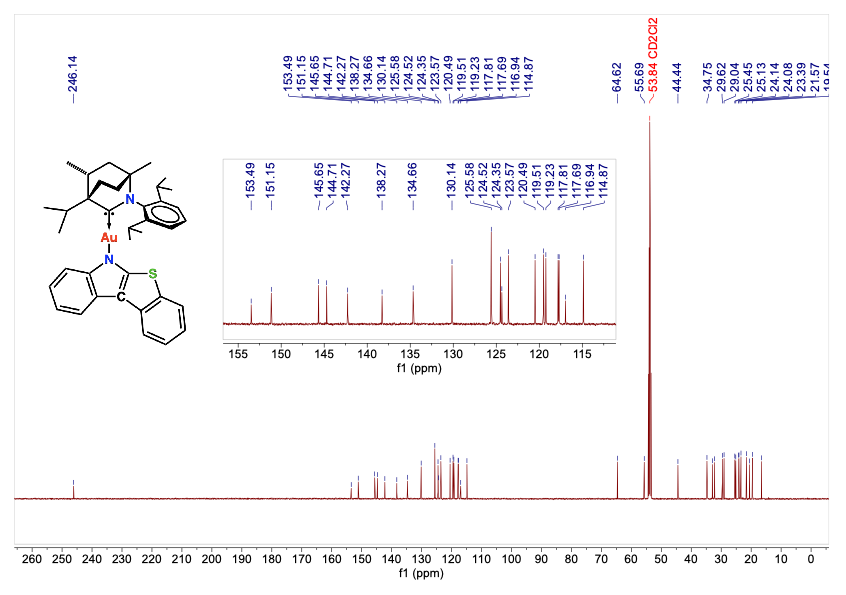


**Thermogravimetric Analysis & Differential Scanning Calorimetry.**

**
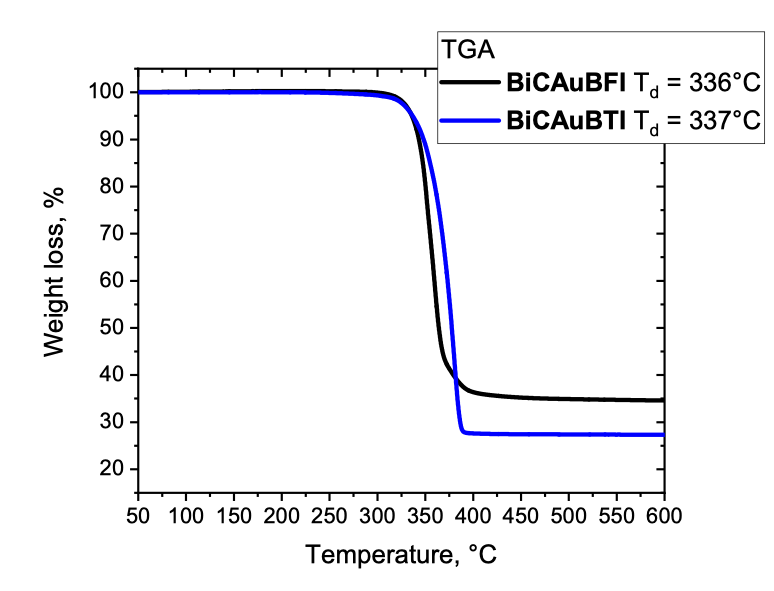

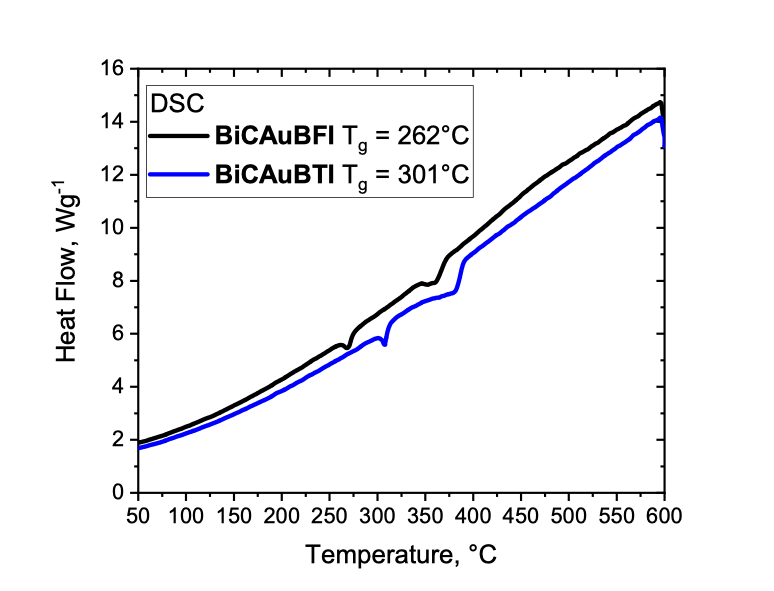
**

**Figure S5.** TGA (left) and DSC (right) curves for **BiCAuBFI** and **BiCAuBTI** complexes. Decomposition temperature (T_d_) indicates the temperature at 5% weight loss.

**X-ray Crystallography.**

Crystals suitable for X-ray diffraction study were obtained by layering the dichloromethane solution of gold complexes with pentane at room temperature. Crystals were mounted in oil on glass fiber and fixed on the diffractometer in a cold nitrogen stream. Data was collected using Rigaku Oxford Diffraction XtaLAB Synergy-S diffractometer at 100 K. Data were processed using the CrystAlisPro-CCD and –RED software.^[[3]](#endnote-3)^ The structure was solved by intrinsic phasing or direct method and refined by the full-matrix least-squares against F2 in an anisotropic (for non-hydrogen atoms) approximation. All hydrogen atom positions were refined in isotropic approximation in a “riding” model with the U_iso_(H) parameters equal to 1.2U_eq_(C_i_), for methyl groups equal to 1.5U_eq_(C_ii_), where U(C_i_) and U(C_ii_) are respectively the equivalent thermal parameters of the carbon atoms to which the corresponding H atoms are bonded. All calculations were performed using the SHELXTL software.^^[[4]](#endnote-4)^,^[[5]](#endnote-5)^^ OLEX2 software was used as graphical user interface.^^[[6]](#endnote-6)^^ To model disorder the library of the idealized fragments was used.^^[[7]](#endnote-7)^^

**(BiC)AuBFI**, CCDC number 2470099, C_38_H_44.5_AuN_2_O (*M*=742.22 g/mol): monoclinic, space group P2_1_/c (no. 14), *a* = 20.8125(6) Å, *b* = 20.7259(5) Å, *c* = 16.9018(5) Å, *β* = 113.846(3)°, *V*= 6668.3(3) Å^3^, *Z* = 8, *T* = 99.8(6) K, μ(Mo Kα) = 4.443 mm^-1^, *Dcalc* = 1.479 g/cm^3^, 55985 reflections measured (6.716° ≤ 2Θ ≤ 58.242°), 16041 unique (*R*_int_ = 0.0542, R_sigma_ = 0.0679) which were used in all calculations. The final *R*_1_ was 0.0548 (I > 2σ(I)) and *wR*_2_ was 0.1138 (all data).

**(BiC)AuBTI**, CCDC number 2470098, C_38_H_44.5_AuN_2_S (*M*=758.28 g/mol): monoclinic, space group P2_1_/c (no. 14), *a* = 20.7414(6) Å, *b* = 21.0358(5) Å, *c* = 16.9067(5) Å, *β* = 113.779(3)°, *V*= 6750.4(3) Å^3^, *Z* = 8, *T* = 99.9(2) K, μ(Mo Kα) = 4.448 mm^-1^, *Dcalc* = 1.492 g/cm^3^, 63226 reflections measured (6.554° ≤ 2Θ ≤ 58.444°), 16161 unique (*R*_int_ = 0.0682, R_sigma_ = 0.0651) which were used in all calculations. The final *R*_1_ was 0.0480 (I > 2σ(I)) and *wR*_2_ was 0.1123 (all data).

**a)**


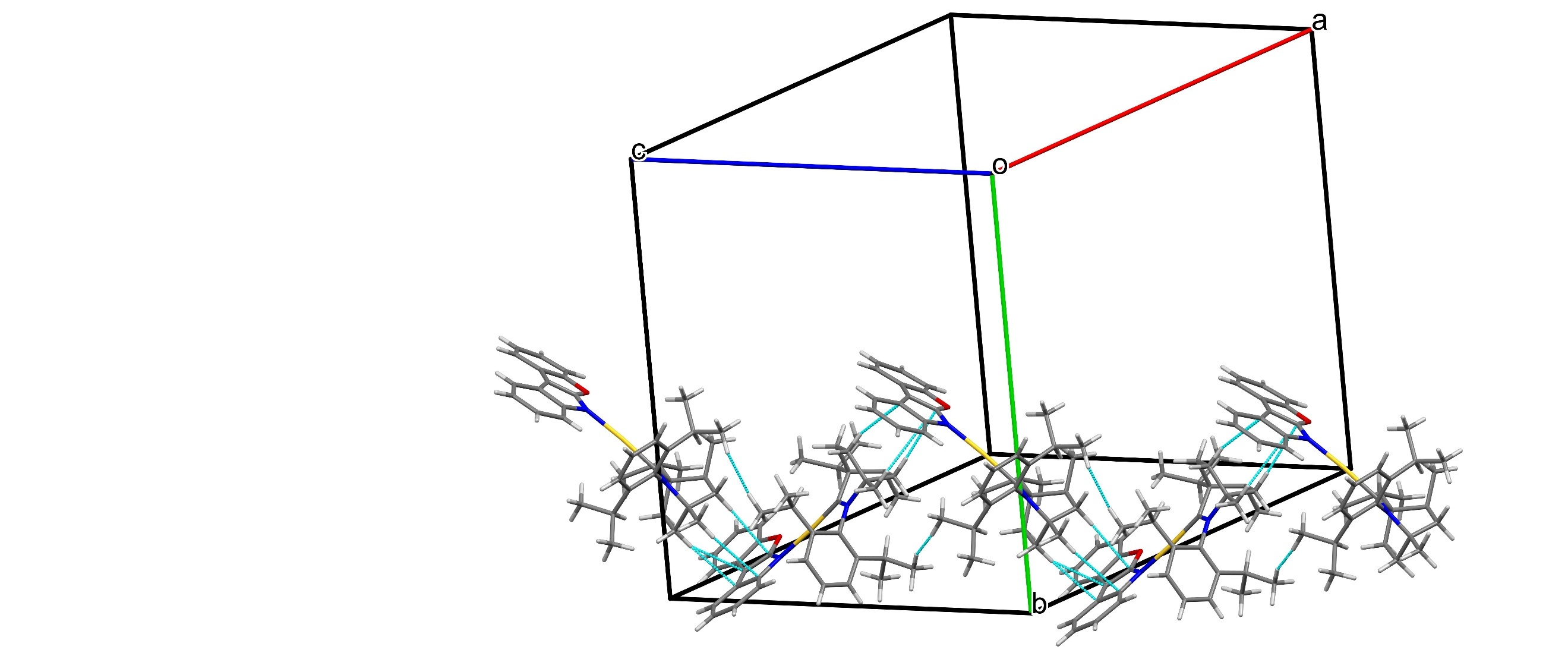


**b)**


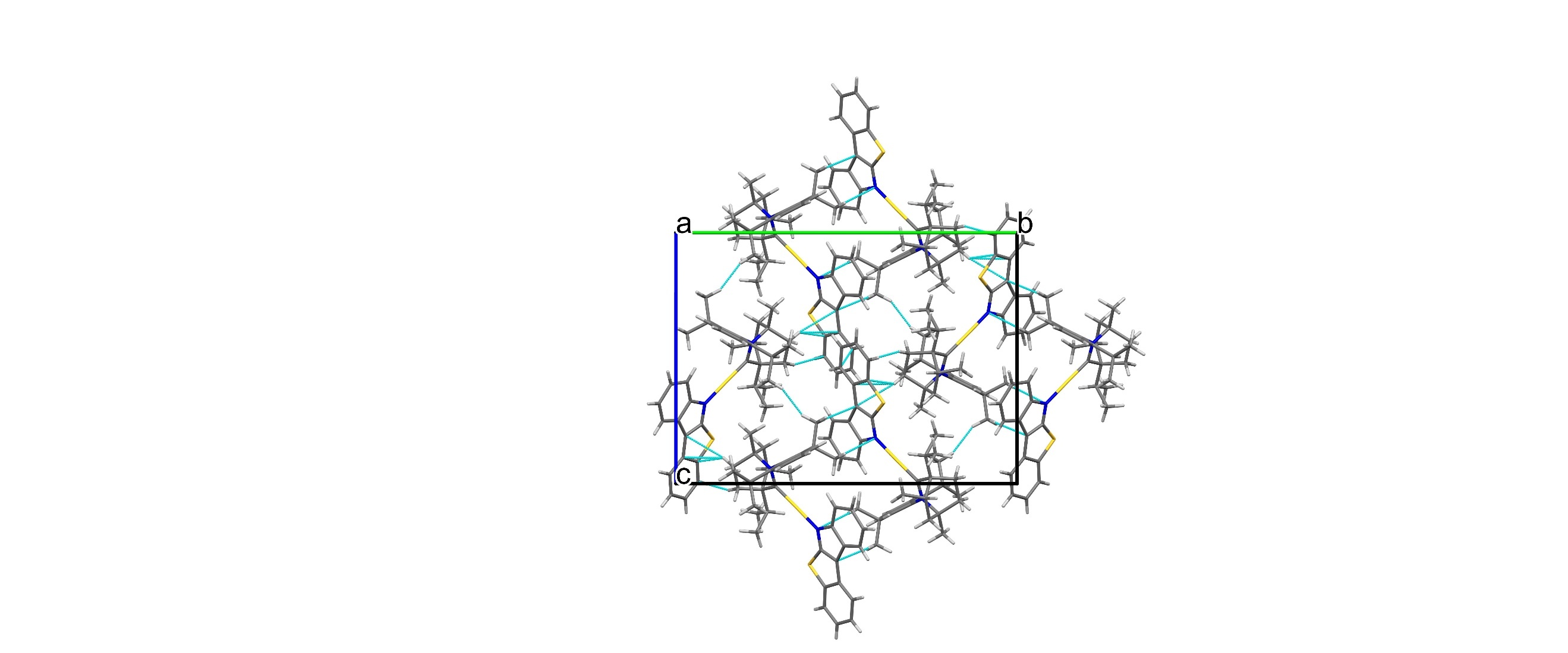


**Figure S6.** Packing diagram for the isomorphous and isostructural complexes **BiCAuBFI** (molecule A with a zigzag chains along crystallographic axis *c*) and **BiCAuBTI** (molecule B, two-dimensional network of molecules in the crystallographic *bc* plane), where blue dashed lines indicate weak intermolecular C–H(carbene)···π(amide) interactions.

**Electrochemistry.**

Cyclic voltammetry (CV) was performed using a three-electrode configuration consisting of a glassy carbon macrodisk working electrode (GCE) (diameter of 3 mm; BASi, Indiana, U.S.A.) combined with a Pt wire counter electrode (99.99%; GoodFellow, Cambridge, U.K.) and an Ag wire pseudoreference electrode (99.99%; GoodFellow, Cambridge, U.K.). The GCE was polished between experiments using alumina slurry (0.3 μm), rinsed in distilled water and subjected to brief sonication to remove any adhering alumina microparticles. The metal electrodes were then dried in an oven at 100 °C to remove residual traces of water, the GCE was left to air dry and residual traces of water were removed under vacuum. The Ag wire pseudoreference electrodes were calibrated to the ferrocene/ferrocenium couple in 1,4-difluorobenzene at the end of each run to allow for any drift in potential, following IUPAC recommendations.^[[8]](#endnote-8)^ All electrochemical measurements were performed at ambient temperatures under an inert N_2_ atmosphere in tetrahydrofuran containing the compound under study (0.14 mM) and the supporting electrolyte [*n*-Bu_4_N][PF_6_] (0.13 mM). Data were recorded with Autolab NOVA software (v. 1.11).

**
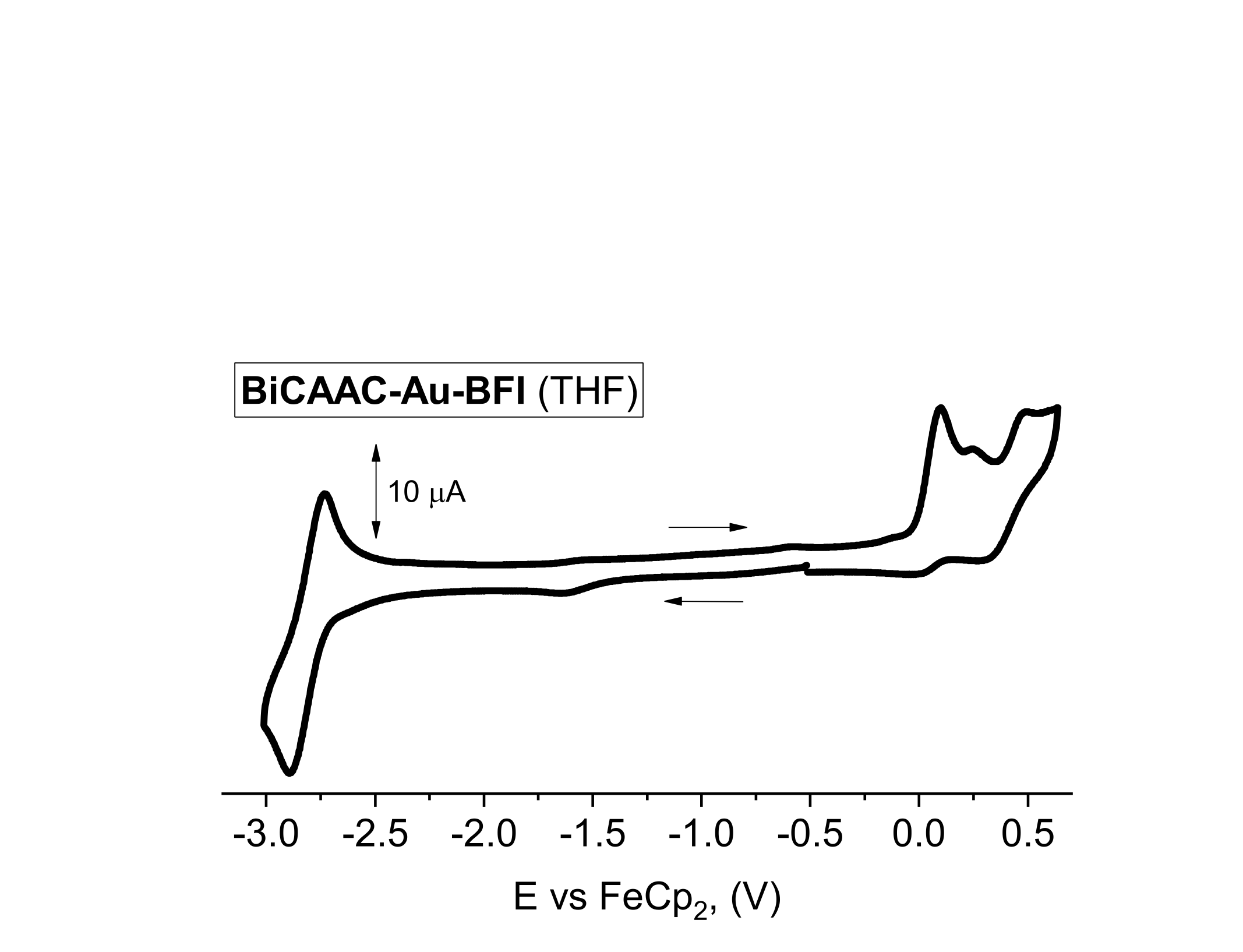
**

**Figure S7.** Full range cyclic voltammogram for **BiCAuBFI**. Recorded using a glassy carbon electrode in tetrahydrofuran solution (1.4 mM) with [n-Bu4N]PF6 as supporting electrolyte (0.13 M), scan rate 0.1 Vs^-1^.


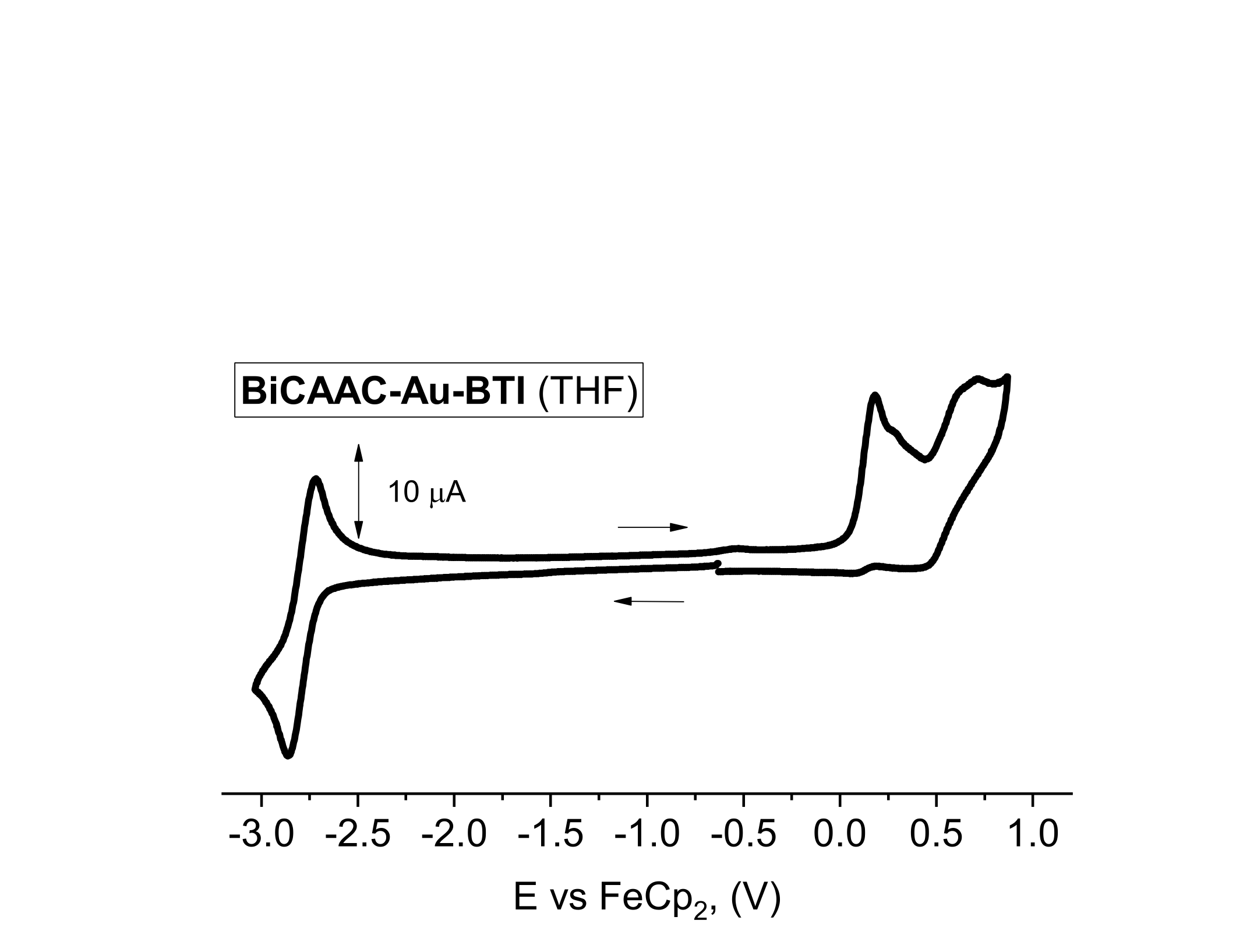


**Figure S8.** Full range cyclic voltammogram for **BiCAuBTI**. Recorded using a glassy carbon electrode in tetrahydrofuran solution (1.4 mM) with [n-Bu4N]PF6 as supporting electrolyte (0.13 M), scan rate 0.1 Vs^-1^.

**Table S1.** Formal electrode potentials (peak position *E*_p_ for irreversible and *E*_1/2_ for quasi-reversible processes (*), *V*, *vs*. FeCp_2_), onset potentials (*E, V*, *vs*. FeCp_2_), peak-to-peak separation in parentheses for quasi-reversible processes (*ΔE_p_* in mV), *E_HOMO_/E_LUMO_* (*e*V) and band gap values (*ΔE*,*e*V) for the redox changes exhibited by **BiCAuBFI** and **BiCAuBTI**.*^a^*

|  | Reduction | | *E_LUMO_*  eV | Oxidation | | | | *E_HOMO_*  eV | *ΔE*  eV |
| --- | --- | --- | --- | --- | --- | --- | --- | --- | --- |
|  | *E*_1st_ | *E_onset red_* |  | *E*_1st_ | *E_onset ox_* | *E*_2nd_ | *E*_3rd_ |  |  |
| **BiCAuBFI** | -2.81  (160) | -2.71 | -2.68 | +0.10 | +0.01 | +0.24 | +0.49 | -5.40 | 2.72 |
| **BiCAuBTI** | -2.79  (140) | -2.71 | -2.68 | +0.18 | +0.06 | +0.28 | +0.71 | -5.45 | 2.77 |

*^a^* In THF solution, recorded using a glassy carbon electrode, concentration 1.4 m*M*, supporting electrolyte [*^n^*Bu_4_N][PF_6_] (0.13 *M*), measured at 0.1 V s^−1^.*^b^* *E*_HOMO_*=*–(*E*_onset ox Fc/Fc_*_+_+*5.39) *e*V; *E*_LUMO_*=*– (*E*_onset red Fc/Fc+_*+*5.39) eV (*Adv. Mater.* 2011, **23**, 2367–2371).

**Photophysical Characterisation.**

UV-visible absorption spectra were recorded using a Varian Cary 5000 UV-Vis-NIR spectrometer. Photoluminescence measurements were recorded on an Edinburgh Instruments FLS980 spectrometer with a solids mount attachment where appropriate. Absolute photoluminescence quantum yields were recorded using an Hamamatsu Quantaurus-QY C11347-11. Quantum yields have been measured in air for solid samples and under argon for solutions. Time resolved luminescence data were collected on a time-correlated single photon counting (TCSPC) Edinburgh Instruments FLS980 spectrometer using F-900 software. A xenon flash lamp and EPL pulsed diode lasers were used as excitation sources. The collected data were analysed using F-900 software.


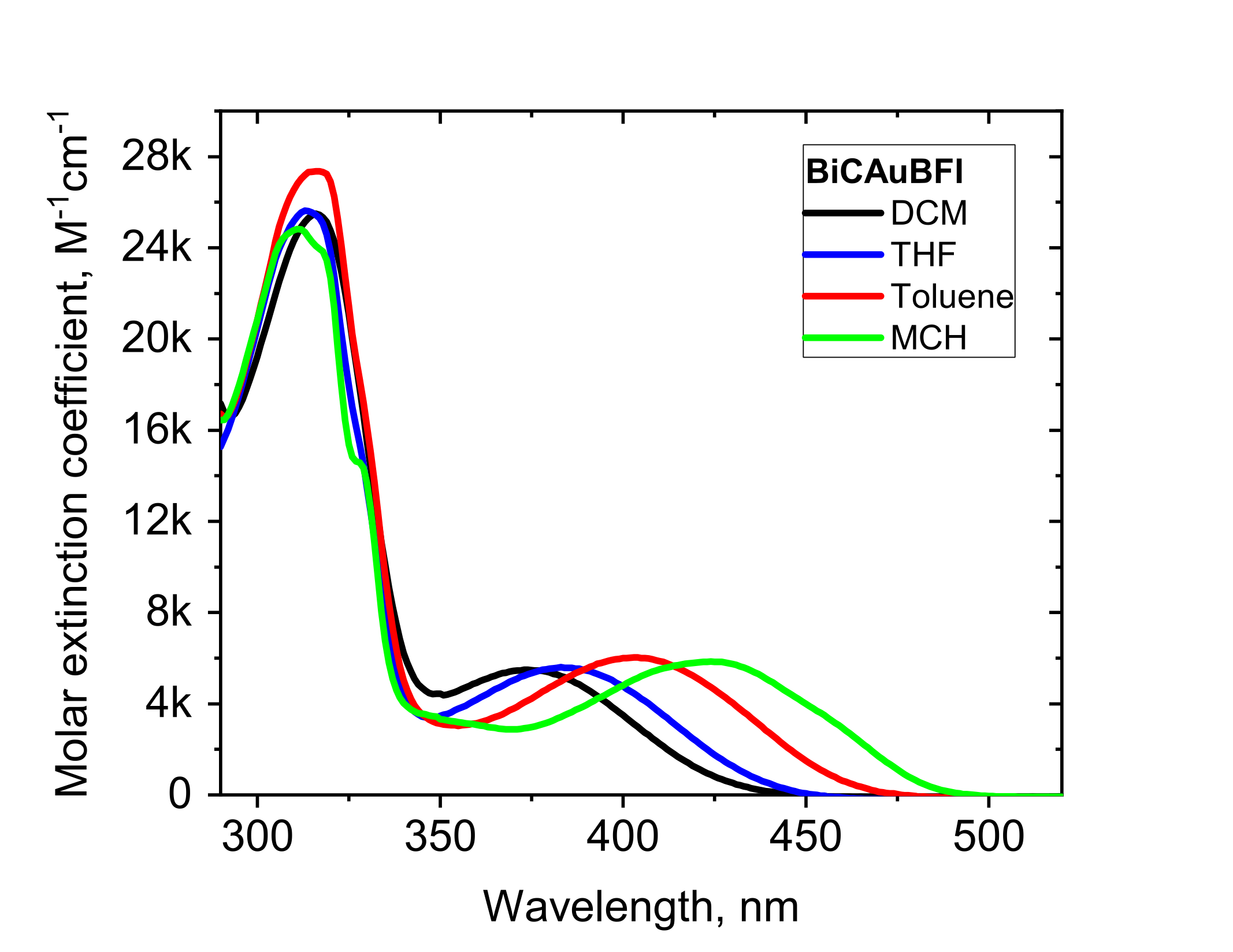


**Figure S9.** UV-Vis spectra of **BiCAuBFI** in various solvents at 295K.


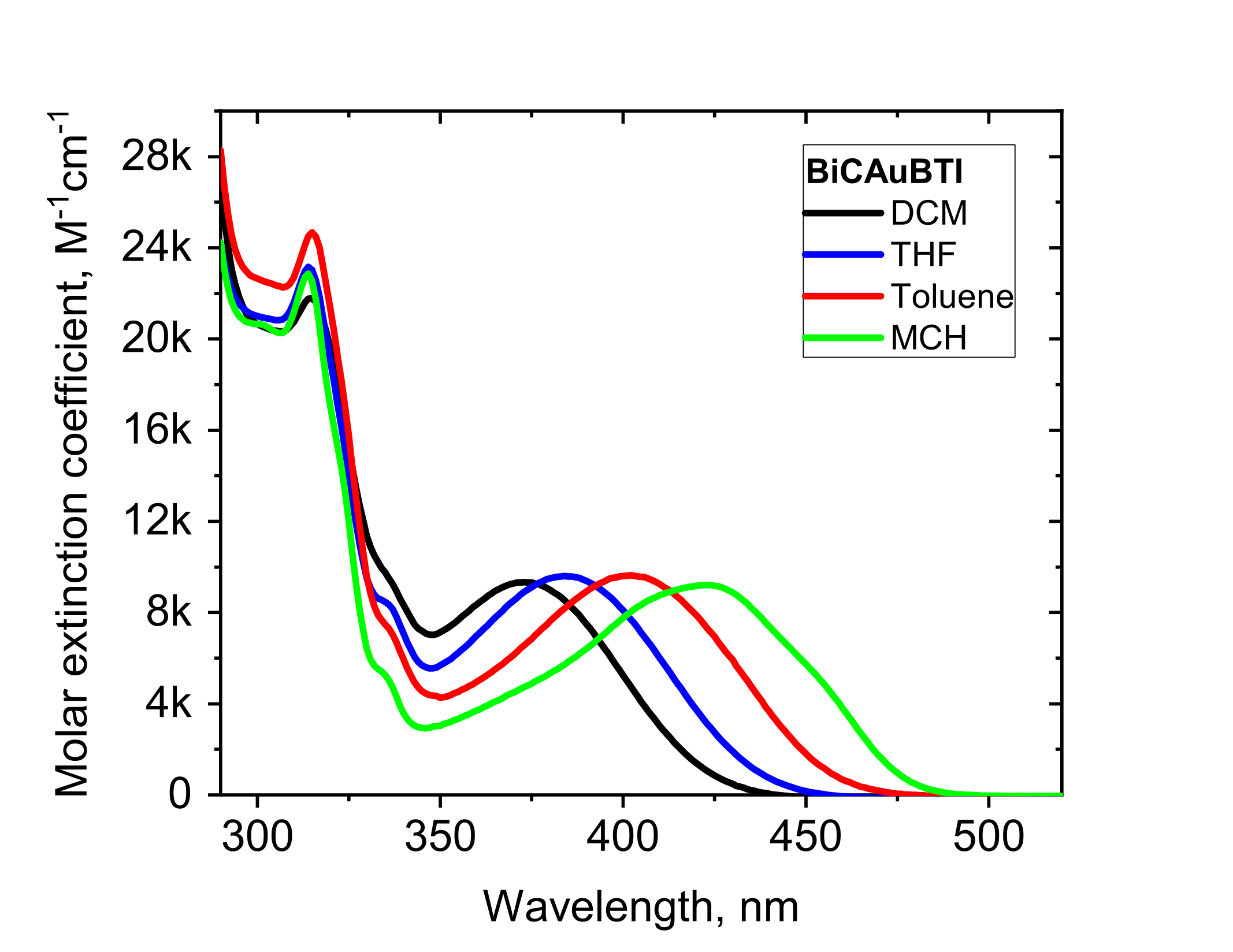


**Figure S10.** UV-Vis spectra of **BiCAuBTI** in various solvents at 295K.

**Table S2.** UV-Vis data for **BiCAuBFI** and **BiCAuBTI** in various solvents.

|  | *λ*_abs_ [nm], (10^3^ *ε*/M^−1^ cm^−1^) | | | |
| --- | --- | --- | --- | --- |
|  | DCM | THF | Toluene | MCH |
| **BiCAuBFI** | 375 (5.5)  316 (25.5)  2.89 eV* | 384 (5.6)  313 (25.6)  2.82 eV* | 403 (6.0)  316 (27.4)  2.68 eV* | 424 (5.9)  311 (24.8)  2.56 eV* |
| **BiCAuBTI** | 373 (9.3)  336 (9.5) sh  315 (21.8)  2.93 eV* | 384 (9.6)  336 (8.3) sh  314 (23.2)  2.83 eV* | 402 (9.6)  337 (7.2) sh  315 (24.6)  2.72 eV* | 423 (9.2)  335 (5.2) sh  314 (22.9)  2.60 eV* |
| *Red-edge onset values | | | | |

**Table S3.** Photophysical properties of **BiCAuBFI** and **BiCAuBTI** in various matrices.

|  | λ_em_ (nm) | τ  (ns) | Φ  (%)^a^ | k_r_  (10^6^ s^−1^)^b^ | k_nr_  (10^6^ s^−1^)^c^ | CT/LE  (eV)^d^ | λ_em_  (nm, 77K) | τ  (µs, 77K) |
| --- | --- | --- | --- | --- | --- | --- | --- | --- |
| 1 wt% PS matrix | | | | | |  | | |
| **BiCAuBFI** | 511 | 473 | 100 | 2.11 | – | 2.75 (2.83)^e^ | 492 | 23.4 (16%)  65.3 (84%) |
| **BiCAuBTI** | 501 | 715 | 100 | 1.40 | – | 2.78 (2.81)^e^ | 488 | 48.2 (26%)  87.7 (74%) |
| Toluene Solution | | | | | | | | |
| **BiCAuBFI** | 582 | 88.7 | 39 | 4.40 | 6.88 | 2.54/2.87 | 482 | 63.2 |
| **BiCAuBTI** | 558 | 340 | 71 | 2.09 | 0.85 | 2.61/2.91 | 469 | 69.9 |
| Methylcyclohexane Solution | | | | | | | | |
| **BiCAuBFI** | 543 | 367 | 58 | 1.58 | 1.14 | 2.62/2.93 | 467 | 63.9 |
| **BiCAuBTI** | 529 | 740 | 85 | 1.15 | 0.20 | 2.65/2.94 | 435 | 62.4 |

*^a^* Quantum yields determined using an integrating sphere; *^b^*radiative rate constant *k*_r_ = *Φ/ τ*; *^c^* Nonradiative constant *k*_nr_ = (1 – *Φ*)*/ τ.* In case of two-component lifetime *τ* an average was used: *τ_av_ =(B_1_/(B_1_ +B_2_*))*τ_1_ + (B_2_/(B_1_ +B_2_*))*τ_2_,* where *B*_1_ and *B*_2_ are the relative amplitudes for *τ_1_* and *τ_2_*, respectively; *^d^* CT/LE energies based on the onset values of the emission spectra blue edges at 295 K and 77 K, respectively; *^e^* CT energies based on the onset values of the emission spectra blue edges at 77 K.

**
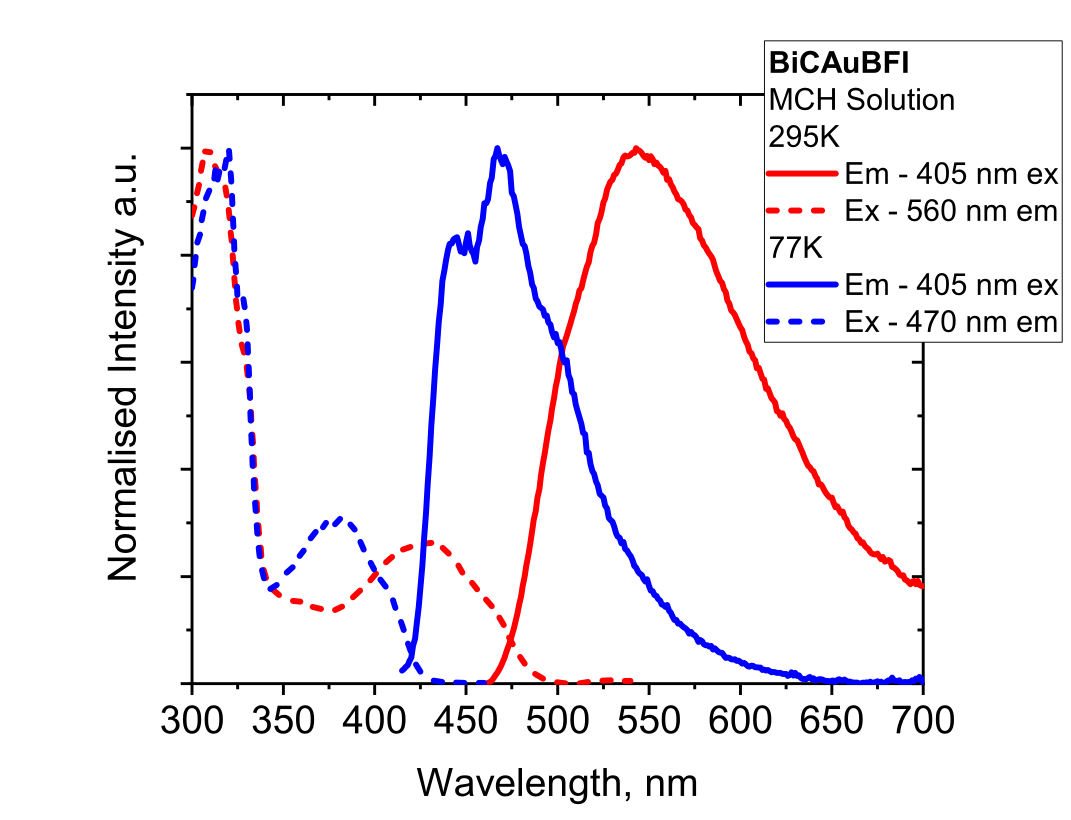

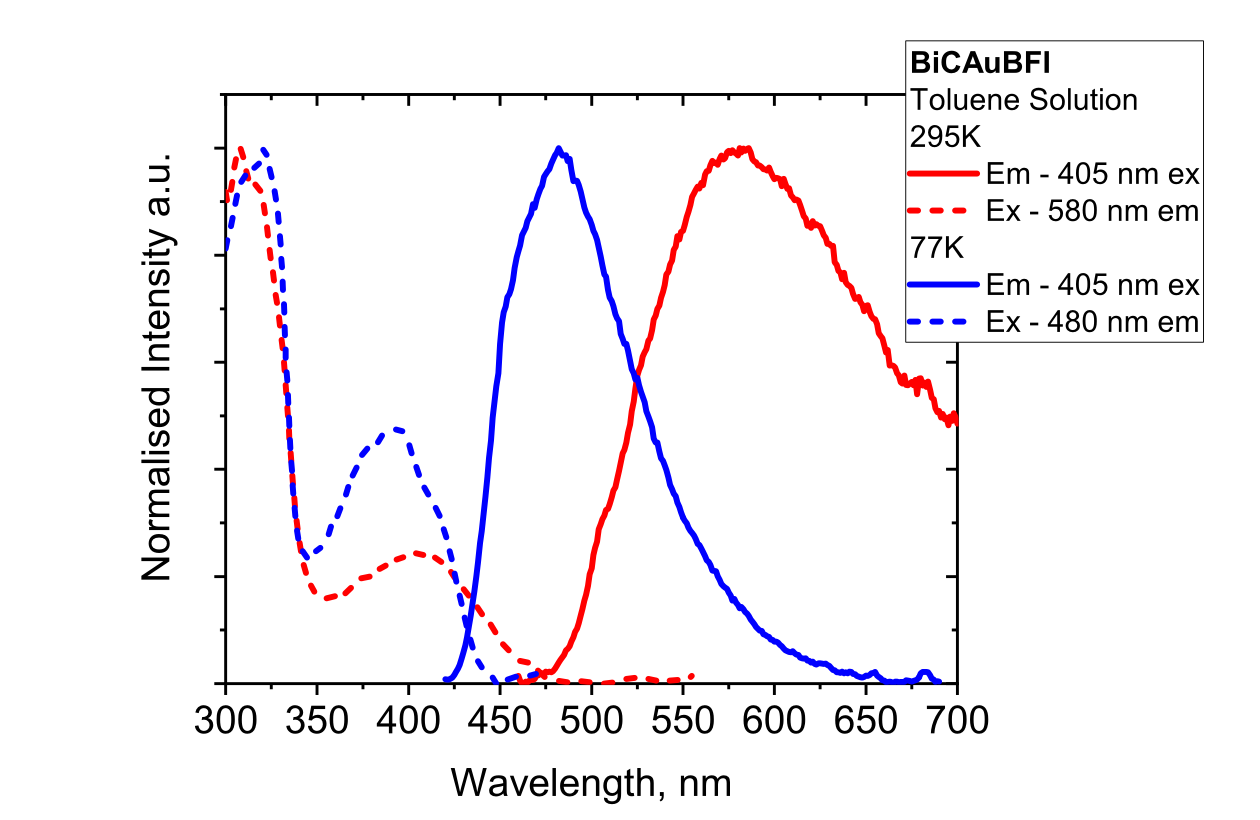

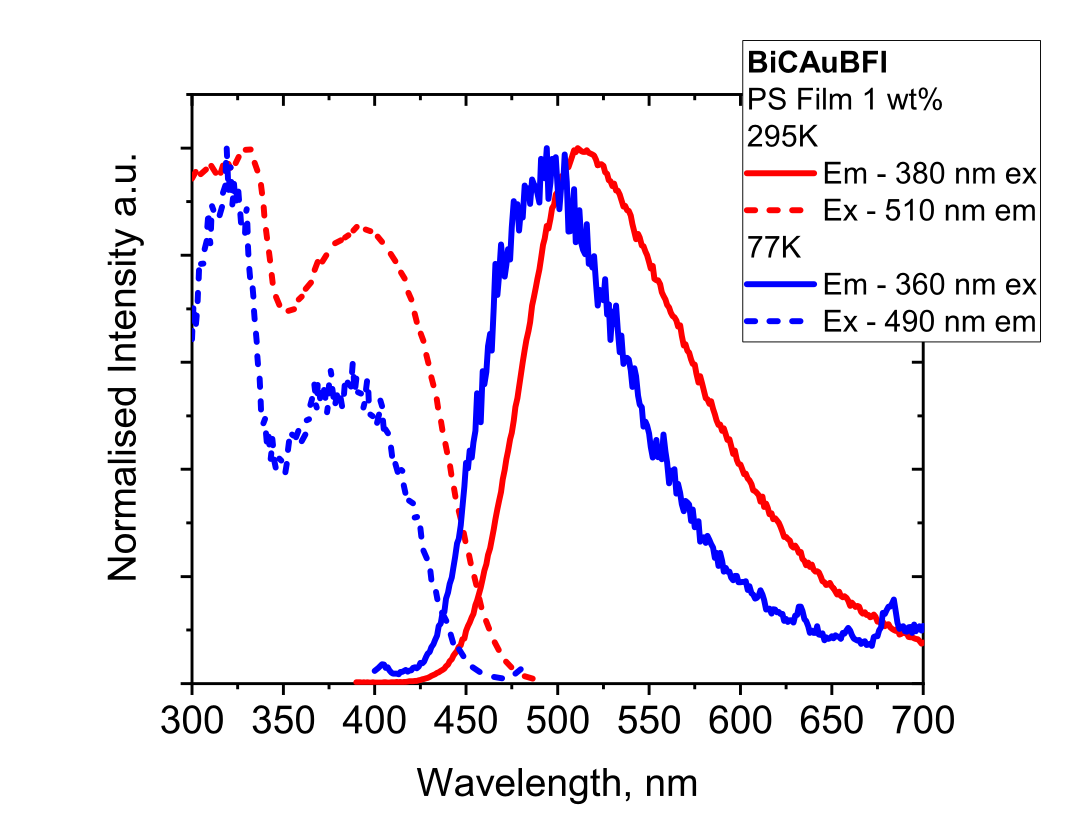
**

**Figure S11.** Photoluminescent spectra of **BiCAuBFI** in methylcyclohexane solution (top left), toluene solution (top right) and 1 wt% PS films (bottom) at 295K (red) and 77K (blue); excitation – dotted, emission – solid.

**
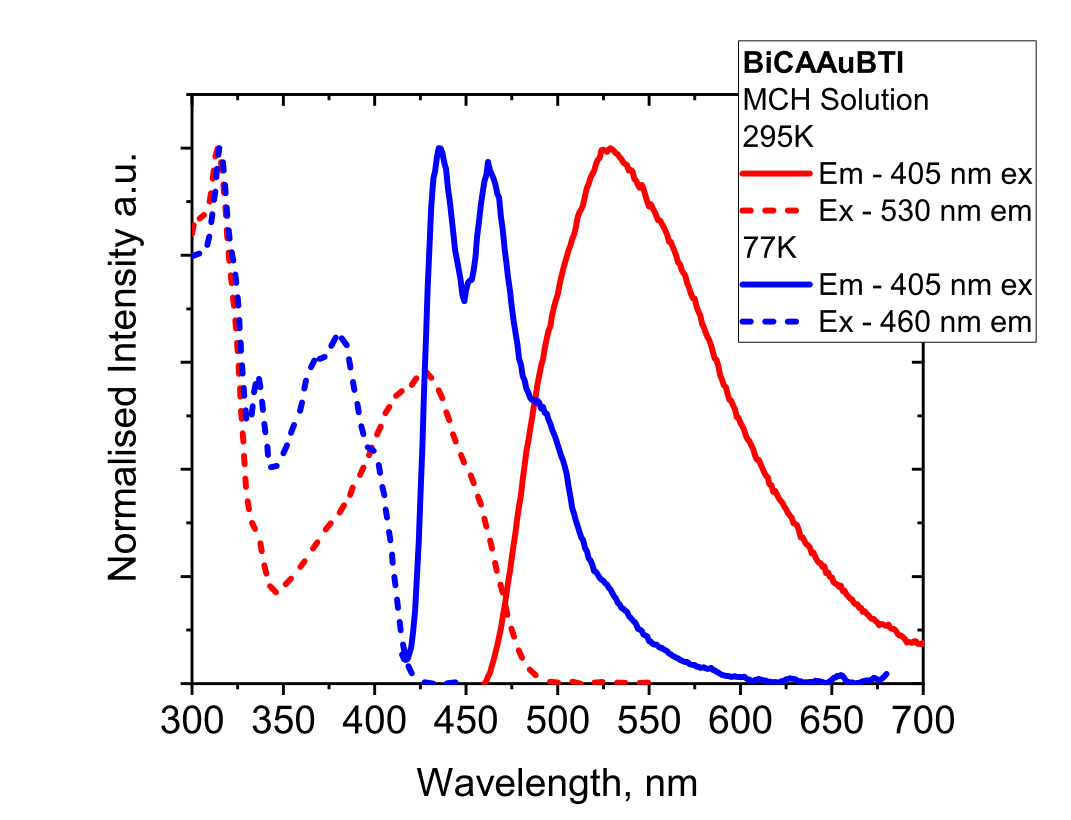

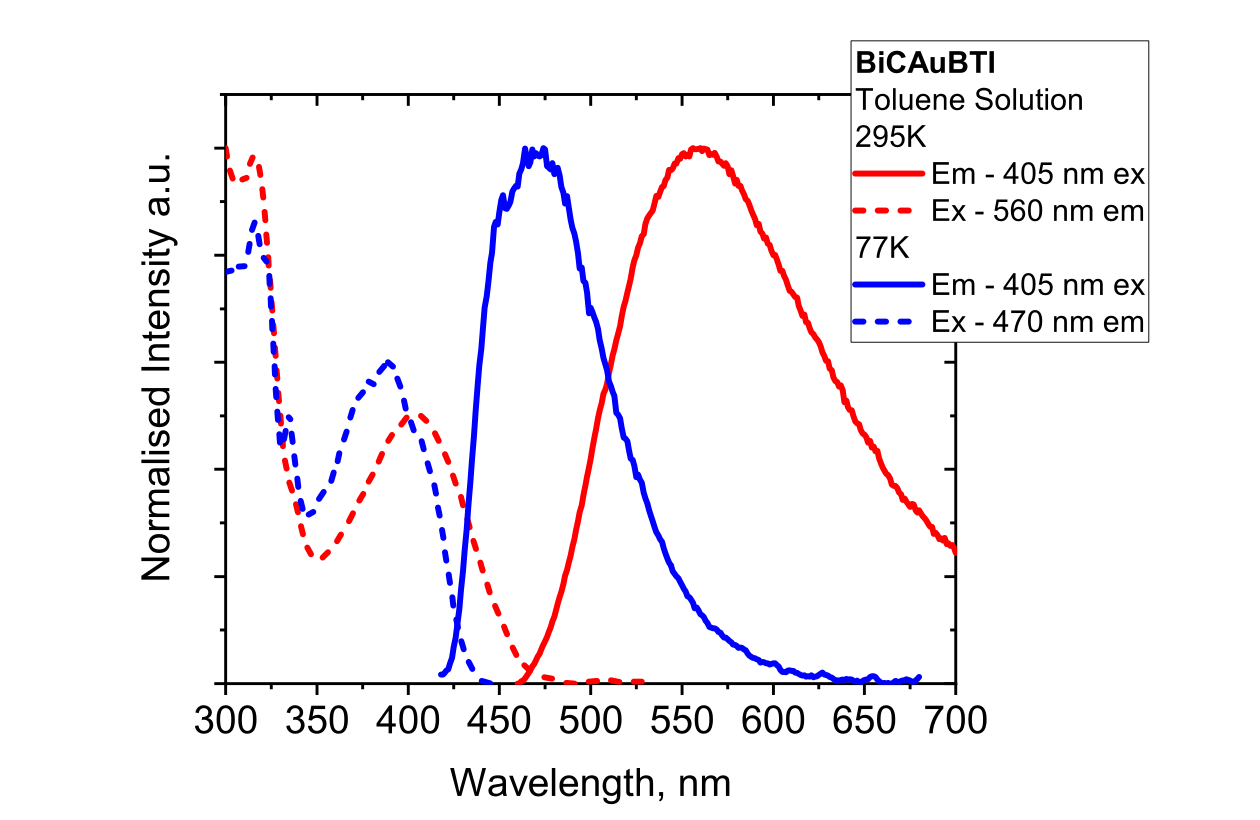

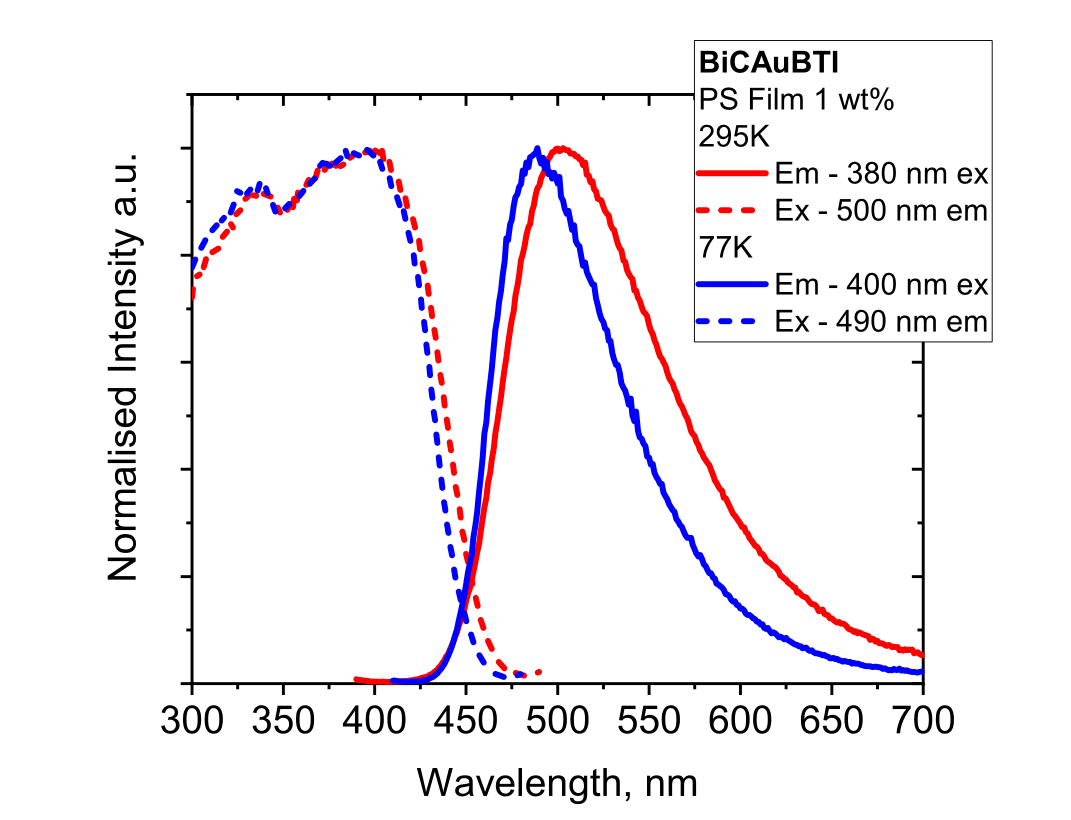
**

**Figure S12.** Photoluminescent spectra of **BiCAuBTI** in methylcyclohexane solution (top left), toluene solution (top right) and 1 wt% PS films (bottom) at 295K (red) and 77K (blue); excitation – dotted, emission – solid.

**Figure S13.** Arrhenius plot to estimate thermal activation energy barrier for new CMA complexes **BiCAuBFI** (top) and **BiCAuBTI** (bottom).

**Computational Details**

The ground states of the complexes were studied by density functional theory (DFT) and the excited states by time-dependent DFT (TD-DFT) using the Tamm-Dancoff approximation.^[[9]](#endnote-9),^^[[10]](#endnote-10)^ Calculations were carried by the global hybrid MN15 functional of the Minnesota series by Truhlar and coworkers, which has especially good performance for noncovalent interactions and excitation energies.^[[11]](#endnote-11)^ The def2-TZVP basis set^[[12]](#endnote-12),^^[[13]](#endnote-13)^ was employed with relativistic effective core potential of 60 electrons for description of the core electrons of Au.^[[14]](#endnote-14)^ We have previously employed the selected methodology with success for closely related molecules.^[[15]](#endnote-15),^^[[16]](#endnote-16)^ For TD-DFT spin-orbit coupling calculations,^17^ an all-electron scalar relativistic SARC-ZORA-TZVP basis set was employed for gold.^18^ Gold metal contributions to HOMO and LUMO were calculated by the Mulliken population analysis and HOMO-LUMO overlap integrals were calculated using Multiwfn program.^19^ All calculations were carried out by Gaussian 16,^20^ except TD-DFT spin-orbit coupling calculations were performed using Orca 6.0.0.^21^

**Table S4**. Molecular orbital distribution of the HOMO and LUMO for gold complexes in S_0_ geometry, showing contributions of the metal orbitals and HOMO-LUMO overlap integrals.

|  | HOMO | LUMO |
| --- | --- | --- |
| 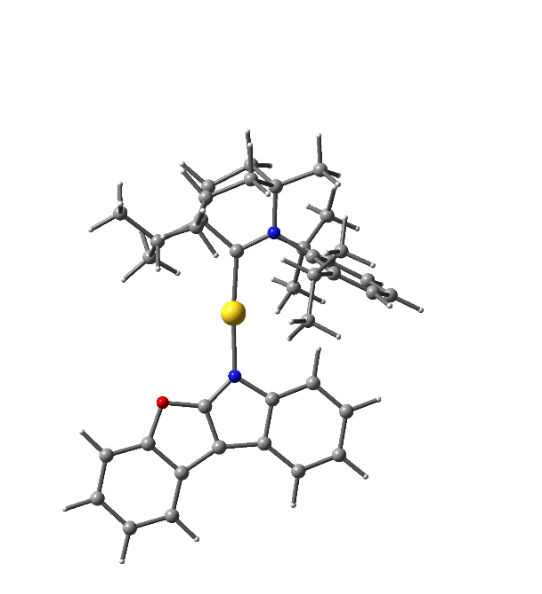  **BiCAuBFI**  Overlap integral: 0.29 | 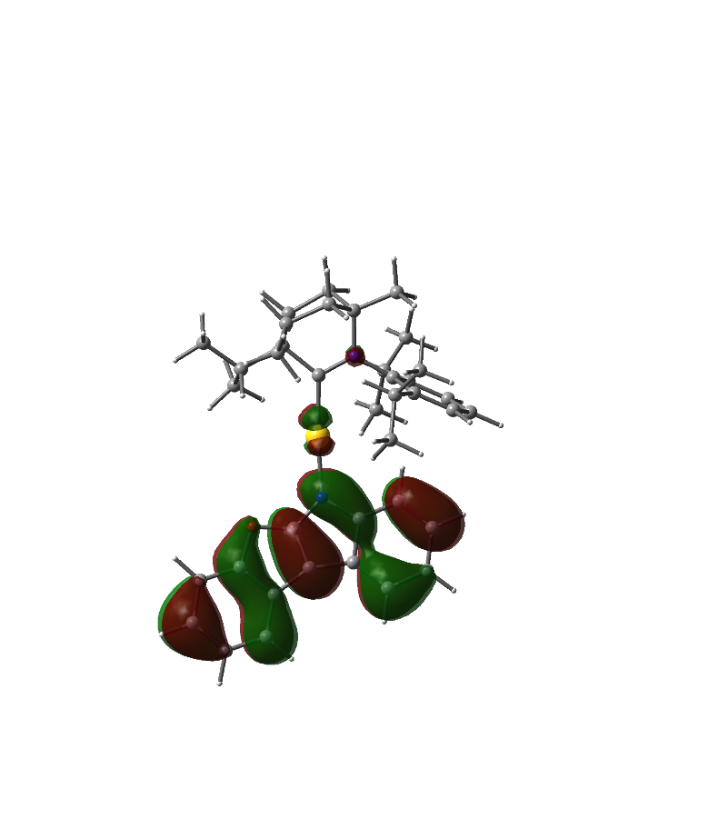  1.4%Au | 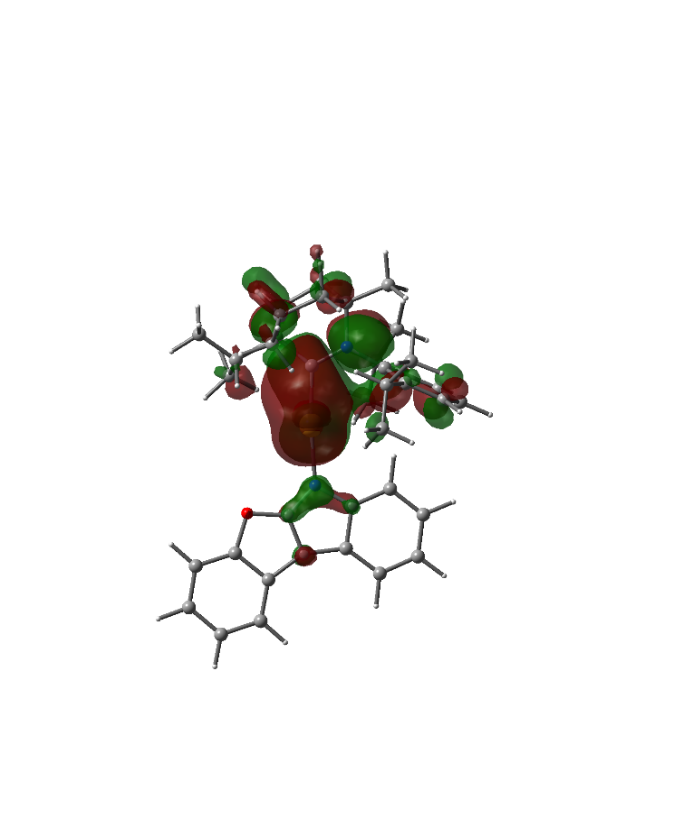  11.1%Au |
| 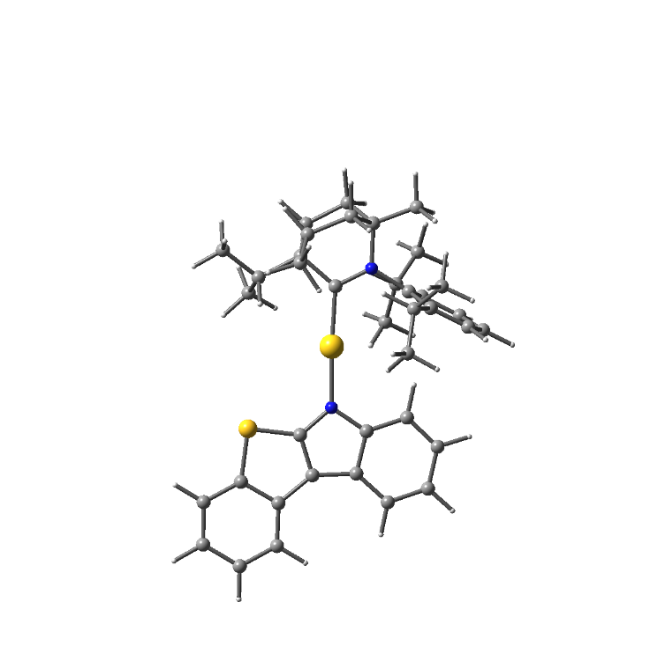  **BiCAuBTI**  Overlap integral: 0.31 | 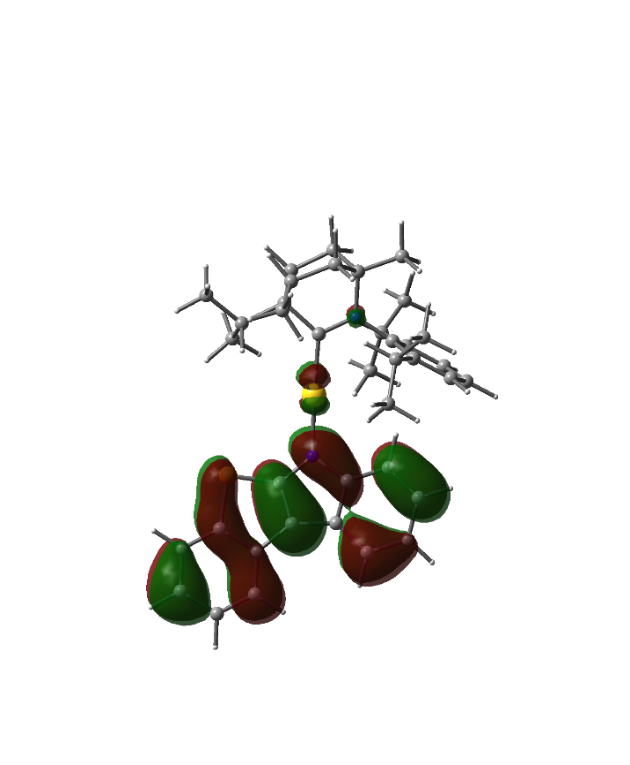  1.5%Au | 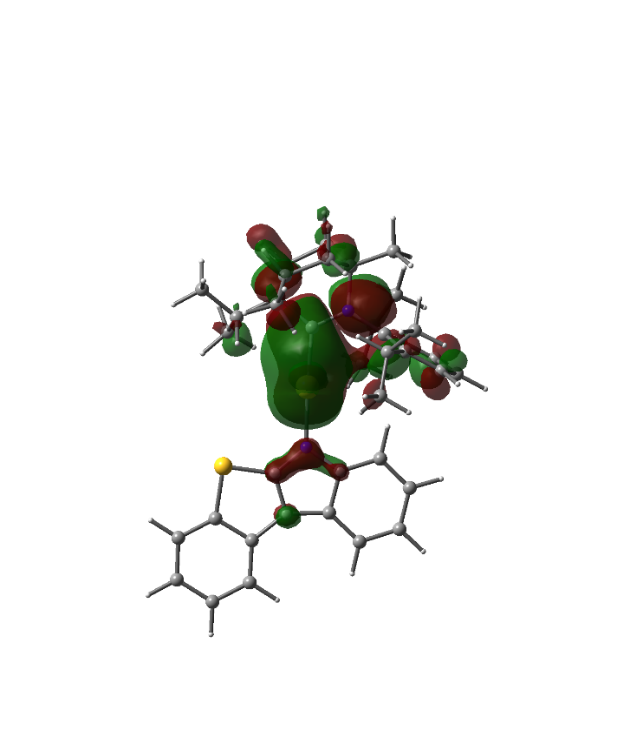  11.5%Au |

**Table S5**: Calculated bond dissociation energies in optimised S_0_ geometry.

|  | Au-C | | Au-N | |
| --- | --- | --- | --- | --- |
|  | kJ/mol | eV | kJ/mol | eV |
| **BiCAuBFI** | 411.6 | 4.27 | 347.4 | 3.60 |
| **BiCAuBTI** | 410.9 | 4.26 | 359.2 | 3.72 |

**Table S6.** Calculated dipole moments in S_0_ and S_1_ states in S_0_ geometry.

|  | S_0_ | S_1_@S_0_ |
| --- | --- | --- |
| **BiCAuBFI** | 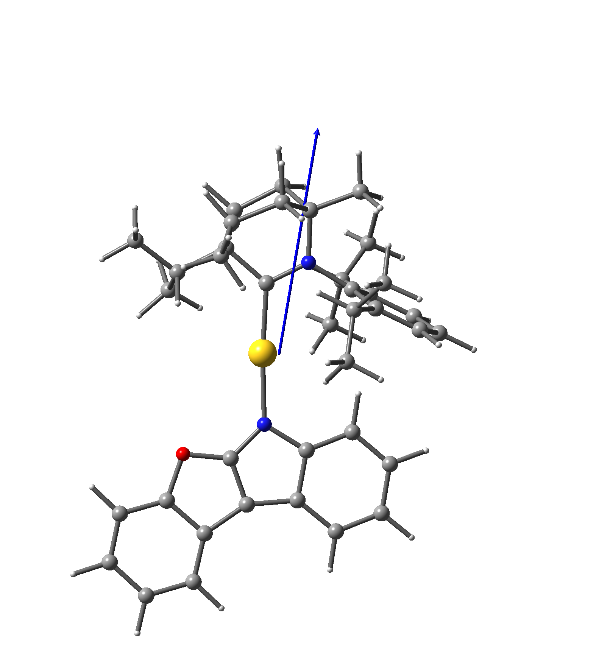  11.7D | 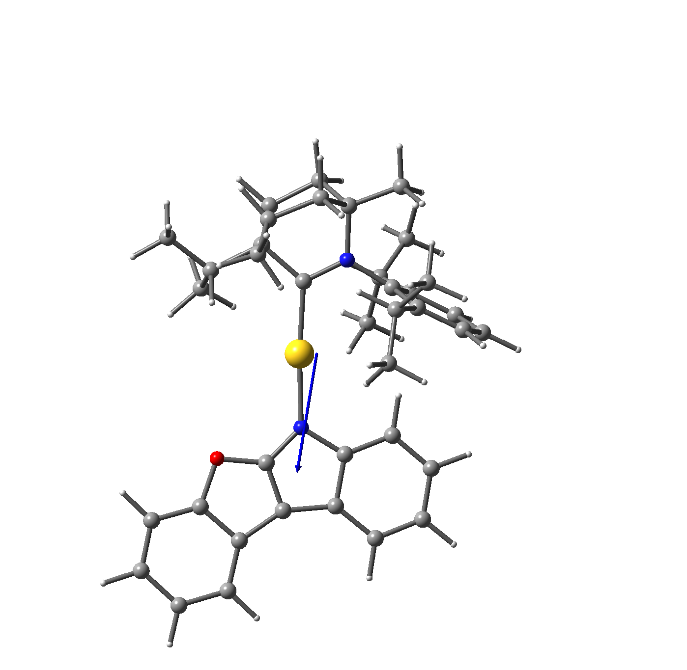  (-)5.9D |
| **BiCAuBTI** | 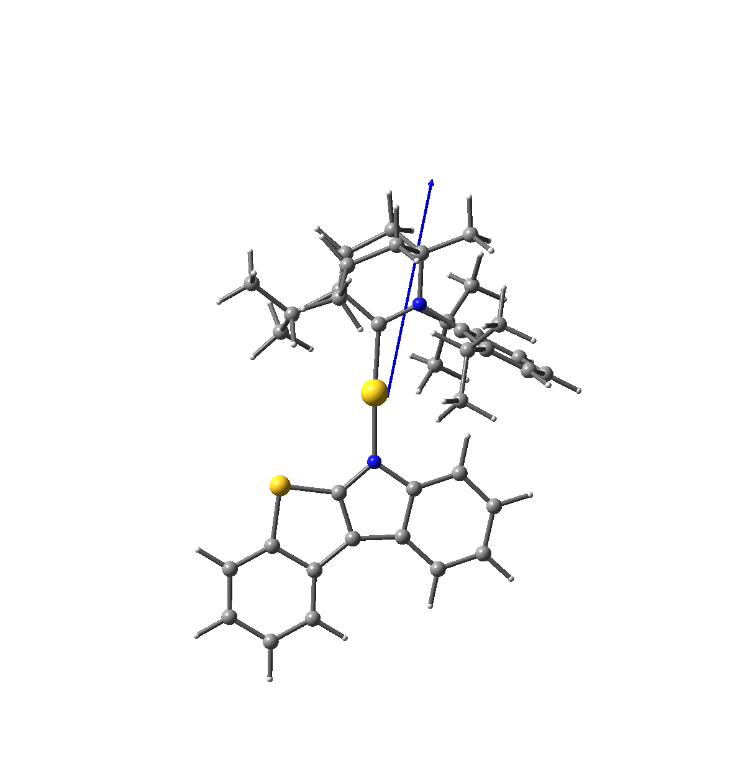  11.7D | 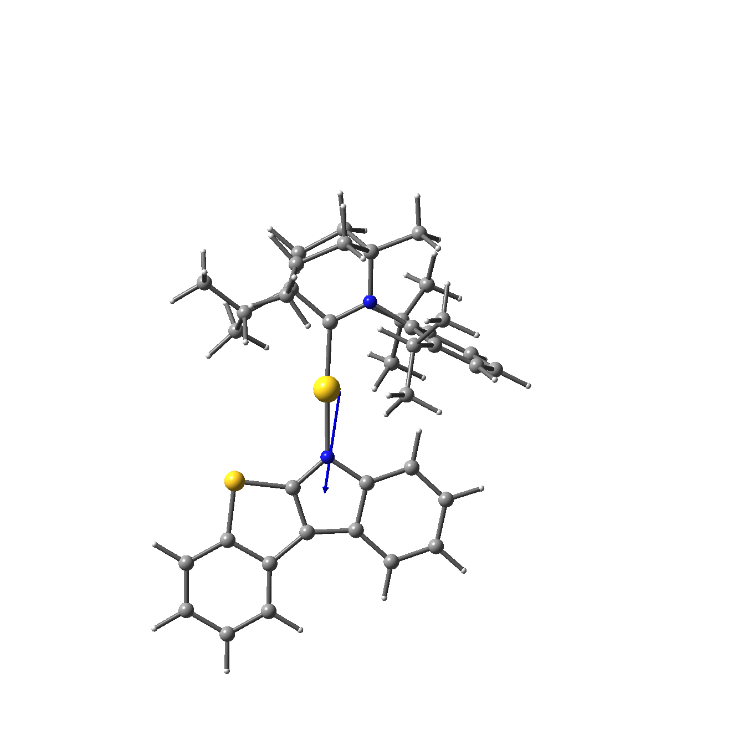  (-)5.5D |

**Table S7** Calculated vertical excitations, their character and S_0_-S_1_ oscillator strength coefficients.

|  | Excitation energy | Character | Oscillator strength |
| --- | --- | --- | --- |
| **BiCAuBFI** | S_1_ (^1^CT): 2.86eV = 433nm | HOMO – LUMO (98%) | 0.1515 |
|  | T_1_ (^3^CT): 2.66eV = 466nm | HOMO – LUMO (94%) |  |
|  | T_2_ (^3^CT): 3.36eV = 369nm | HOMO-1 – LUMO (88%) |  |
|  | T_3_ (^3^LE(amide)): 3.58eV = 347nm | HOMO – LUMO+3 (82%) |  |
| **BiCAuBTI** | S_1_ (^1^CT): 2.94eV = 422nm | HOMO – LUMO (98%) | 0.1711 |
|  | T_1_ (^3^CT): 2.71eV = 458nm | HOMO – LUMO (90%) |  |
|  | T_2_ (^3^CT): 3.20eV = 387nm | HOMO-1 – LUMO (86%) |  |
|  | T_3_ (^3^LE(amide)): 3.56eV = 349nm | HOMO – LUMO+4 (65%) |  |

**Table S8.** Calculated highest occupied natural transition orbitals (HONTOs) and lowest unoccupied natural transition orbitals (LUNTOs) for vertical excited states.

|  |  | HONTO | LUNTO |
| --- | --- | --- | --- |
| **BiCAuBFI** | S_1_ | 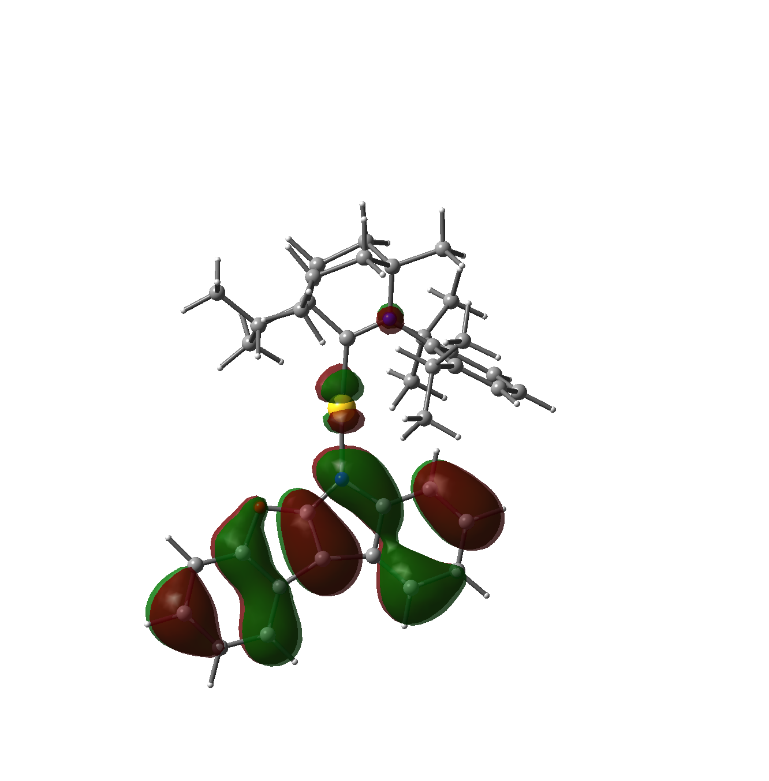 | 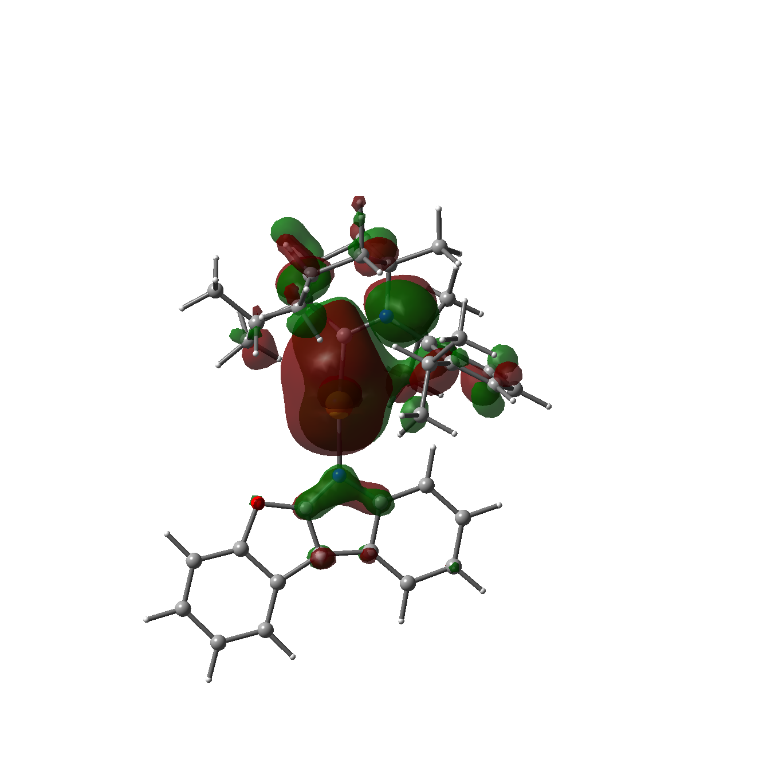 |
|  | T_1_ | 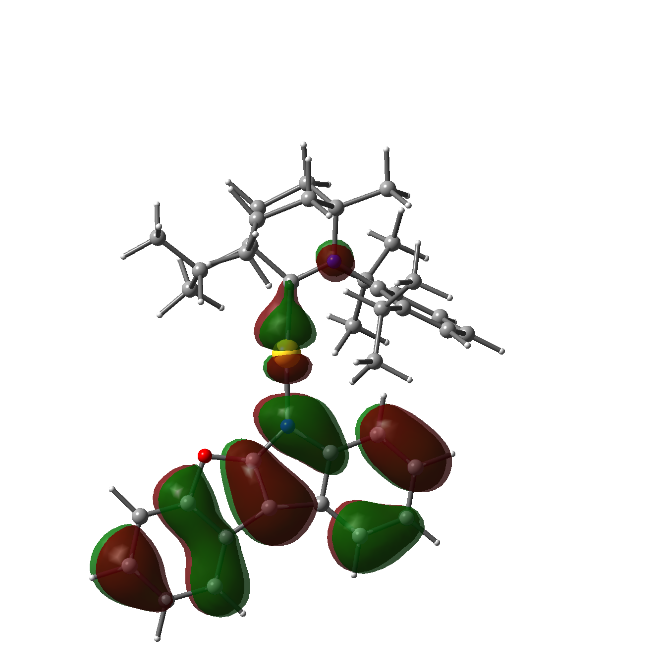 | 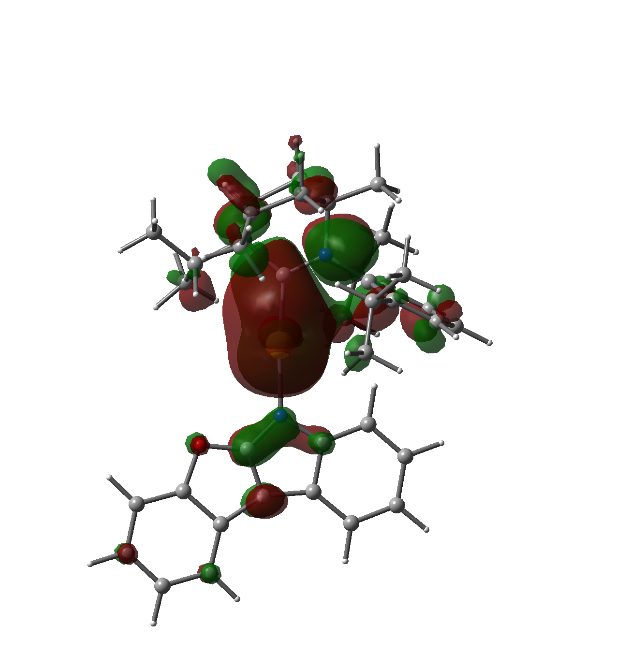 |
|  | T_2_ | 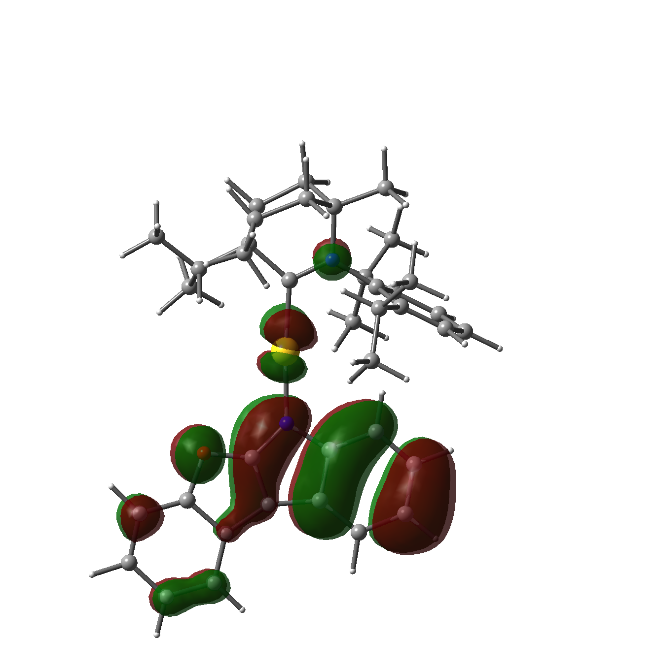 | 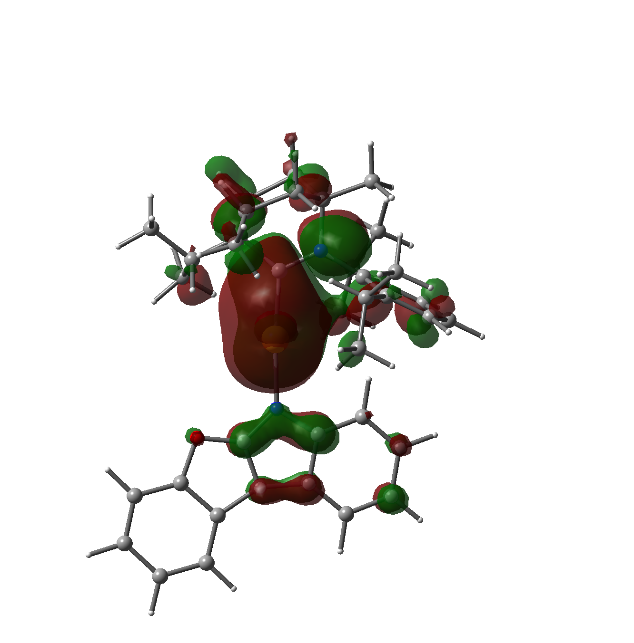 |
|  | T_3_ | 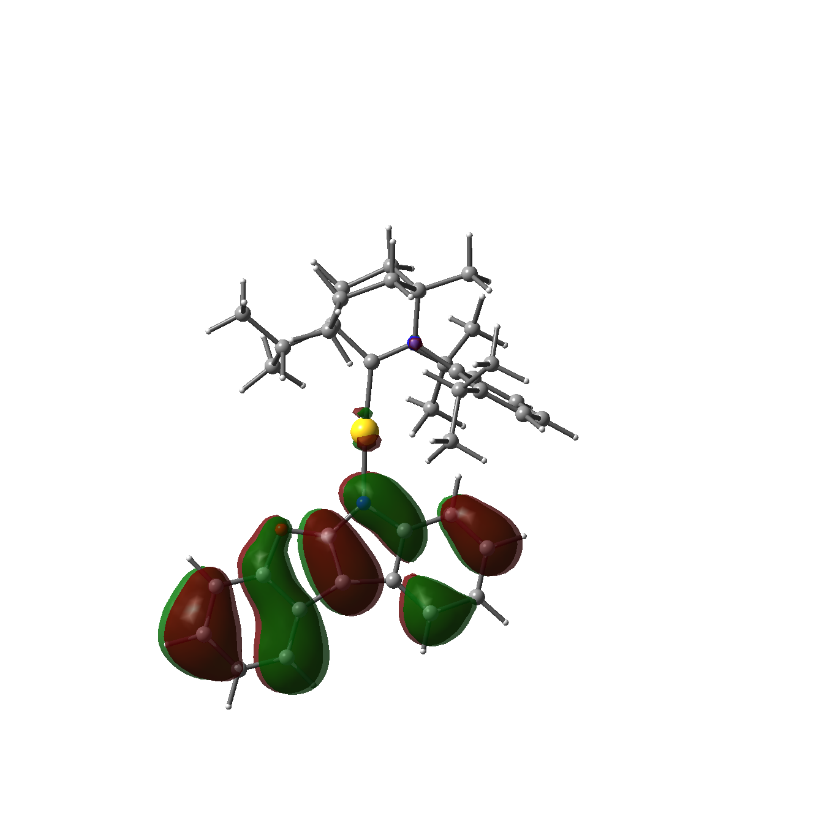 | 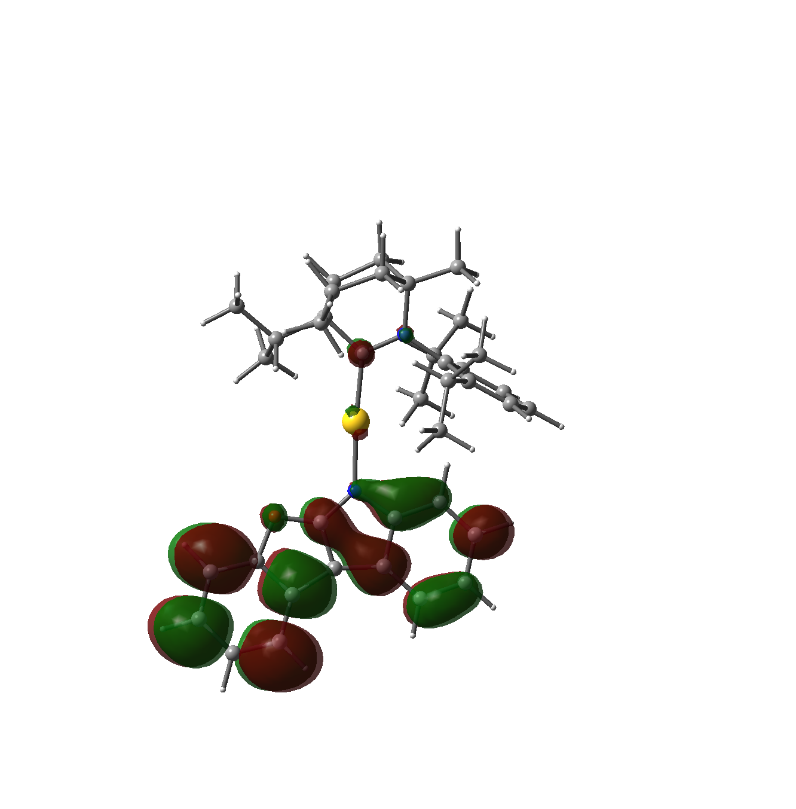 |
| **BiCAuBTI** | S_1_ | 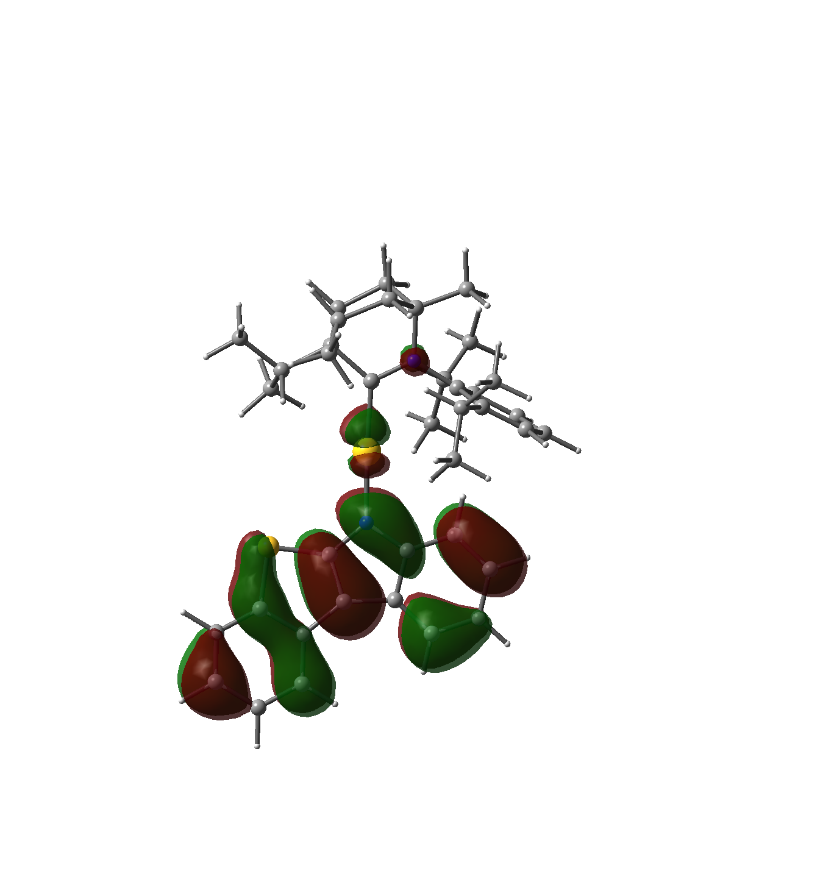 | 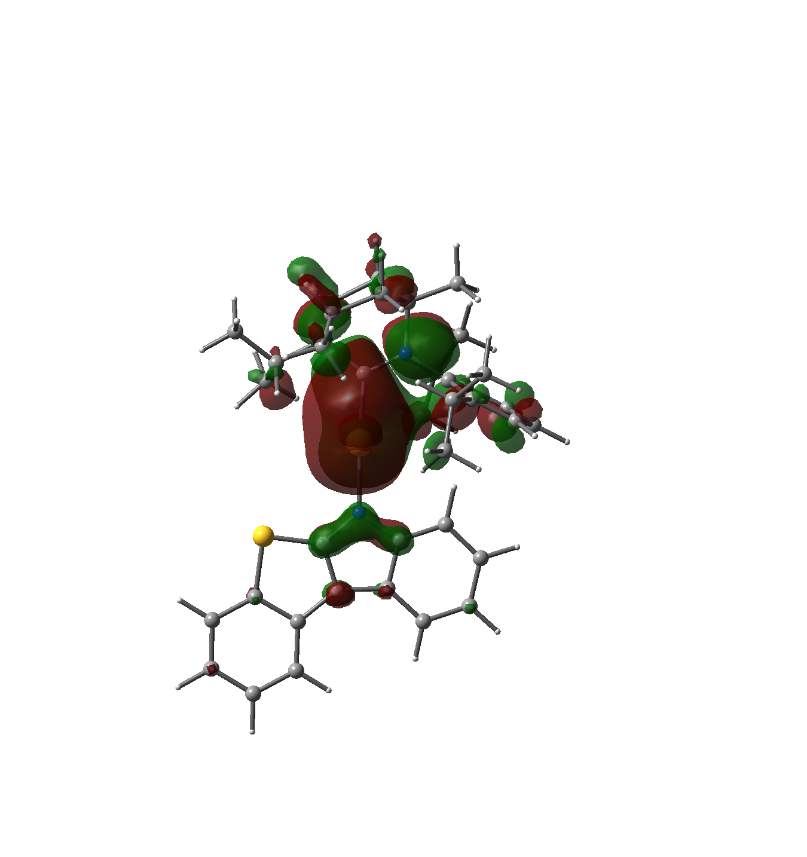 |
|  | T_1_ | 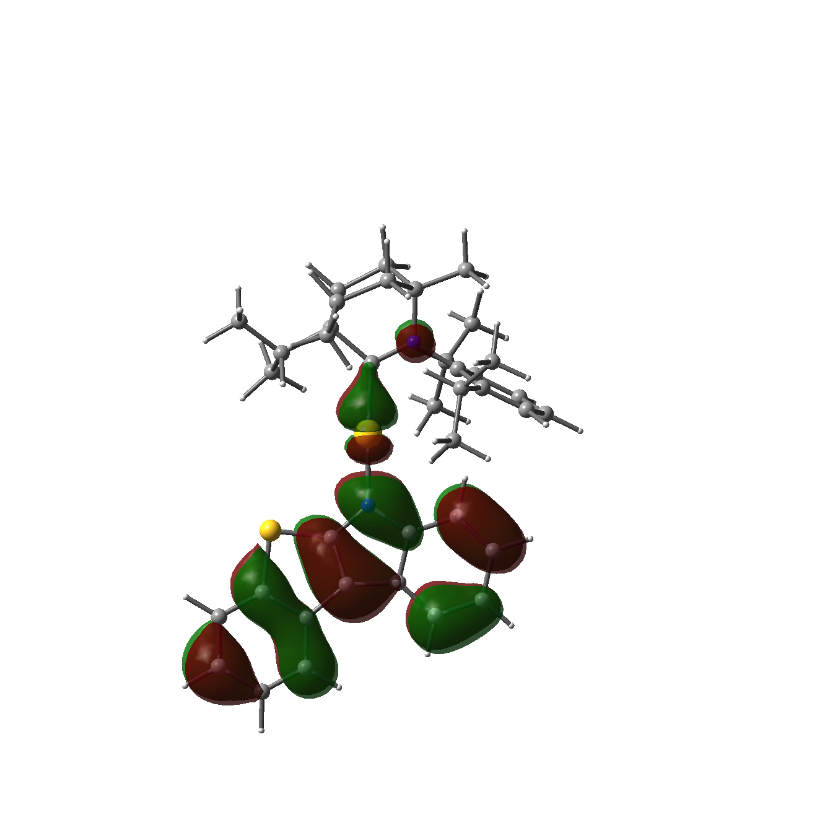 | 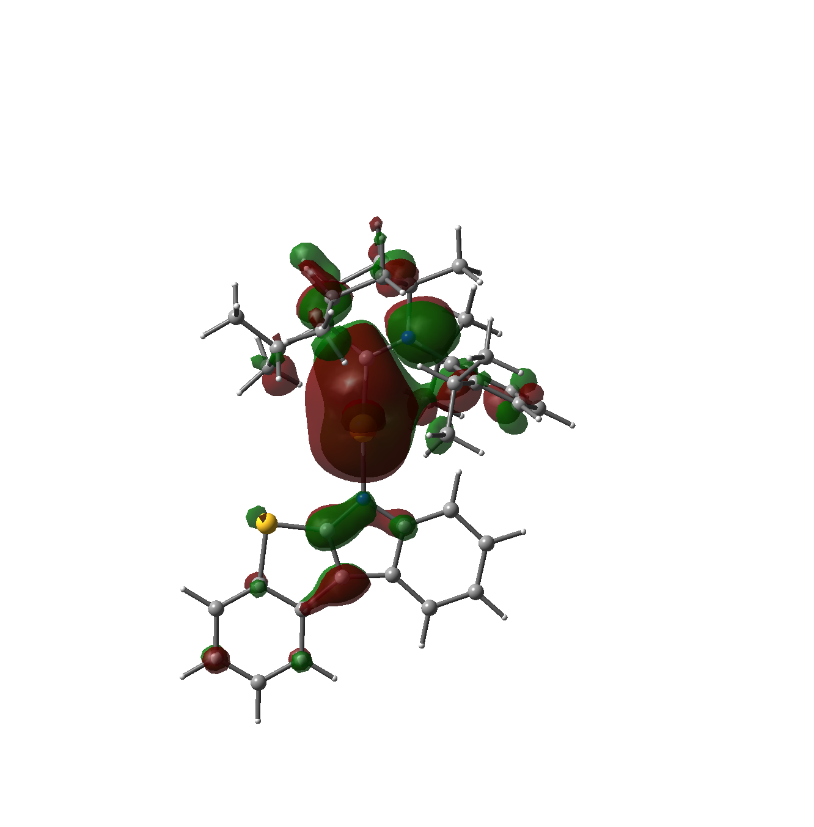 |
|  | T_2_ | 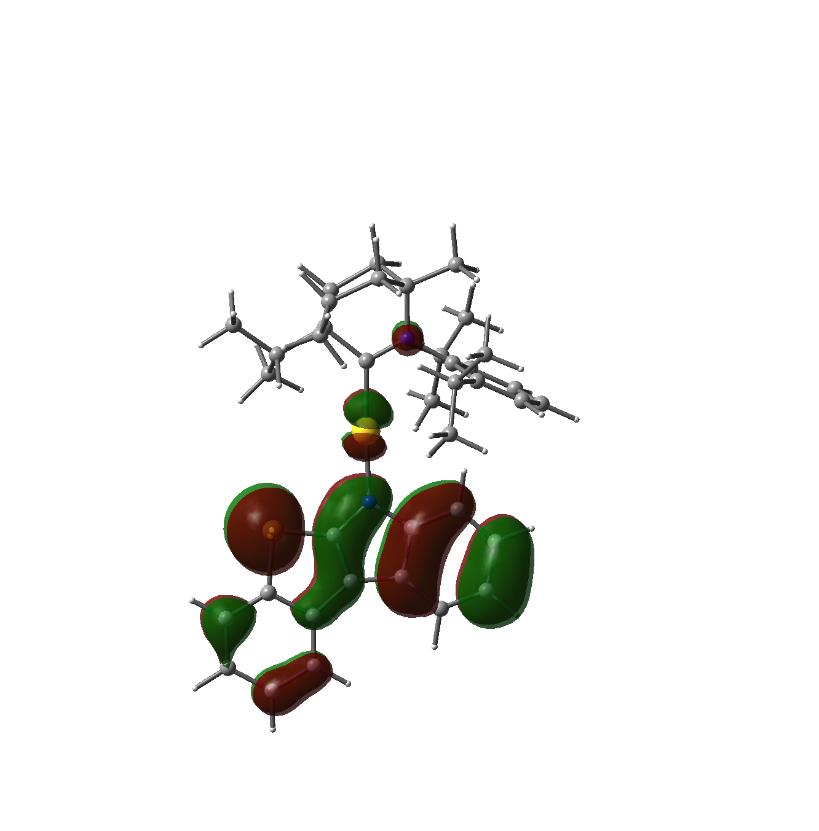 | 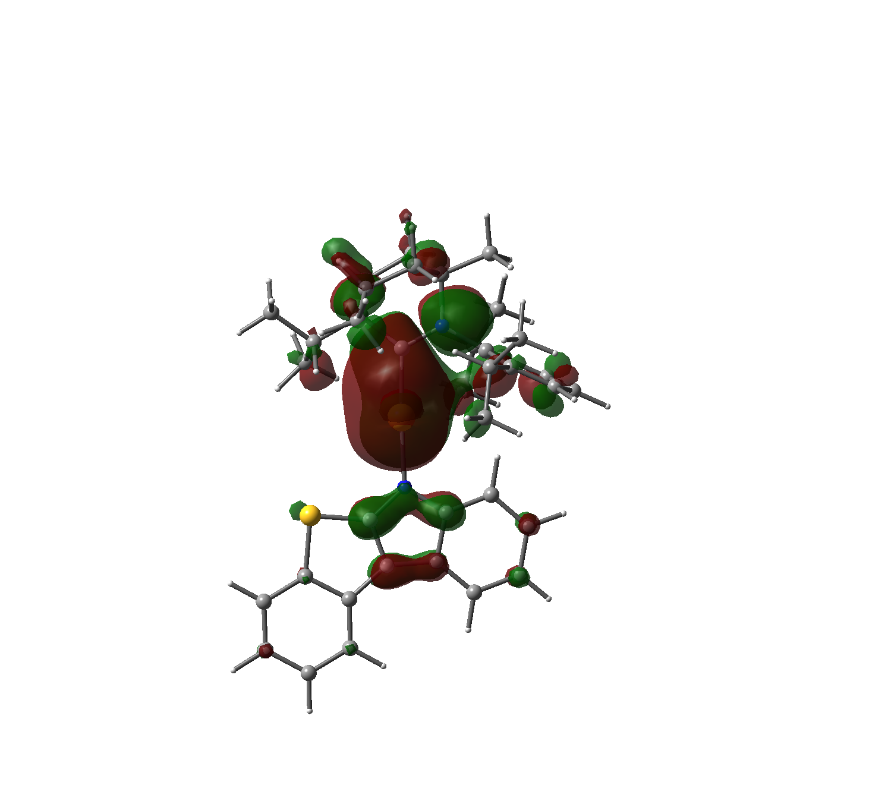 |
|  | T_3_ | 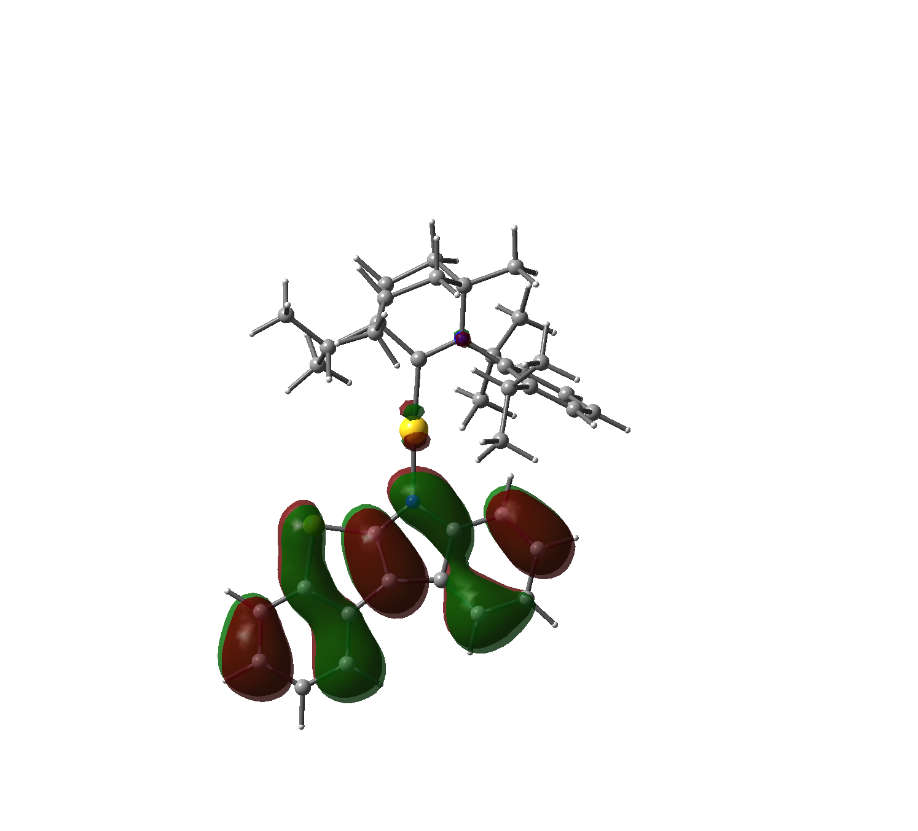 | 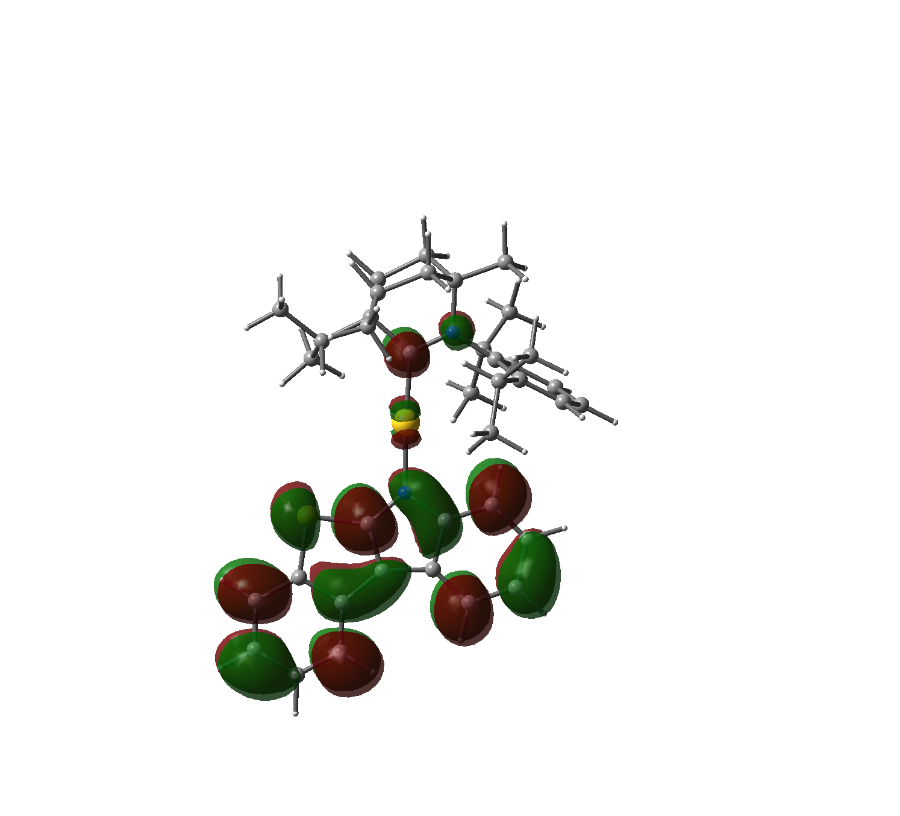 |

**Table S9.** Spin orbit coupling matrix elements (SOCME, cm^-1^) between excited singlet and triplet states.

|  | State | | Component | | | HSOC (cm^-1^) |
| --- | --- | --- | --- | --- | --- | --- |
|  | T | S | Z | X | Y |  |
| **BiCAuBFI** | 1 | 0 | -20.93 | -23.38 | -55.78 | 64.0 |
|  | 2 | 0 | 40.69 | -20.41 | -67.23 | 81.2 |
|  | 3 | 0 | -2.80 | 12.35 | 20.15 | 23.8 |
|  | 1 | 1 | -7.02 | -8.50 | -5.03 | 12.1 |
|  | 2 | 1 | -24.79 | 7.15 | -6.50 | 26.6 |
|  | 3 | 1 | -12.98 | -23.47 | 4.22 | 27.2 |
|  | 1 | 2 | -26.90 | 6.73 | 3.95 | 28.0 |
|  | 2 | 2 | -4.29 | 5.87 | 10.48 | 12.8 |
|  | 3 | 2 | 4.22 | 2.83 | 1.74 | 5.4 |
|  | 1 | 3 | 7.26 | 277.29 | -97.99 | 294.2 |
|  | 2 | 3 | -0.06 | -14.98 | 5.13 | 15.8 |
|  | 3 | 3 | -3.54 | -5.88 | -2.52 | 7.3 |
| **BiCAuBTI** | 1 | 0 | -14.75 | -23.70 | 41.13 | 49.7 |
|  | 2 | 0 | 26.49 | -18.16 | 28.51 | 42.9 |
|  | 3 | 0 | 6.70 | 11.33 | -11.88 | 17.7 |
|  | 1 | 1 | 6.46 | 9.00 | -2.84 | 11.4 |
|  | 2 | 1 | 17.16 | -2.92 | -3.62 | 17.8 |
|  | 3 | 1 | 7.09 | 25.88 | 3.95 | 27.1 |
|  | 1 | 2 | 18.08 | -3.71 | 2.19 | 18.6 |
|  | 2 | 2 | 0.50 | -5.55 | 4.00 | 6.9 |
|  | 3 | 2 | -2.60 | 1.24 | 2.44 | 3.8 |
|  | 1 | 3 | 16.40 | 332.86 | 168.76 | 373.6 |
|  | 2 | 3 | -1.55 | -24.52 | -16.60 | 29.7 |
|  | 3 | 3 | -5.46 | -30.63 | -21.96 | 38.1 |

**OLED fabrication and characterization**

**Table S10.** Photoluminescent properties of **BiCAuBFI** and **BiCAuBTI** in x% by weight mCP film at room temperature.

| mCP  :BiCAuBFI x wt% | *λ*_em,PL_^a^  (nm) | *τ*  (ns) | *Φ*_PL_^b^  (%) | *Θ*_h_^c^  (%) | mCP  :BiCAuBTI x wt% | *λ*_em,PL_^a^  (nm) | *τ*  (ns) | *Φ*_PL_^b^  (%) | *Θ*_h_^c^  (%) |
| --- | --- | --- | --- | --- | --- | --- | --- | --- | --- |
| 5 | 487 | 428 | 61.5 | 70 | 5 | 482 | 652 | 61.7 | 72 |
| 10 | 487 | 438 | 57.9 | 75 | 10 | 482 | 619 | 61.3 | 72 |
| 100 (neat) | 502 | 334 | 43.5 | 78 | 100 (neat) | 482 | 571 | 61.3 | 78 |

^a^ PL emission spectrum peak wavelength. ^b^ Absolute PLQYs. ^c^ Molecular horizontal dipole orientation.


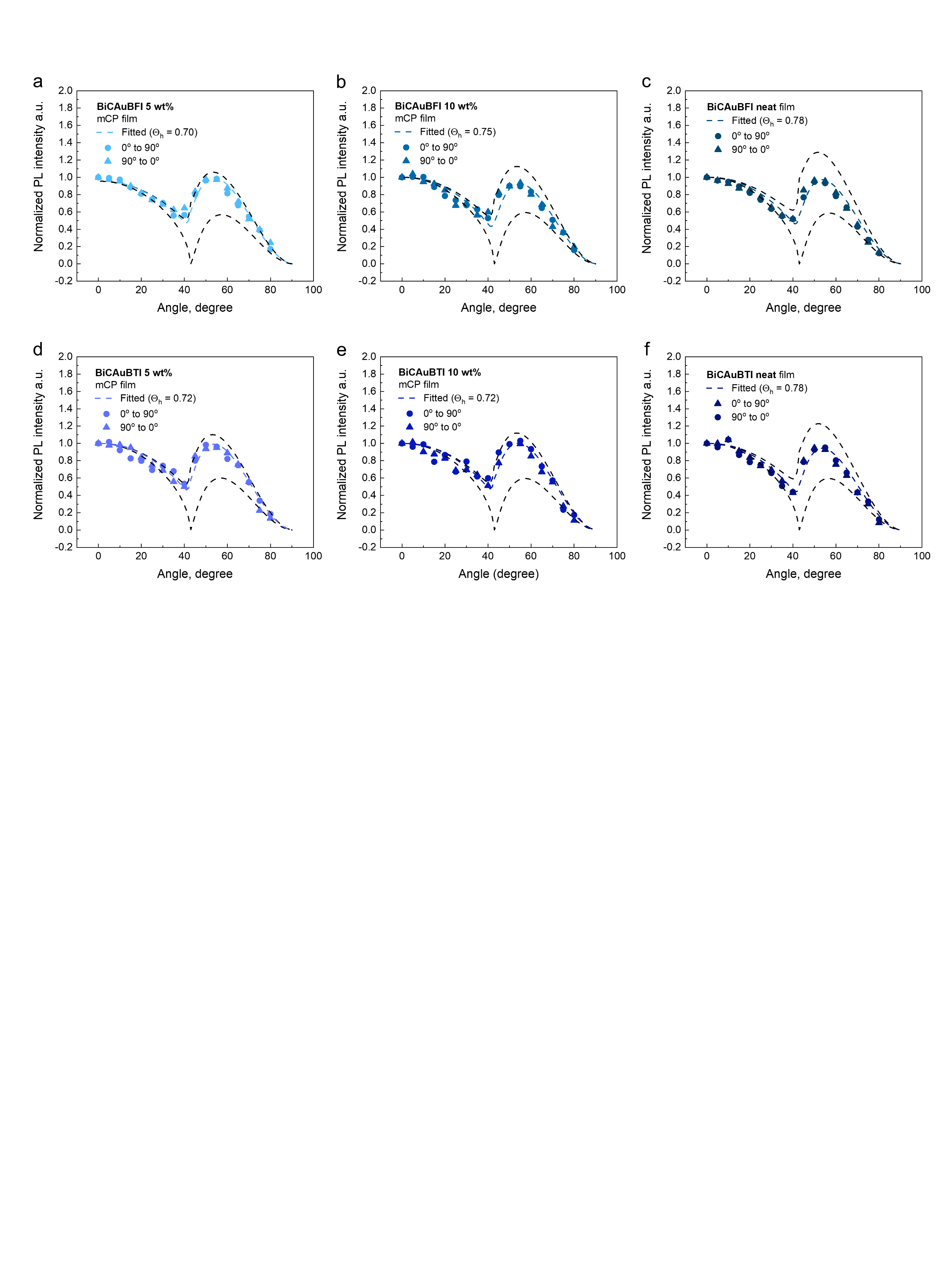


**Figure S14.** (a-f) Angle-dependent PL intensity of x% (x = 5, 10, 100 (neat)) doped thin-films of complexes **BiCAuBFI** and **BiCAuBTI** in 1,3-bis(N-carbazolyl)benzene (mCP) host at room temperature.


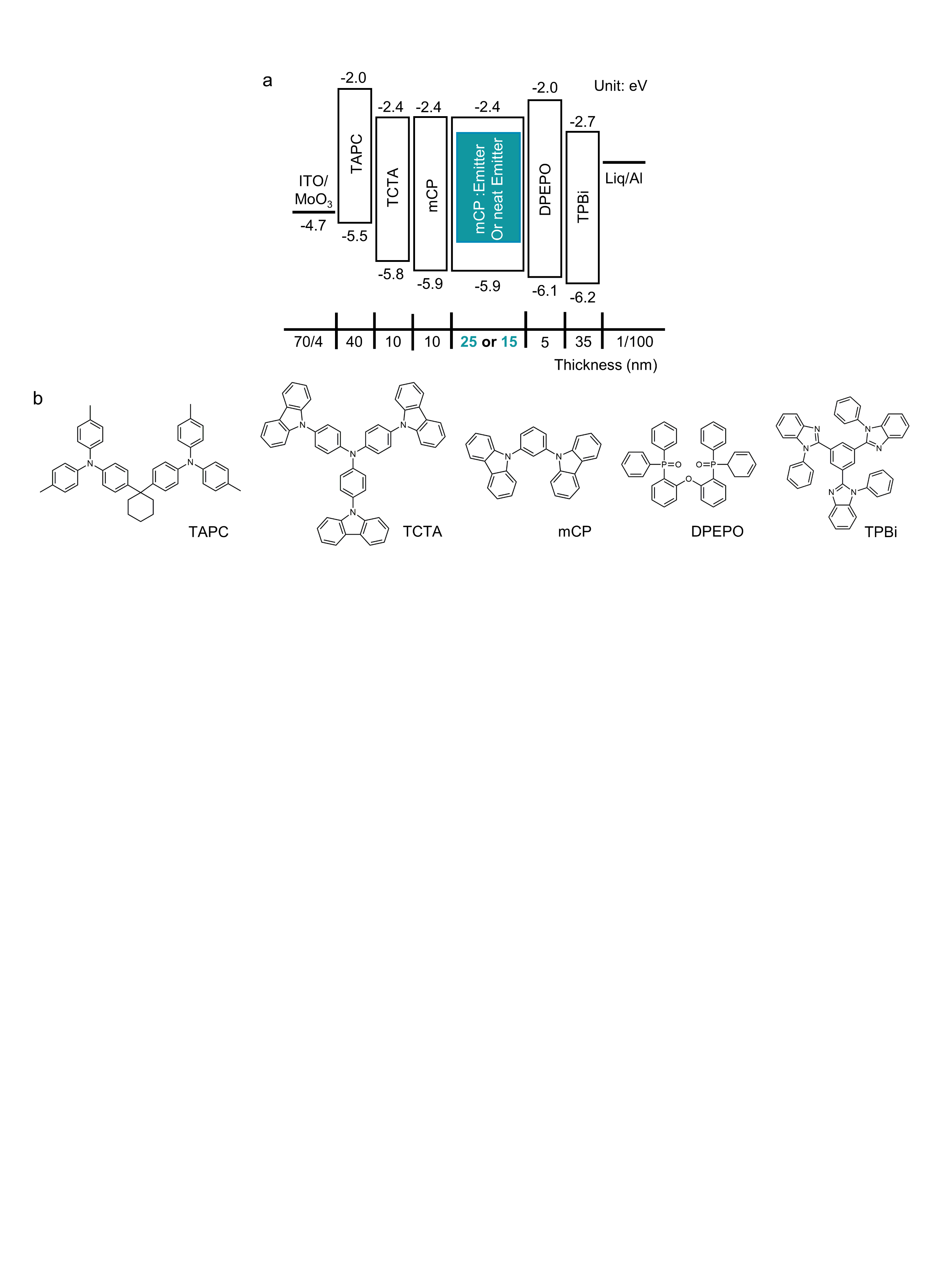


**Figure S15.** (a) Device structure and energy level diagram of CMA complex blue OLEDs. (b) Chemical structure of the transporting layer.


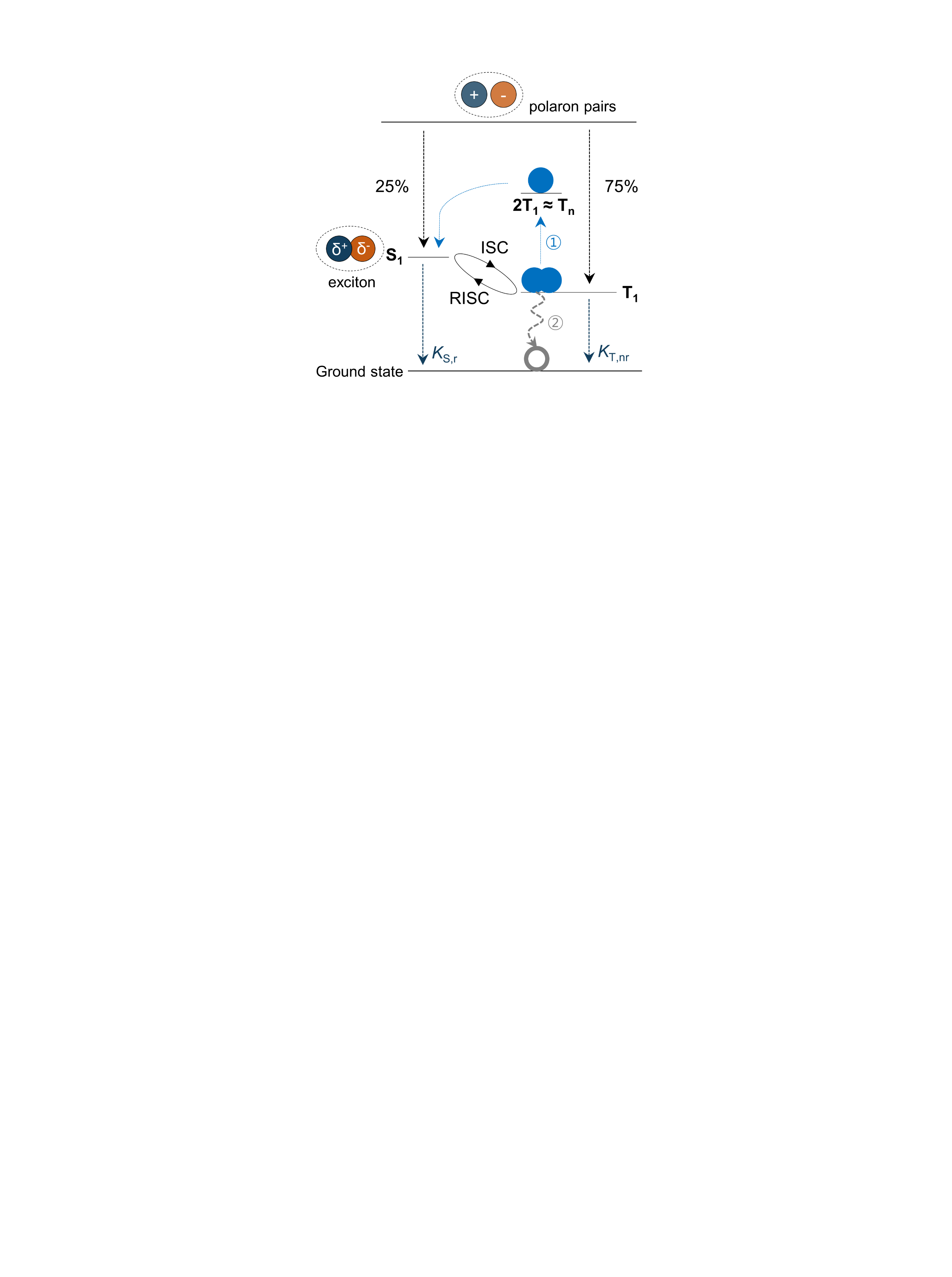


**Figure S16.** Schematic image of triplet-triplet collision. ①The triplet-triplet annihilation (TTA)-induced fluorescence. ②The triplet excitons quenching through triplet-triplet collision.


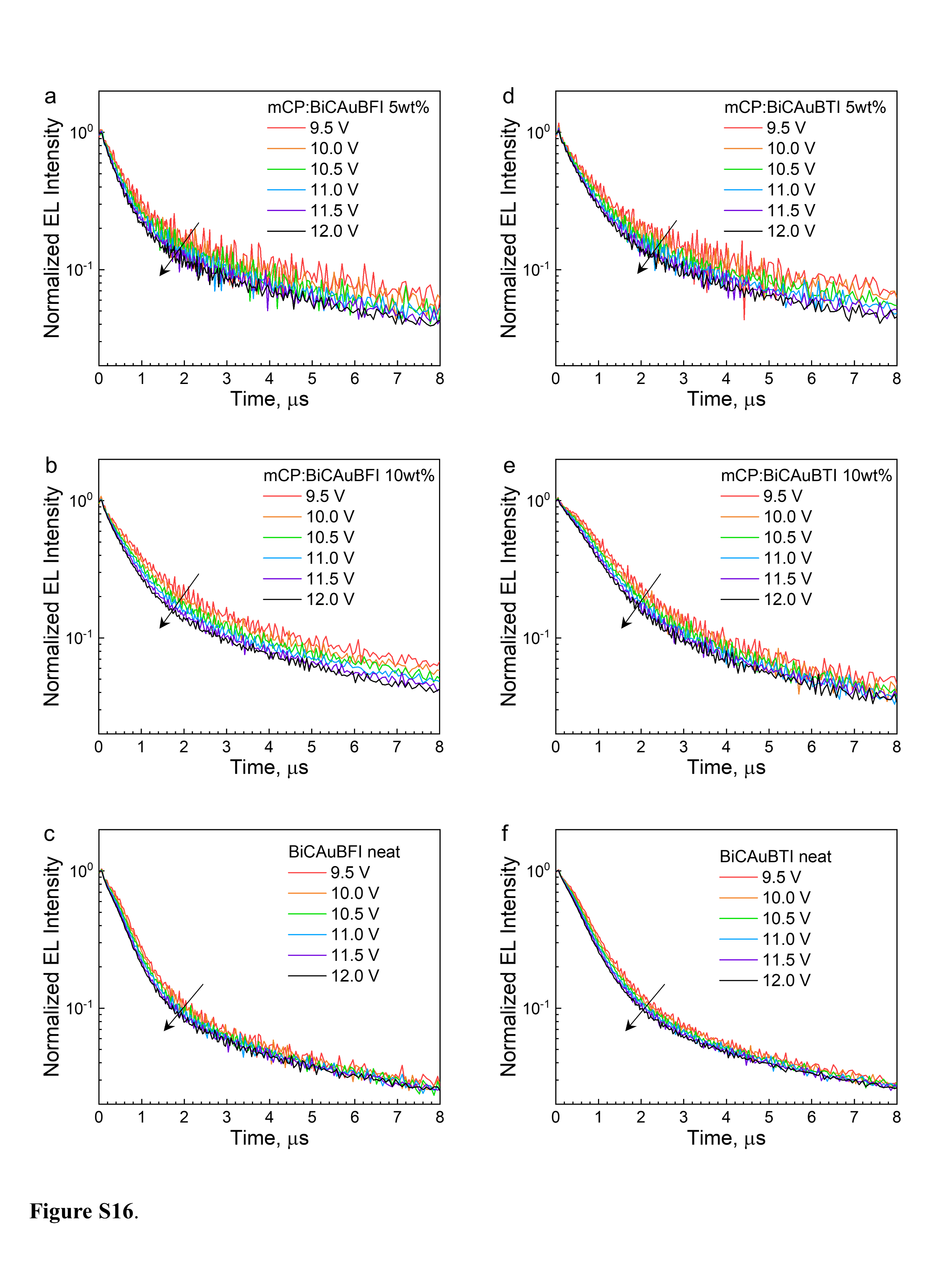


**Figure S17.** Time-resolved EL decay curves measured at different voltages (9.5–12.0 V): (a–c) mCP:BiCAuBFI (5 wt%, 10 wt%, and neat film, respectively) and (d–f) mCP:BiCAuBTI (5 wt%, 10 wt%, and neat film, respectively).


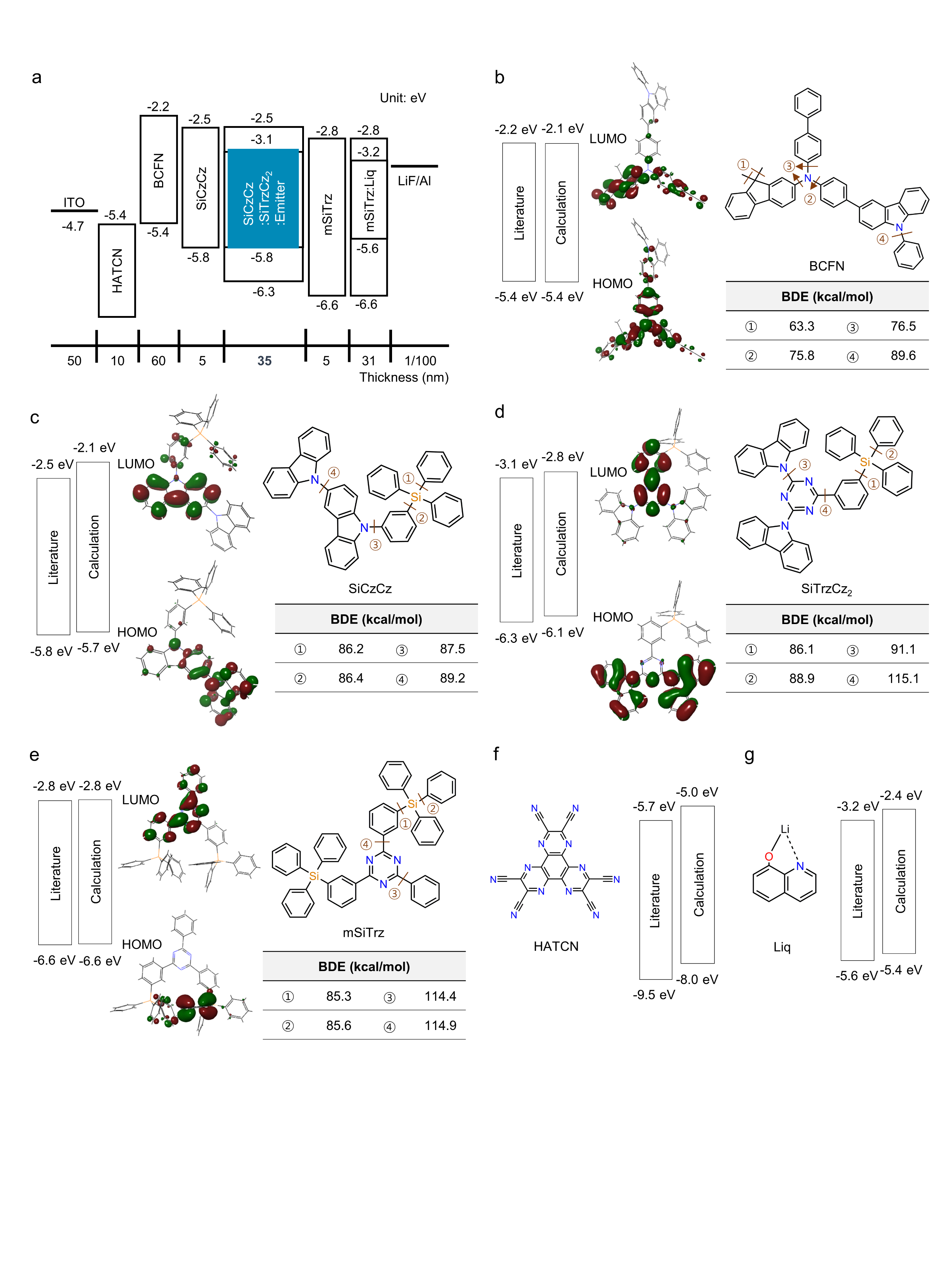


**Figure S18.** (a) Device structure and energy level diagram of CMA complex blue OLEDs with better operating stability. (b-g) Chemical structure of the transporting layer and calculated bond dissociation energies.


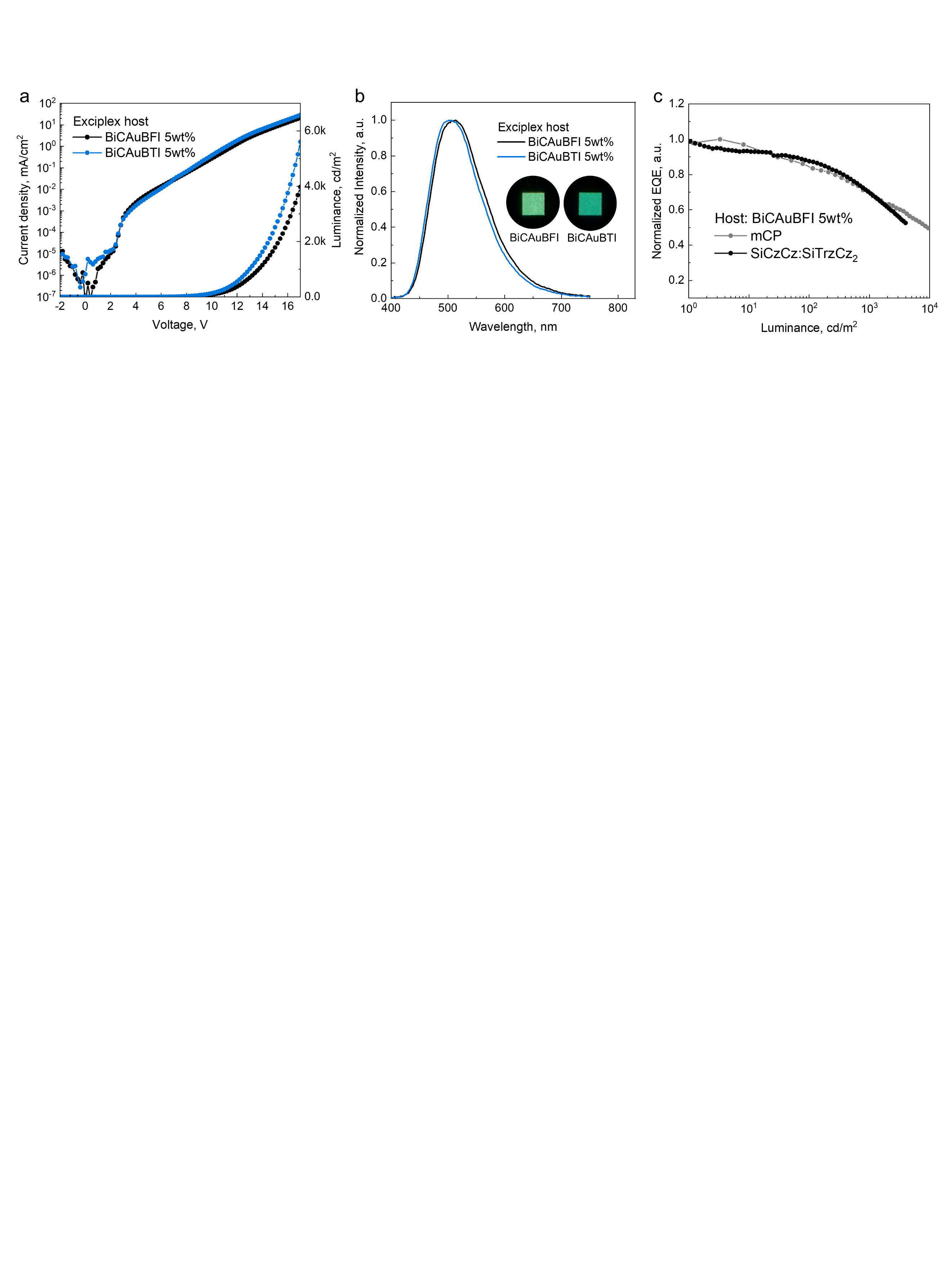


**Figure S19.** Electroluminescent characteristics of CMA complex blue OLEDs with better operating stability: emission layer (EML) is 5wt% doped thin-films of complexes **BiCAuBFI** and **BiCAuBTI** in SiCzCz:SiTrzCz_2_ exciplex host. (a) Current density-voltage-luminance (*J*-*V*-*L*) characteristics of the blue OLEDs. (b) EL spectra and photograph of the blue OLEDs. (c) normalized External quantum efficiency-luminance (EQE-*L*) characteristics of the blue OLEDs.

**Table S11.** Electroluminescent characteristics of CMA complex blue OLEDs with better operating stability^a^.

| Emission layer | *V*_on_^b^  (V) | *V*_op_^c^  (V) | C.E.^d^  (cd/A) | EQE^e^  (%) | *λ*_em,EL_^f^  (nm) | CIE (x,y)^g^ |
| --- | --- | --- | --- | --- | --- | --- |
| SiCzCz:SiTrzCz_2_  :BiCAuBFI 5 wt% | 3.6 | 13.8 | 36.7 / 24.7 | 13.7 / 9.3 | 503 | (0.267,0.468) |
| SiCzCz:SiTrzCz_2_  :BiCAuBTI 5 wt% | 3.8 | 13.0 | 39.4 / 27.7 | 14.7 / 10.3 | 502 | (0.249, 0.448) |

^a^ glass/ ITO (70 nm)/ HATCN (10 nm)/ BCFN (60 nm) / SiCzCz (5 nm)/ SiCzCz:SiTrzCz2:emitter 5wt% (6:4, 35 nm)/ mSiTrz (5 nm)/ mSiTrz:Liq (1:1, 31 nm)/ LiF (1 nm)/ Al (100 nm) ^b^ Applied voltage at a luminance of 1 cd/m^2^. ^c^ Applied voltage at a luminance of 1,000 cd/m^2^. ^d^ Maximum current efficiency: maximum, then the value at 1,000 cd/m^2^. ^e^ External quantum efficiency: maximum, then the value at 1,000 cd/m^2^. ^f^ EL emission spectrum peak wavelength. ^g^ Color coordinates (CIE 1931) at maximum luminance.

**References:**

1. Konidena, R. K.; Lee, K. H.; Lee, J. Y., 6H-Benzo[4,5]thieno[2,3-b]indole as a novel donor for efficient thermally activated delayed fluorescence emitters with EQEs over 20%. *J. Mater. Chem. C*, **2019**, *7*, 13912–13919. [↑](#endnote-ref-1)
2. Chotard, F.; Sivchik, V.; Linnolahti, M.; Bochman, M.; Romanov, A. S., *Chem. Mater.* **2020**, *32*(14), 6114–6122. [↑](#endnote-ref-2)
3. *Programs CrysAlisPro, Oxford Diffraction Ltd., Abingdon, UK (2010)*. [↑](#endnote-ref-3)
4. Sheldrick, G., SHELXT - Integrated space-group and crystal-structure determination. *Acta Cryst. A* **2015,** *71* (1), 3-8. [↑](#endnote-ref-4)
5. Sheldrick, G., Crystal structure refinement with SHELXL. *Acta Cryst. C* **2015,** *71* (1), 3-8. [↑](#endnote-ref-5)
6. Dolomanov, O. V.; Bourhis, L. J.; Gildea, R. J.; Howard, J. A. K.; Puschmann, H., OLEX2: a complete structure solution, refinement and analysis program. *J. Appl. Cryst.* **2009,** *42* (2), 339-341. [↑](#endnote-ref-6)
7. Guzei, I. A., An idealized molecular geometry library for refinement of poorly behaved molecular fragments with constraints. *J. Appl. Cryst.* **2014,** *47* (2), 806-809. [↑](#endnote-ref-7)
8. Gritzner, G.; Kůta, J. Recommendations on reporting electrodepotentials in nonaqueous solvents: IUPC commission on electro-chemistry. *Electrochim. Acta*, 1984, 29, 869−873. [↑](#endnote-ref-8)
9. F. Furche and D. Rappoport, Density functional methods for excited states: equilibrium structure and electronic spectra. In Computational Photochemistry; M. Olivuccim, Ed.; Elsevier: Amsterdam, 2005; pp. 93–128. [↑](#endnote-ref-9)
10. G. M. J. Peach and D. J. Tozer, *J. Phys. Chem. A,* 2012, **116**, 9783–9789. [↑](#endnote-ref-10)
11. H. S. Yu, X. He, S. L. Li and D. G. Truhlar, *Chem. Sci*., 2016, **7**, 5032–5051. [↑](#endnote-ref-11)
12. F. Weigend, M. Häser, H. Patzelt and R. Ahlrichs, *Chem. Phys. Lett.,* 1998, **294**, 143–152. [↑](#endnote-ref-12)
13. F. Weigend and R. Ahlrichs, *Phys. Chem. Chem. Phys.,* 2005, **7**, 3297–3305. [↑](#endnote-ref-13)
14. D. Andrae, U. Haeussermann, M. Dolg, H. Stoll and H. Preuss, *Theor. Chim. Acta*, 1990, **77**, 123–141. [↑](#endnote-ref-14)
15. F. Chotard, A. S. Romanov, D. L. Hughes, M. Linnolahti and M. Bochmann, *Eur. J. Inorg. Chem.,* 2019, 4234–4240. [↑](#endnote-ref-15)
16. A. S. Romanov, S. T. E. Jones, Q. Gu, P. J. Conaghan, B. H. Drummond, J. Feng, F. Chotard, L. Buizza, M. Foley, M. Linnolahti, D. Credgington and M. Bochmann, *Chem. Sci*., 2020, **11**, 435–446.

    ^17^ B. de Souza, G. Farias, F. Neese, R. Izsák, *J. Chem. Theory Comput.* **2019**, *15*, 1896-1904.

    ^18^ D. A. Pantazis, X.-Y. Chen, C. R. Landis, F. Neese, *J. Chem. Theory Comput.* **2008**, *4*, 908-919.

    ^19^ T. Lu and F. J. Chen, *Comput. Chem*., 2012, **33**, 580–592.

    ^20^ Gaussian 16, Revision A.03, M.J. Frisch, G.W. Trucks, H.B. Schlegel, G.E. Scuseria, M.A. Robb, J.R. Cheeseman, G. Scalmani, V. Barone, G.A. Petersson, H. Nakatsuji, X. Li, M. Caricato, A.V. Marenich, J. Bloino, B.G. Janesko, R. Gomperts, B. Mennucci, H.P. Hratchian, J.V. Ortiz, A.F. Izmaylov, J.L. Sonnenberg, D. Williams-Young, F. Ding, F. Lipparini, F. Egidi, J. Goings, B. Peng, A. Petrone, T. Henderson, D. Ranasinghe, V.G. Zakrzewski, J. Gao, N. Rega, G. Zheng, W. Liang, M. Hada, M. Ehara, K. Toyota, R. Fukuda, J. Hasegawa, M. Ishida, T. Nakajima, Y. Honda, O. Kitao, H. Nakai, T. Vreven, K. Throssell, J.A. Montgomery, Jr., J.E. Peralta, F. Ogliaro, M.J. Bearpark, J.J. Heyd, E.N. Brothers, K.N. Kudin, V.N. Staroverov, T.A. Keith, R. Kobayashi, J. Normand, K. Raghavachari, A P. Rendell, J.C. Burant, S.S. Iyengar, J. Tomasi, M. Cossi, J.M. Millam, M. Klene, C. Adamo, R. Cammi, J.W. Ochterski, R.L. Martin, K. Morokuma, O. Farkas, J.B. Foresman and D.J. Fox, Gaussian, Inc., Wallingford CT, 2016.

    ^21^ F. Neese, *WIREs Comput. Mol. Sci.* **2022**, *12*, e1606. [↑](#endnote-ref-16)
